# A Comparison of 100 Human Genes Using an *Alu* Element-Based Instability Model

**DOI:** 10.1371/journal.pone.0065188

**Published:** 2013-06-03

**Authors:** George W. Cook, Miriam K. Konkel, Jerilyn A. Walker, Matthew G. Bourgeois, Mitchell L. Fullerton, John T. Fussell, Heath D. Herbold, Mark A. Batzer

**Affiliations:** 1 Department of Biological Sciences, Louisiana State University, Baton Rouge, Louisiana, United States of America; 2 Department of Bioengineering, Clemson University, Clemson, South Carolina, United States of America; 3 Electrochemical Materials, Louisiana Business and Technology Center, Baton Rouge, Louisiana, United States of America; 4 Albemarle Corporation, Pasadena, Texas, United States of America; Tel Aviv University, Israel

## Abstract

The human retrotransposon with the highest copy number is the *Alu* element. The human genome contains over one million *Alu* elements that collectively account for over ten percent of our DNA. Full-length *Alu* elements are randomly distributed throughout the genome in both forward and reverse orientations. However, full-length widely spaced *Alu* pairs having two *Alus* in the same (direct) orientation are statistically more prevalent than *Alu* pairs having two *Alus* in the opposite (inverted) orientation. The cause of this phenomenon is unknown. It has been hypothesized that this imbalance is the consequence of anomalous inverted *Alu* pair interactions. One proposed mechanism suggests that inverted *Alu* pairs can ectopically interact, exposing both ends of each *Alu* element making up the pair to a potential double-strand break, or “hit”. This hypothesized “two-hit” (two double-strand breaks) potential per *Alu* element was used to develop a model for comparing the relative instabilities of human genes. The model incorporates both 1) the two-hit double-strand break potential of *Alu* elements and 2) the probability of exon-damaging deletions extending from these double-strand breaks. This model was used to compare the relative instabilities of 50 deletion-prone cancer genes and 50 randomly selected genes from the human genome. The output of the *Alu* element-based genomic instability model developed here is shown to coincide with the observed instability of deletion-prone cancer genes. The 50 cancer genes are collectively estimated to be 58% more unstable than the randomly chosen genes using this model. Seven of the deletion-prone cancer genes, *ATM, BRCA1, FANCA, FANCD2, MSH2, NCOR1 and PBRM1*, were among the most unstable 10% of the 100 genes analyzed. This algorithm may lay the foundation for comparing genetic risks posed by structural variations that are unique to specific individuals, families and people groups.

## Introduction

The draft human genome is interspersed with approximately 45% of mobile element related repetitive sequence [1]. Advanced sequence analyses indicate that the repeat related portion of the genome may be as high as 69% [2]. Retrotransposons, which reproduce through a copy and paste mechanism, have generated the majority of this repetition. The human retrotransposon with the highest copy number is the *Alu* element. *Alu* elements have populated the human genome with over one million copies and account for over 10 percent of all human DNA [3].

Both by insertion and by recombination, *Alu* elements spawn genetic disease [4]–[7]. Over 100 studies link Alu elements to deletion-related diseases (Table S1). It has been suggested that the most damaging impact of mobile elements may not be their insertion into genes, but their potential interactions with each other. Such interactions could result in deletions, duplications, inversions and a host of more complex genomic structural changes [8]–[11]. *Alus* have also been associated with copy number variation breakpoints [12], [13]. The incidence of *Alu-Alu* interactions is further supported by studies highlighting *Alu-Alu* gene conversion events [14], [15]. The homogenization of neighboring *Alu* sequences in ostensibly healthy subjects is consistent with the theory that *Alu-Alu* interactions routinely occur in healthy cells [16], [17].

Recombinant inverted *Alu* pairs have been shown to be unstable in genetically engineered yeast experiments when separated by up to 100 base pair (bp) and are potential sources of chromosome instability when separated by up to 350,000 bp in humans [18]–[20]. Furthermore, fusions of inverted *Alu* pairs previously separated by 1–5 kb have been recently identified at the breakpoints of high copy number loci in cancer cells [13].

Previously we reported that full-length inverted *Alu* pairs (represented by the letter, I) were statistically underrepresented in the human genome when compared to full-length direct oriented *Alu* pairs (represented by the letter, D). The term, *Alu* pair exclusions (APEs), was used to describe this human I∶D *Alu* pair imbalance [18]. In this study we find that the lower number of inverted *Alu* pairs (when compared to direct *Alu* pairs) applies to all combinations of human *Alu* sizes. Additionally, we characterize this human *Alu* pair I∶D imbalance and construct a model for estimating relative human genome instability based upon the premise that the human *Alu* pair I∶D imbalance is generated as a consequence of inverted *Alu* pair instability.

This newly developed *Alu* induced instability model was used to compare the relative instabilities of 50 human cancer genes with 50 randomly selected genes from the human genome to experimentally validate the model. The cancer genes considered in this study were selected for their potential susceptibility to deletions based upon previous studies [21]–[23]. This selection criterion was adopted in order to maximize the model's opportunity to distinguish between these two groups of genes. Taken together, the model estimates that the deletion-prone cancer genes are 58% more unstable than the randomly chosen genes.

## Results

Each human gene resides within a unique landscape of *Alu* elements. The structures of these landscapes vary in attributes that include *Alu* density, clustering and orientation. Adding further to *Alu* landscape complexity is the number of exons and their spacings. Within these backdrops inverted *Alu* pairs are statistically less numerous than direct oriented *Alu* pairs. It has been hypothesized that this imbalance is primarily the consequence of deletions generated by interactions between inverted *Alu* pairs [18].

This hypothesis was tested by construction of an algorithm designed to estimate the risk that a gene's *Alu* landscape could potentially impose upon its coding sequence. The coding sequence risk was estimated by multiplying two independent probabilities. The first probability, the *Alu*-induced deletion risk, is the probability of the occurrence of an *Alu*-induced deletion. This deletion probability was estimated by characterization of the human genome-wide inverted *Alu* pair to direct *Alu* pair imbalance. The imbalance is described by the ratio of inverted to direct *Alu* pairs, I∶D. In this study, the statistically significant departure of the I∶D ratio below unity (p<0.05) is assumed to be a consequence of deletions that remove inverted *Alu* pairs from the genome. The predicted likelihood of a deletion arising from the instability of a given *Alu* pair is derived from the genome-wide I∶D imbalance pattern. This likelihood is estimated as a function of three parameters which are discussed later in this section. The second probability, the *Alu*-induced deletion size risk, is the risk that once a deletion is formed, it will be of sufficient size to extend into the coding region of the gene being evaluated. Deletion size risk is estimated using an algorithm constructed from recent studies describing the human indel size frequency distribution. Each of these two probabilities is discussed in greater detail later in this section.

This *Alu* element-based instability model was used to compare the relative stabilities of 50 human cancer genes with 50 randomly selected genes from the human genome. The cancer genes considered in this study were selected for their potential susceptibility to deletions [21]–[23]. This methodology was utilized to increase the likelihood that the model would be able to discriminate between these two groups of genes.

### Two-hit potential of *Alu* elements

**Figure 1:**
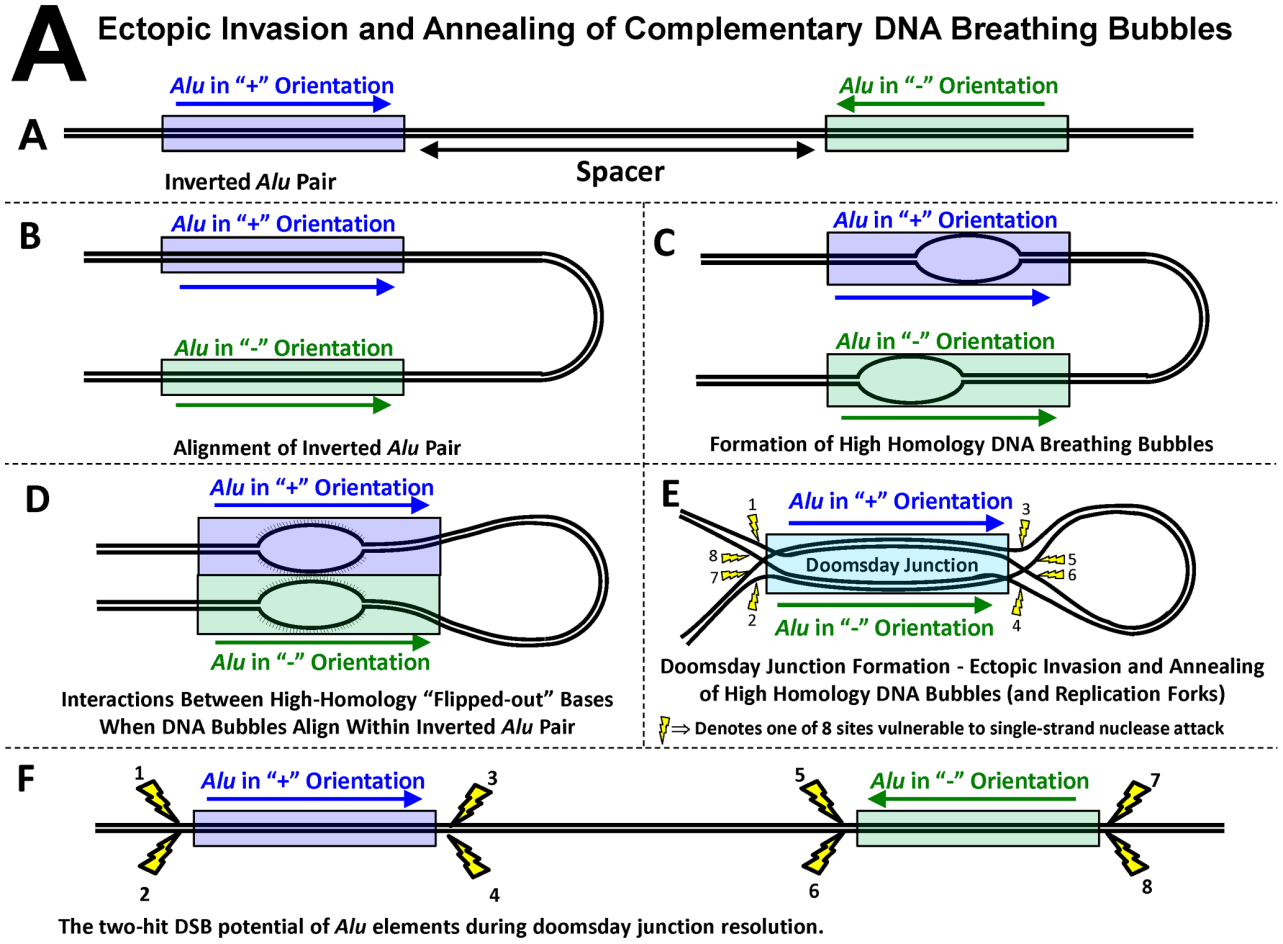
Proposed mechanism for formation and resolution of doomsday junction formed by ectopic invasion and annealing of complementary DNA breathing bubbles. (A) Two *Alu* elements in opposite orientations form an inverted *Alu* pair. (B) These inverted *Alu* pairs can align as high-homology regions. (C) DNA bubbles create short-lived sections of single-stranded DNA [25]. (D) The unbound bases within these bubbles are characterized by their flipping out from the centerline of the DNA strand [24]. Coincident passage of these bubbles within aligned *Alu* elements can create the opportunity for interactions between the flipped-out bases of the complementary DNA strands. (E) The ectopic invasion and annealing of single-stranded DNA associated with high-homology DNA bubbles could potentially extend to the entire length of the *Alu* elements. The hypothetical conformation created by this interaction is termed a doomsday junction. A similar interaction may also occur between high-homology replication forks and is described in Figure S1 and [18]. Eight segments of single-stranded DNA formed at the boundary of doomsday junctions create the opportunity for single-strand nuclease attack. These sites are illustrated as yellow lightning bolts. (F) As again illustrated by the yellow lightning bolts, each end of each *Alu* element involved in the doomsday junction is vulnerable to a double-strand break. This two-hit hypothesis for each *Alu* element was incorporated into the model's algorithm (see text).

The instability model assumes that each end of an *Alu* element is vulnerable to a double-strand break, DSB. These DSB sites are identified from the proposed DNA conformations associated with two mechanisms that have been suggested to explain human inverted *Alu* pair instability. These two mechanisms are characterized by the ectopic invasion and annealing of single-stranded DNA between high-homology DNA bubbles and/or replication forks [18]. Coincident DNA bubbles passing through aligned *Alu* elements may expose their complementary “flipped out” bases to one another [24], [25]. Complementary replication forks may also be susceptible to this type of interaction. Each pathway may result in the formation and subsequent resolution of a DNA conformation referred to as a doomsday junction. These two mechanisms are illustrated in Figures 1 and S1, respectively. Although we are unaware of other mechanisms that might also explain this imbalance, we readily acknowledge that they may exist. The ectopic DNA conformations described in these two figures are offered as possible explanations for the *Alu* pair I∶D imbalance phenomenon and they are used as a platform for constructing this instability model.

Figure 1E identifies the eight potential sites where a single-strand break could occur during the resolution of a doomsday junction. These sites (illustrated by yellow lightning bolts) are created at the periphery of the doomsday junction where each single strand of DNA transitions from the original DNA double helix to the ectopic conformation of the doomsday junction. These regions of single-stranded DNA may be susceptible to attack by single strand nucleases. If only one strand at the end of each *Alu* element is cut, the doomsday junction can likely resolve itself without damage to the original sequence. However, if both strands at the same end of either of the two inverted *Alu* elements are cut, a DSB can occur (Figure 1F). This potential for a DSB at each end of an *Alu* element forms the basis for the “two-hit hypothesis” for each *Alu* element considered by this instability model.

### Probability one – *Alu*-induced deletion risk

**Figure 2:**
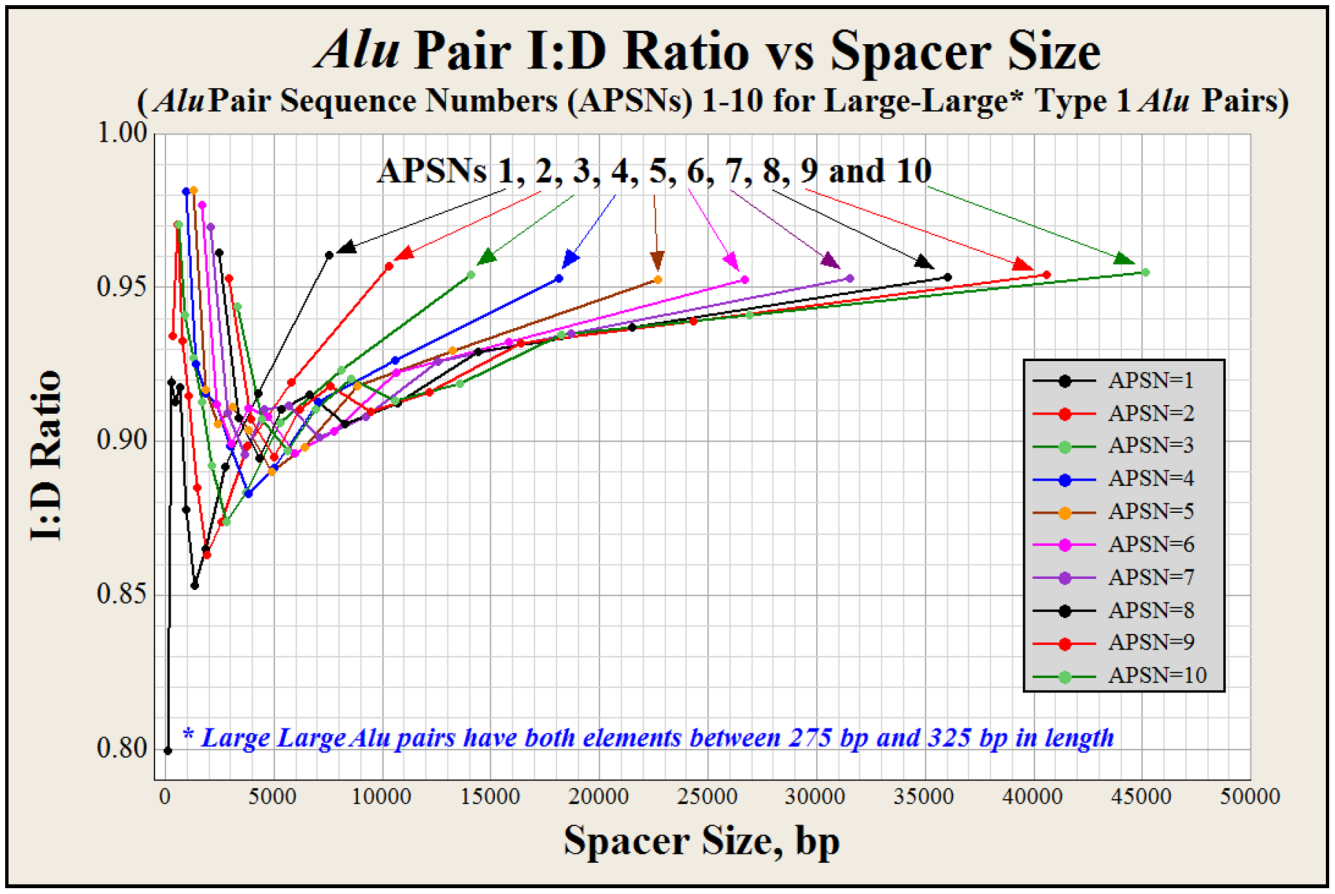
The *Alu* pair I∶D ratio versus spacer size for Type 1 *Alu* pairs for APSNs 1–10. A total of 10 points are used to construct each of the 10 APSN curves in this figure. These points represent the respective *Alu* pair I∶D ratios for 10, non-overlapping spacer size groupings. The first point (left to right) represents the I∶D ratio for the smallest five percent of spacer sizes. This point is followed by nine consecutively larger spacer size groupings. Each of these nine larger sized groupings contains 10% of the *Alu* pairs found within the respective APSN family. The I∶D ratio for each percentile group is plotted against its median spacer size, respectively (see Methods). This plot illustrates that the I∶D ratio is not a continuous function versus spacer size and may indicate the activity of different *Alu-Alu* interaction mechanisms (see text). These curves, along with their 5′ mirror images, make up ten of the 220 (APSNs ±1–110) curves that are collectively shown in Figure 3.

**Figure 3:**
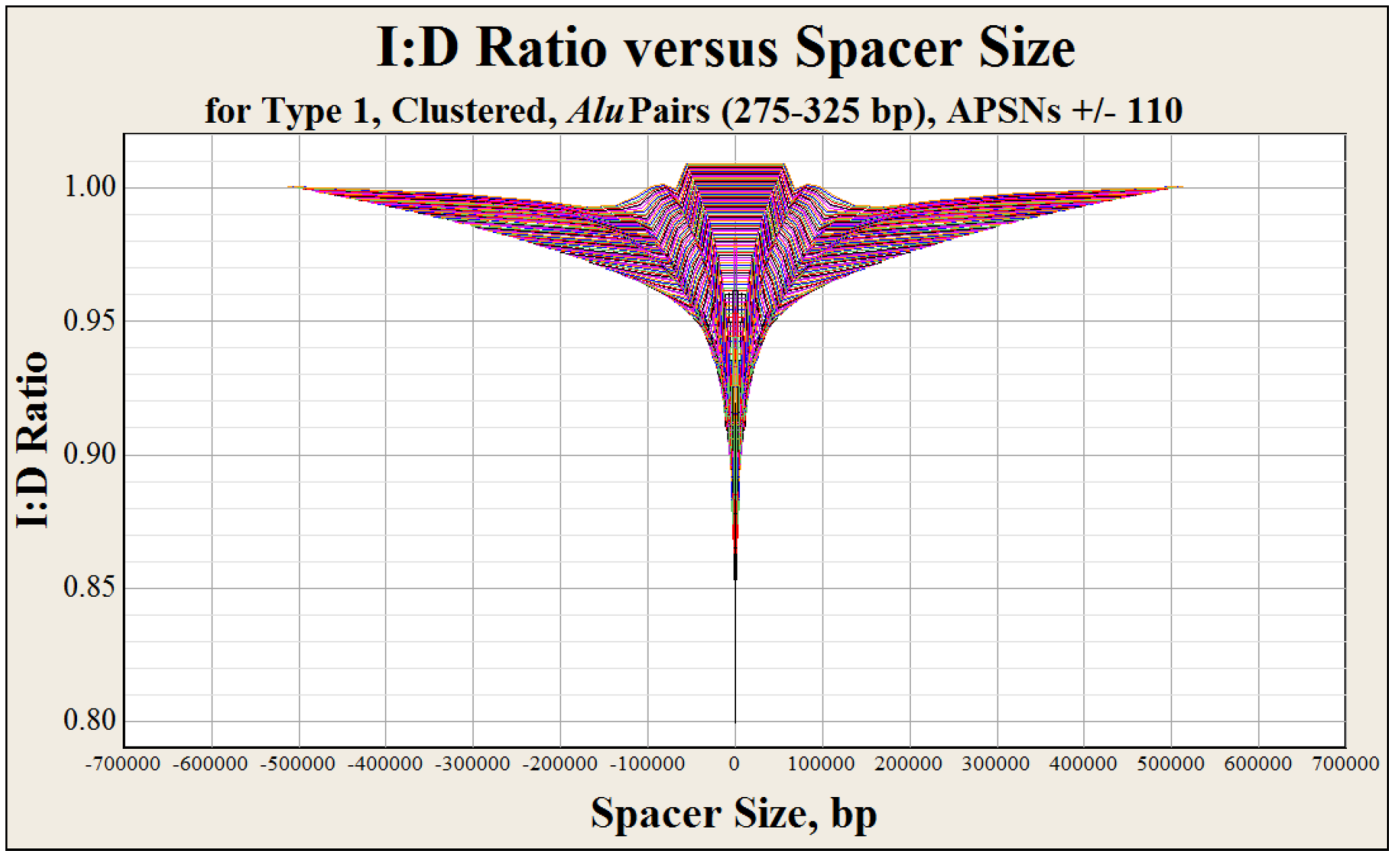
The *Alu* pair I∶D ratio versus spacer size for Type 1 *Alu* pairs for APSNs ±1–110. This figure illustrates the ±110 APSN curves for full-length (275–325 bp) Type 1 human *Alu* pairs. The individual curves in this figure are so closely spaced that they collectively appear as a surface. An expanded view of Type 1 APSN curves 1–10 is shown in Figure 2. Similar I∶D surfaces for Type 2 and Type 3 *Alu* pairs are shown in Figures S2A and S2B, respectively.

The *Alu*-induced deletion risk is the likelihood of a deletion arising from the resolution of a doomsday junction. The two-hit deletion potential of each *Alu* element results in the number of potential *Alu*-induced deletion sites within a given *Alu* landscape being twice the number of *Alu* elements. Three variables were found to significantly correlate with the *Alu* pair I∶D ratio; 1) spacer size, 2) the number of *Alu* elements within the spacer and 3) the clustering state of the each *Alu* pair (discussed in more detail, below). Figures 2, 3 and S2 express the human inverted to direct *Alu* pair ratio, I∶D ratio, as a function of these three variables The *Alu* pair I∶D ratio was not found to significantly correlate with *Alu* length (see Methods).

Figure 2 provides a detailed view of how the I∶D ratio varies with spacer size for APSNs 1–10 for Type 1 *Alu* pairs. Each of these 10 curves is plotted along 10 data points. These 10 data points represent the I∶D ratio for 10 fitted spacer size groupings from Table S2. These 10 data points represent, from smallest to largest, the 2.5th, 10th, 20th, 30th, etc. through 90th percentiles of spacer sizes groupings (see Methods). The shape of the curves in these three figures illustrate that the *Alu* pair I∶D ratio is not a smooth function across the full range of spacer sizes. These curves are plotted along the median of spacer size for the ten spacer size percentile groupings for each of the respective *Alu* pair sequence numbers (APSNs). The APSN is the parameter that describes the number of *Alus* within the spacer of an *Alu* pair. The APSN for an *Alu* pair is the n+1 number of *Alu* elements residing with the spacer (see Methods).

Three possible mechanisms may explain the unusual shape of the human *Alu* pair I∶D ratio versus spacer size curves. Using the APSN1 curve in Figure 2 as a reference, these three mechanisms may be as follows; 1) between the 0th and 5th spacer size percentiles (centered at ∼100 bp), hairpin formation may be the predominant form of *Alu-Alu* interaction, 2) for the 10th (5th–15th), 20th (15th–25th) and 30th (25th–35th) spacer size percentiles (centered between ∼100 and ∼500 bp) DNA persistence (stiffness), may hinder inverted *Alu-Alu* interactions and 3) for spacer sizes between the 40th (35th–45th) and 90th (85th–95th) spacer size percentiles, DNA persistence appears to wane and the curve begins to progress toward unity.

Human *Alu*, LINE1 and SVA elements, frequently cluster together in groups where adjacent elements are separated by ≤50 bp [18]. Using this definition of clustering, four types of clustered *Alu* pairs can be described. These are identified as Types 0, Type 1, Type 2 and Type 3 *Alu* pairs. Type 0 *Alu* pairs (clustered together) have both *Alu* elements residing within the same cluster, Type 1 *Alu* pairs (clustered separately) have both *Alu* elements residing within different clusters, Type 2 *Alu* pairs (hemi-clustered) have only one of the two elements residing within a cluster and Type 3 *Alu* pairs (non-clustered) have neither element residing within a cluster (see Methods). Type 1, 2 and 3 *Alu* pairs exhibit distinctly different I∶D ratios and their stabilities must therefore be estimated separately (Figure S3). Type 0 *Alu* pairs are subject to strong orientational insertion bias and their instability has been estimated via experimental studies of *Alu* elements in yeast (see Methods and [18], [19]).

Figure 2 illustrates the I∶D ratio versus spacer size for Type 1 large-large (275–325 bp) *Alu* pairs for APSNs 1–10. Figure 3 is similar to Figure 2 and includes all APSNs (±110) containing at least one spacer size percentile with an I∶D ratio <0.995. I∶D ratios ≥0.995 do not provide statistical confidence that the I∶D ratio is below unity (see Methods). Figures S2A and S2B are similar to Figure 3 and show the I∶D ratio versus spacer size and APSN for Type 2 and Type 3 *Alu* pairs, respectively.

Using the I∶D ratio relationships illustrated in Figures 3 and S2, the model generates a predicted stability for each *Alu* element within a gene's *Alu* landscape. The predicted I∶D ratio is the predicted stability for the *Alu* pair. The contribution that an inverted *Alu* pair makes to the stability of each *Alu* element of that pair is obtained by taking the square root of that pair's predicted I∶D ratio. Likewise, the stability for one end of an *Alu* element is the fourth root of that *Alu* pair's predicted I∶D ratio. The overall stability of one end of an *Alu* element is the product of the fourth roots of all of the predicted I∶D ratios for each of the potential 220 inverted interactions (i.e., the grand product) that an *Alu* element might form with its ±110 *Alu* neighbors (see Methods).

Figure 3 reveals an unexpected excursion of the I∶D ratio above unity for the highest *Alu* density genomic regions. This excursion only exists for APSNs ≥65 and only for the most *Alu* dense regions of the genome (0–5^th^ spacer size percentile, Table S2). This high I∶D ratio may indicate that direct *Alu* pair recombination in these high *Alu* density regions of the genome may outpace the activity of inverted APE events.

### *Alu* landscapes

**Figure 4:**
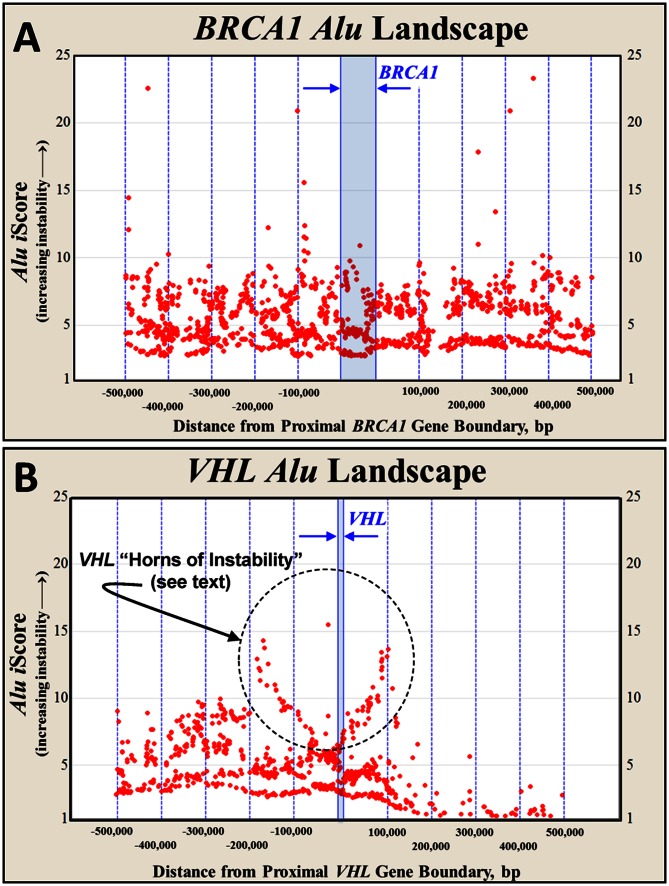
*Alu* landscapes for *BRCA1* and *VHL*. This figure characterizes the *Alu* landscapes within and 500 kbp, 5′ and 3′ of A) *BRCA1* and B) *VHL*. The midpoint for each *Alu* element is plotted against its respective instability score, *i*Score. Larger *i*Score values represent higher predicted *Alu* element instabilities (see text). Similar *Alu* landscapes for eight additional genes examined in this study are shown in figures S4A–H. These spans are twice the size of the ±250 kbp flanking landscapes which are considered to pose a risk for an exon damaging deletion (see text). These larger spans better illustrate the ebb and flow of *Alu*-related instability around each respective gene.

Each of the genes considered in this study were evaluated using the backdrop of *Alu* elements in which they reside. These *Alu* backdrops are referred to as *Alu* landscapes. Figure 4 illustrates the *Alu* landscapes around two of the deletion-prone cancer genes evaluated in this study, *BRCA1* and *VHL*. The vertical blue lines in each figure demarcate 100,000 bp distances from the respective end of each gene and the light blue region in the center of each diagram encompasses the respective gene's coding locus.

The respective instability score (*i*Score) for each *Alu* element is plotted along the vertical axis at the locus of each *Alu* element. These *i*Score values are the inverse of the *Alu* stabilities calculated using the algorithms developed from Figures 3 and S2. Higher *i*Score values represent higher *Alu* instabilities. The red dots signify the locus versus the iScore value for each element within the *Alu* landscape.

The *Alu* landscapes illustrated in Figure 4 span ±500,000 bp from the end of each gene. Similar landscapes are shown for eight additional genes in Figure S4. The instability model only includes those *Alus* residing within ±250,000 bp from the end of each gene (discussed in more detail, below). The larger landscapes provided in Figures 4 and S4 are shown to illustrate the ebb and flow of *Alu* instabilities across the genome. Approximately 0.3% of the human genome is represented in the 10 *Alu* landscapes shown in these two figures.

An average of 410 *Alu* elements reside within the +/− 250,000 bp landscapes of the genes examined in this study. These gene-specific *Alu* populations are not of sufficient size to detect inverted *Alu* pair stability with statistical confidence (see Methods). However, using the genome-wide human *Alu* population, it is possible to construct a statistically relevant model to estimate relative gene instabilities based upon each gene's respective *Alu* landscape. An additional insight into this model's relevance is that the human genome-wide I∶D imbalance is slightly over two percent (I∶D = 0.979) [18]. While this I∶D depression is statistically significant (p<0.05) the large majority of inverted *Alu* pairs likely remain in the human genome. Consequently, if the mechanisms that created this I∶D depression remain active, the genome-wide loss of slightly over two percent of the inverted *Alu* element population would likely do little to deter the continued activity of these mechanisms. Furthermore, wet bench experimental comparisons between orthologous chimpanzee and human inverted *Alu* pair loci reveal the chimpanzee-specific loss of inverted *Alu* pairs [18]. This analysis suggests that a portion of the loss of inverted *Alu* pairs may be of recent origin.

The panes in Figure S4A–S4E illustrate the *Alu* landscapes for the five deletion-prone cancer genes, *APC*, *ATM, BRCA1, MLH1 and MSH2*. The panes in Figure S4F–S4H describe the *Alu* landscapes for randomly chosen genes, *GDPD2, KEAP1 and SF3B3*. Among the 100 genes examined in this study, only two of the top 10 highest *Alu* density landscapes are associated with deletion-prone cancer genes, *ARID1A* and *BRCA1*. These two genes rank 8^th^ and 10^th^ in this list with *Alu* landscape densities of 1,322 and 1,309 *Alus* per mega base, respectively (see Table S3). The *Alu* element density across the human genome averages 381 *Alus* per mega base. The top five most *Alu* dense landscapes (all randomly selected genes) belong to *KEAP1, NCF1, NANOS3, OPRD1*, and *SET1* with *Alu* densities of 1,916, 1,783, 1,644, 1,534 and 1,525 *Alus* per mega base, respectively (see Table S4).

### Probability two – *Alu*-induced deletion size risk

**Figure 5:**
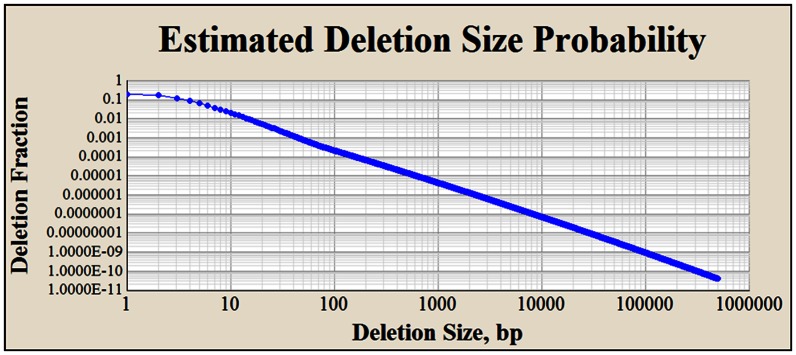
Estimated human deletion size frequency distribution. (A) This log-log (base 10) plot estimates the relative distribution of deletion sizes within the human genome. The curve was constructed from two different studies and predicts that 95% of deletions are ≤50 bp in size and 99% of deletions are ≤445 bp [26], [27]. When combined with the two-hit hypothesis for *Alu* elements (see Figure 1F and text), this curve suggests that the two ends of an *Alu* element pose specific and different risks to an exon's coding region.

Human genome indel size frequency distributions from two previous studies provide a glimpse into the shape of the overall human deletion size frequency distribution [26], [27]. A hybrid deletion size frequency model was developed from these studies and is shown in Figure 5. The sum of the 500,000 deletion probabilities shown in this figure equals 1.0. This hybrid model is used to estimate the relative deletion size risks that arise from inverted *Alu*-induced DSBs (See Methods). The shape of the curve in Figure 5 reflects a deletion size frequency distribution where 95 percent of deletions are ≤50 bp. The maximum Figure 5 deletion size of 500,000 bp was chosen because this size deletion has a risk of occurrence that is less than one billionth of the risk predicted for a 1 bp deletion. This model assumes that deletions extend equidistant from an initiating DSB. Consequently, the maximum distance from which an individual *Alu* element is considered to pose a deletion risk to a coding exon is 250,000 bp (250,000 bp×2 = 500,000 bp). In addition to considerations for maximum deletion size, additional flanking sequence must be examined within an *Alu* landscape to accommodate for the possibility that inverted *Alu* pairs can interact when separated by up to 421,000 bp. This is the spacer size (in Figure 3) that intersects with an I∶D ratio of 0.995. This I∶D ratio is statistically lower than unity (p≤0.05, see Methods). Therefore, an *Alu* element that is separated by as much as 671,000 bp from a coding exon could potentially threaten the coding integrity of that exon. At this distance from a coding exon, an *Alu* element could conceivably interact with a second *Alu* separated by only 250,000 bp from the same exon (spacer size between the two *Alus* = 671,000 bp - 250,000 bp = 421,000 bp). This interaction could potentially generate a DSB at the second *Alu* that could possibly extend into the coding exon.

### Relative gene stabilities

**Figure 6:**
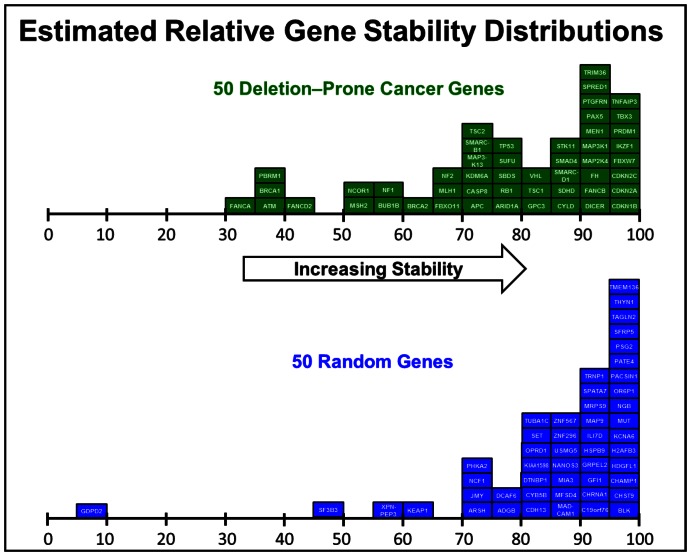
Distributions of estimated relative instabilities for 50 deletion-prone cancer genes and 50 randomly chosen genes. This diagram provides separate histograms that describe the relative instabilities of the 50 deletion-prone genes and the 50 randomly selected genes, respectively. The values in these histograms are the unadjusted outputs from the *Alu* element-based instability model algorithm. These stabilities are also provided in Tables S3 and Table S4, respectively. Note that the least stable of all 100 genes is the randomly selected gene, *GDPD2*. This low stability is the result of the putative exonized *Alu* that occurs in variant 1 of *GDPD2's* 12^th^ exon (see text).

The relative stability of a gene for the purpose of this study is defined as the relative likelihood that a coding exon will not be breached by a deletion. The determination of this stability must consider the collective deletion risks along with the respective deletion size risks posed by all potential DSB sites generated within a gene's *Alu* landscape. More specifically, the overall stability of a gene is the multiplied product (grand product) of the individual *Alu* element contributions to that gene's stability within its *Alu* landscape (see Methods). The required calculations to determine this stability are extensive. Estimation of the stability of *BRCA1*, because of its large *Alu* landscape, requires 171,225 consecutive calculations. As can be seen from Table S3, *BRCA1* has 761 *Alu* elements residing within its intronic regions and the 250,000 bp flanking regions, 5′ and 3′ of the gene. The majority of these calculations are associated with the 220 potential *Alu* pair interactions for each of these 761 *Alu* elements. The sheer number of required consecutive calculations raised concerns that significant adjustments would be required for proper interpretation of the raw output from the model. This concern did not materialize. The individual gene stabilities plotted in Figure 6 are the unadjusted output stability values from the model.

The uppermost histogram in Figure 6 is a distribution of the raw stabilities of the 50 deletion-prone genes taken directly from the model. The bottom histogram is a distribution of the raw stabilities of the 50 randomly selected genes. Lower values represent greater instability. Tables S3 and Table S4 list the individual gene stabilities. For reference, this instability model would generate a stability of 100 for a gene residing within an *Alu*-free landscape. The average unadjusted stabilities of the deletion-prone cancer genes and randomly chosen genes from Tables S3 and S4 are 77.7% and 85.9%, respectively. The deletion-prone cancer genes, therefore, have 58% greater likelihood of a deletion insult than that of the randomly chosen genes.
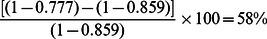
This likelihood increases to 78% when *GDPD2*, the randomly chosen gene with an exonized *Alu* element, is excluded from the list of random genes (discussed in more detail, below).

Only one cancer gene, *IKZF1*, was among the most stable 10% of the 100 genes analyzed, while seven deletion-prone cancer genes, *FANCA, NCOR1, BRCA1, PBRM1, ATM, FANCD2* and *MSH2* were among the most unstable 10% (10) of the 100 genes analyzed (Tables S3 and S4). The top 10% most stable genes contain an average of 4 coding exons, versus an average coding exon count of 31 for the 10% most unstable genes (Tables S3 and S4). Individual least squares correlations were subsequently performed using 1) exon number, 2) *Alu* population within each gene's *Alu* landscape and 3) gene coding length to determine the extent to which these parameters can be used to predict the estimated relative gene stabilities determined in this study. These three correlations were made using the combined set of 100 genes found in Tables S3 and S4. The best predictor of the model's estimated relative gene stability among these three variables was found to be gene exon number followed by the *Alu* population within the +/− 250,000 bp landscape followed by gene coding region length. Using a least squares regression for these three variables versus this model's estimated gene stabilities generated adjusted R^2^ values of 41.7%, 23.2% and 1.6%, respectively. These lower adjusted R^2^ values suggest that the algorithms used in this instability model provide complexity which cannot be accurately estimated with a single variable. This is consistent with the view that this study's methodology is a new approach for accessing the *Alu* element contribution to estimating relative gene instabilities.

The least stable of all 100 genes is the randomly selected gene, *GDPD2*. The low relative stability of *GDPD2* (7.1%, see Table S4) results from a putative exonized *Alu* that occurs in variant 1 of *GDPD2's* 12^th^ exon. Four different variants of this gene are represented in the UCSC genome browser. The absence of this exon in the other three variants is consistent with this predicted instability. This *Alu* element-based instability model considers an exonized *Alu* element as the most unstable form of structural variation within a gene's coding region. Therefore, in addition to the disruption of coding sequence associated with an *Alu* insertion into an exon, subsequent disruption may also ensue because of the high potential for small deletions to occur at the ends of the *Alu* element. Both of these mechanisms may help explain the scarcity of exonized *Alus*. The potential risk of an exon-damaging deletion originating from the end of a nearby *Alu* element is consistent with the observed scarcity of *Alus* and other transposable elements within 50 bp of human exons [28], [29].

An examination of the variation in relative gene instabilities with respect to variation in the deletion size frequency distribution was also conducted. This evaluation was performed by varying the ≤50 bp deletion size frequency between 90 and 99 percent in increments of one percent (Figure S5). While this analysis resulted in significant changes in absolute gene instabilities, the relative instabilities between most genes were unaltered. Exceptions to this observation occurred for *ATM* and *CASP8*. These have the two closest *Alu* elements located within 5 and 7 bp of exons 14 and 8, respectively. The next closest *Alu* to a deletion-prone cancer gene exon occurs at exon 19 of *FANCD2* with a separation of 20 bp. *ATM* and *CASP8* disproportionately increase in relative instability (compared to the other 48 genes in the deletion-prone cancer gene group) as the fraction of deletions ≤50 bp was increased (see Methods).

### Relative exon stabilities

The relative stabilities of the 1,287 coding exons that make up the 100 genes evaluated in this study were also compared. Figure S6A is a boxplot of the individual exon stabilities for the 50 deletion-prone cancer genes. Figure S6B is a similar boxplot for the 50 randomly selected genes. The two figures are constructed left-to-right based upon each gene's most unstable exon. These two figures illustrate that relative exon stability values tend to cluster in a gene specific manner. Within the deletion-prone cancer gene group the two left-most genes, *ATM* and *CASP8*, have moderate mean exon stability values. However, the presence of exons with outlying high instabilities within *ATM* and *CASP8* puts these two genes first and second place of the most unstable among the deletion-prone cancer genes. These two genes have *Alu* elements that are within 5 and 7 bp of their 14^th^ and 8^th^ exons, respectively. When average exon instability is used as the sorting criterion (illustrated by the bold black line through each respective boxplot), *VHL*, *BRCA1*, FANCA, *TP53* and *SBDS* make up the top 10% most unstable genes among the 50 deletion-prone cancer genes. Finally, Figure S6B illustrates the very low stability value (7.2) determined for the exon containing the putative exonized *Alu* in *GDPD2*.

### Deletion sizes in VHL cancer deletion families do not recapitulate Figure 5

Figure 5 is constructed upon the premise that over 95% of deletions in the human genome are less than 50 bp in length [26], [27]. In contrast, 25% of the deletions resulting in VHL cancer are greater than 10,000 bp [30]. This apparent conflict in deletion size frequency may arise from ascertainment bias as only those deletions that result in VHL cancer are detected. The *Alu* landscape flanking the VHL gene in Figure 4B reveals two regions of high *Alu* instability (*i*Scores shaped as horns) that extend in both 5′ and 3′ directions from the base of the *VHL* gene. As can be seen from the diagram, the 5′ and 3′ regions extend approximately 150,000 bp and 100,000 bp, respectively from the gene. Based on genome-wide derived deletion size frequencies in Figure 5, most of the deletions arising within these “horns of *Alu* instability” would be much shorter than the distances required to damage the VHL coding integrity and would likely go undetected.

## Discussion

Evolution is a slow process. The clues to its activity reside almost exclusively in the subtle patterns that it leaves behind. Two of these patterns, chimeric *Alus* and the instability of cancer genomes are consistent with this study's model of inverted *Alu* pair instability. The implications of these two evolutionary patterns are discussed below.

### Chimeric *Alus* may camouflage the instability of inverted *Alu* pairs

It is generally accepted that most chimeric *Alu* elements are formed by non-allelic homologous recombination (NAHR) between two direct oriented *Alu* elements [5]. However, chimeric *Alu* elements can also be generated by single-strand annealing repair of DSBs that occur within the spacer sequence separating a direct oriented *Alu* pair. However, single-strand annealing repair is only possible when high-homology sequences flank the DSB. Satisfying this homology requirement entails sufficient resection of the intervening spacer sequence separating the *Alu* pair [8].

The presence of a chimeric *Alu* element at the boundary, or breakpoint, of structural variation provides little evidence regarding the etiology of its formation. As a result, the mechanistic details behind this type of structural variation are difficult to ascertain. Without supporting evidence for an intervening deletion mechanism in the pre-chimeric spacer, the putative NAHR route is the most reasonable explanation for the formation of chimeric *Alu* elements.

This study's *Alu* structure-based stability algorithm was constructed upon the premise that DSBs can be generated from the interaction between inverted *Alu* pairs. It is possible that a fraction of these inverted *Alu* pair generated DSBs could be repaired through single-strand annealing repair of direct-oriented *Alu* pairs. This type of repair would generate a chimeric *Alu* element. The chimeric *Alu* element would effectively mask the inverted *Alu* pair as the source of the DSB. Further adding to this camouflage is the possibility that the chimeric *Alu* breakpoint (repair point) can be thousands of base pair removed from the initiating DSB [5], [31].

Both non-allelic homologous recombination and single strand annealing repair likely contribute to the human chimeric *Alu* population. However, to our knowledge, the strongest evidence in support of either theory is the imbalance in the human *Alu* pair I∶D ratio [18], [20]. Chimeric *Alu* elements appear to result from repair of approximately 10 percent of inverted APE deletions [18].

### Oncogenesis may be a passenger mutation to genome-wide instability

As mentioned previously in the Results section, the *Alu* element-based instability model predicts that deletion-prone cancer genes are ∼58% more unstable than randomly selected genes. This 58% difference between cancer and random gene deletion rates is not sufficiently large to preclude the possibility that both rates may be common products of an insidious process that damages the genomes of somatic cells. Prior to senescence, the trillions of cells in our bodies likely provide multiple occasions for an unfortunate combination of cancer-prone genetic damage to occur [32].

Most of the mutations in a cancer cell are passenger mutations that do not appear to contribute to the cancer cell's fitness [33]. It is generally assumed that the vast majority of these passenger mutations are byproducts of oncogenesis. While passenger mutations may be more likely to occur subsequent to the oncogenic driver mutation, the assumption that somatic cell genomes are stable prior to oncogenesis has not been proven.

In final support of a model suggesting general somatic cell instability is the observation that deletion size frequencies observed in VHL cancer (see Results) do not conform to the deletion size frequency distribution that has been observed in healthy cells (Figure 5). The disproportionate number of large deletions (relative to Figure 5) observed among various VHL cancer families suggests that many smaller, non-cancerous deletions occur, but go undetected within healthy cell populations.

### The human *Alu* pair I∶D ratio may underrepresent inverted *Alu* pair interactions

As previously stated, a premise of this study is that the imbalance in the human *Alu* pair I∶D ratio is a consequence of genomic instability. The human *Alu* pair I∶D imbalances illustrated in Figures 3 and S2 may under estimate inverted *Alu* pair instability for two reasons. 1) The depression of the I∶D ratio does not include inverted *Alu* pair deletions that have been lost through negative selection pressure and genetic drift. 2) The instability estimates derived from the I∶D ratio assumes no instability between direct oriented pairs. Several studies have shown that both inter-chromosomal and intra-chromosomal recombination occurs between *Alu* elements [5], [31], [34].

The development of this genomic instability model is just one approach to finding tangible risk factors associated with mobile element-related threats to the genome. Unfortunately, we are far from a complete understanding of the entire puzzle. However, the fundamentals provided by the algorithms used in this study may lay the foundation for other computational approaches to comparing genetic risks posed by structural variations that are unique to specific individuals, families and people groups. With the advancement of genome sequencing technologies and the emergence of whole genome analyses, sophisticated modeling systems such as this *Alu*-element based instability model, will likely be essential to the future of genomics research.

## Conclusions

Interactions between highly homologous *Alu* elements and their potential to result in deletions, duplications, inversions and gene conversion events has been well documented [5], [10], [14], [15], [35]. Various forms of structural variation have been shown to account for a large proportion of human genetic diversity [9], [26], [36]. Recent studies have suggested that common types of *Alu* induced structural variation may be just the tip of the iceberg, with far more complex mechanisms for *Alu* induced genome instability being possible [9], . The model developed in this study estimates relative human genome instability based upon the premise that inverted *Alu* pair exclusions are generated as a consequence of genomic instability.

Assuming that the basic concepts for this *Alu* element-based gene stability model are correct; five conclusions are evident from this study. 1) *Alu* landscapes create regions of genomic instability that are unique for each human gene. The majority of this instability resides within the ±250,000 bp regions flanking each gene. 2) Genes with higher exon counts are potentially more vulnerable to coding deletions. Additional exons provide more opportunities for *Alu* elements to reside in close proximity to coding regions. 3) Exonized *Alu* elements are a particularly unstable class of structural variation. This instability is inherent in exonized *Alus* because any deletion resulting from an *Alu-Alu* interaction is more likely to result in loss of coding sequence. 4) The human deletion size frequency curve predicts that large deletions detected through a cancer phenotype may be evidence that many smaller deletions also occur at the same locus, but go undetected. 5) This *Alu*-based human genome instability model may be used to evaluate the genetic risk posed by *Alu* element-based variation which is unique to specific individuals, families, and people groups.

## Methods

### Data acquisition and flow

The hg19, 2009 Human Genome Assembly was used for this study. Retrotransposon data was obtained from RepeatMasker [37] and downloaded from the UCSC genome BLAT Table Browser (UCSC Table Browser website. Available: http://genome.ucsc.edu/cgi-bin/hgTables?command=start. Accessed 2013 May 2). This data was imported into Excel 2010 (Microsoft Corporation; Redmond, Washington). Statistics were calculated using Excel 2010 output using Minitab 15 and Minitab 16 (Minitab Inc.; State College, Pennsylvania).

### Identification of the key variables that correlate with the human *Alu* pair I∶D ratio

Three variables were found to significantly correlate with the *Alu* pair I∶D ratio. These three variables are 1) the spacer size separating the two members of the *Alu* pair, 2) the number of *Alu* elements within the spacer separating the two members of the *Alu* pair and 3) the clustering state (clustered or not clustered) of the each member of the *Alu* pair.

The *Alu* pair I∶D ratio was not found to correlate strongly with *Alu* size. The only exception to this observation occurs between the first 10 immediate *Alu* neighbors of small-small and small-medium *Alu* pairs. Small *Alus* are between 30 and 135 bp in length and medium *Alus* are between 136 and 274 bp in length. This anomaly involves less than 0.2 percent of the *Alu* pair population. Manual inspection of several of these loci suggests that this phenomenon results from these smaller *Alu* fragments being incorporated into tandem repeats (data not shown). Incorporation of *Alu* fragments into tandem repeats lowers the I∶D ratio for pairs of this size.

### Description of key variables – spacer size

The spacer is the intervening sequence between the two *Alu* elements that make up an *Alu* pair. Spacer size is the number of base pairs within this intervening sequence. Additional *Alu* elements may be present within the spacer sequence.

### Description of key variables – *Alu* pair sequence number (APSN)

The parameter describing the number of *Alu* elements within the spacer of an *Alu* pair is termed the *Alu* pair sequence number (APSN). The APSN would ideally be defined as the number of *Alu* elements within the spacer sequence. However, the APSN uses either a positive or negative value to discriminate between pairs formed by *Alus* located either 5′ (negative) or 3′ (positive) of each *Alu* being evaluated. As a result, mathematical confounding of 5′ and 3′ adjacent pairs precludes the use of zero to describe this parameter. The APSN is consequently defined as the “n+1” number of *Alu* elements within the spacer.

### Description of key variables – clustering

The human non-LTR retrotransposons, *Alu* LINE and SVA elements, frequently cluster together in groups we previously defined as CLIQUEs, catenated *L*INE1 endonuclease induced *q*ueues of uninterrupted *Alu*, LINE1 and SVA elements [18]. Building on our original work, this study found that the *Alu* pair I∶D ratio is a strong function of the clustering state of *Alu* pairs (Figure S3). Four types of clustered *Alu* pairs exist and are identified as types 0, 1, 2 and 3. Type 0 and Type 1 *Alu* pairs are located within CLIQUEs. Type 0 *Alu* pairs are formed when both members of the pair reside within the same CLIQUE and Type 1 *Alu* pairs are formed when both members of the pair reside within different CLIQUEs. Type 0 pairs are rare (<0.5 percent of human *Alu* pair population) and because of the inherent orientational *Alu* biases within a CLIQUE, require a different methodology than I∶D ratio to determine instability [18]. This methodology is discussed separately under the heading entitled, “Determination of *Alu* pair instability within CLIQUEs”, below. Type 2 *Alu* pairs are hemi-clustered. This category of *Alu* pairs occurs where only one of the two *Alus* making up the pair resides within a CLIQUE. Type 3 *Alu* pairs are non-clustered. Figures 3 and S2 illustrate the relationship of I∶D ratio among different clustering conformations within the human *Alu* pair population.

### Algorithm development for estimating *Alu* pair I∶D ratio from key variables

Segregation of the separate contributions of spacer size, APSN and clustering to the *Alu* pair I∶D ratio was accomplished using a five-step methodology.

**Step one** in *Alu* pair I∶D ratio algorithm development was determination of the full-size *Alu* pair population (275–325 bp) with its associated I∶D ratio for each APSN (from APSN = ±1 through APSN = ±110). This information is available from previously published work (for APSNs 1–107) that utilized the human genome assembly hg18 as its resource [18]. This study updated the earlier work using improved techniques and the most recent human genome assembly, hg19. The improved techniques permitted extending the number of statistically significant APSNs from 107 to 110.

**Step two** in I∶D ratio algorithm development was accomplished by incrementally stepping through each of the populations of APSNs 1–110 in small (0.03–0.05% of each APSN population) spacer size increments. The population of *Alu* pair types 1, 2 and 3 (clustered, hemi-clustered and non-clustered) are determined within each increment. The resultant data set for each APSN and *Alu* pair type was then sorted into ten percentile groups. The first percentile accounts for the smallest five percent of the spacer sizes and the remaining nine percentiles capture sequential groupings of approximately ten percent of the APSN's *Alu* pair population. Each of these final nine percentiles is identified by its respective median point; 10^th^, 20^th^, 30^th^ etc., through 90^th^ percentiles. The spacer size boundaries for these final nine percentile groupings include ±5% of the *Alu* pair population for the APSN being evaluated. As examples, the 10^th^ percentile describes the grouping that includes spacer sizes ranging between the 5^th^ and 15^th^ percentiles, the 20^th^ percentile describes the spacer sizes falling between the 15^th^ and 25^th^ percentiles, etc. The *Alu* pair sample size for most APSN populations falls between 550,000 and 560,000. The only exceptions are the APSNs 1–4. These APSN families increase in population size from 461,054 to 548,606 because of CLIQUE (clustering) effects. An *Alu* pair population size above 507,000 is required to provide statistical confidence that an I∶D value ≤0.995 is below unity (p<0.05).

The percentile groupings are further reduced in size by subdividing them into their respective *Alu* pair types. The median spacer sizes along with actual and fitted I∶D ratios for Type 1 *Alu* pairs are shown in Table S2. As shown in Table S5, sample sizes across these spacer size percentile groupings reduce the sample size to as low as 2,611 for the 0–5^th^ percentile grouping for Type 1 *Alu* pairs for APSN = 1. The average sample size for the larger percentiles (APSN>1) is 18,574. This sample size problem for measuring the I∶D ratio for individual APSNs within percentiles and *Alu* pair types is addressed in step three of this five-step methodology.

**Step three** in *Alu* I∶D ratio algorithm development plots each of the ten percentile groupings for APSNs 1 through 115 against its median spacer size. This approach increases the population size for each percentile grouping by approximately 115X and permits more accurate estimation of the actual I∶D ratio at each APSN (see Figure S7). The smallest of these 115X sample sizes is 693,930 for the 2.5^th^ percentile of Type 1 *Alu* pairs. This sample size is larger than the 507,000 minimum sample size (see step two, above) required for I∶D values of <0.995 to be statistically less than unity (p<0.05). Examination of these 115 groupings revealed that for APSNs >110, no percentile grouping dropped below the minimum statistically significant I∶D value of 0.995 (p≤0.05). Consequently, only APSNs of 1 through 110 were used in the construction of the instability model algorithm.

A total of 30 regression curves were generated; 10 for Type 1 *Alu* pairs (clustered; 13,364,142 total full-length pairs), 10 for Type 2 *Alu* pairs (hemi-clustered; 28,537,478 total full-length pairs) and 10 for Type 3 *Alu* pairs (non-clustered; 18,836,832 total full-length pairs). Each set of percentile data was then regressed versus its median spacer size (from step two). The resultant algorithm that describes the data for each respective percentile was then assembled. In several instances the best fit for the data was accomplished by using a composite of two or more regressions for one set of APSN percentile data. Examples of these curve fits are shown in Figures S7A–C for the 2.5^th^ percentile curves for Type 1, 2 and 3 *Alu* pairs.

**Step four** in development of the *Alu* I∶D ratio prediction algorithm was the extraction of the respective I∶D ratios for each of the ten regressed percentiles for each APSN for *Alu* pair types 1, 2 and 3. Each regressed I∶D ratio value was plotted for each APSN against its median spacer size. This step produces 345 different I∶D curves, 115 curves for each *Alu* pair type. As mentioned previously, only APSN curves 1–110 had at least one point along the spacer size percentiles with an I∶D ratio that was statistically below unity (0.995 = p<0.05). This technique excludes *Alu* pair type zero, which was treated separately (see heading, “Determination of *Alu* pair instability within CLIQUEs”, below). An example of regressed data extracted from this step for Type 1 *Alu* pairs for APSNs 1–10 is shown in Figure 2. Figures 3 and S2 show the complete set of regressed I∶D data (APSNs = ± 1–110) for Type 1, 2 and 3 *Alu* pairs.

**Step five** in development of the *Alu* pair instability algorithm development was the regression of the ten percentile data points derived from step four (above) for each of the 345 graphs. The shape of these curves often requires more than one regression equation to accurately portray these regressed values. In addition, median spacer size values below the 2.5^th^ percentile and above the 90^th^ percentile fall outside of the regressed region for these curves. Spacer sizes that are smaller than the median spacer size for the 2.5^th^ percentile are assigned the I∶D value of the 2.5^th^ percentile. Straight lines connect the 2.5^th^ percentile midpoints for the 5′ and 3′ curves for each APSN for each of the three *Alu* pair types shown in Figures 3 and S2. Spacer sizes that are larger than the median spacer size for the 90^th^ percentile are fit along a straight line from the I∶D value at the 90^th^ percentile to unity at the 99^th^ percentile. The equation types and associated coefficients for the ±110 APSN curves associated with Type 1 *Alu* pairs are provided in Table S6.

### Determination of *Alu* pair instability within CLIQUEs

Type 0 *Alu* pairs possess inherent *Alu* orientational insertion biases. This is reflected by the low CLIQUE I∶D ratio = 0.460. These biases preclude the direct estimation of *Alu* pair instability from I∶D measurements [18]. However, less than 0.5% of human *Alu* pairs reside within the same CLIQUE. Most of these Type 0 *Alu* pairs have spacer sizes of ≤50 bp [18]. Although these pairs represent a relatively small fraction of the total *Alu* pair population, their small spacer size may make a disproportionately large contribution to the total inverted *Alu* pair instability within the genome.

A solution to this stability prediction dilemma for Type 0 *Alu* pairs was resolved using data from previous work performed with a yeast experimental system. This system measured the instability of inverted *Alu* pairs when separated by 12, 20, 30 and 100 bp for homologies of 94% and 100% [19]. Typical human *Alu* pair homologies are 85% [20].

Fortunately, the median spacer size for adjacent Type 1 (clustered) *Alu* pairs in 0th–5th percentile range was 100 bp (Table S5). This data point, representing 2,611 *Alu* pairs (Table S5), is one of the four spacer sizes evaluated for determination of inverted *Alu* pair instability in the experimental yeast system. This data point was used to anchor the 85% *Alu* homology curve to the 94% and 100% homology curves used in the yeast experiments [19]. The resultant Type 0 *Alu* pair algorithm for estimating inverted *Alu* pairs with 85% homologies is as follows.
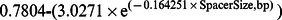
This algorithm is used to predict the I∶D ratio for Type 0 *Alu* spacer sizes ≤50 bp. The algorithms developed for Type 1 *Alu* pairs were used to estimate Type 0 *Alu* pairs with spacer sizes >50 bp.

### Instability estimate for individual *Alu* elements within an *Alu* pair

The I∶D ratio is the stability of an *Alu* pair, not the stability of an individual *Alu* element. The instability of an individual *Alu* element within an *Alu* pair is estimated as the square root of the I∶D ratio estimated for that pair. Depending upon the single-strand cleavage pattern at its eight potential cleavage sites (Figures 1 and S1; [18]), the resolution of the hypothetical doomsday junction can result in some level of gene conversion and/or from zero to four DSBs.

The I∶D ratio versus spacer size relationships represented in Figures 3 and S2 are composed of the ±110 APSNs curves for Type 1, 2 and 3 *Alu* pairs. Each of these APSN curves contain at least one percentile along their spacer size interval (Figure 3) where the I∶D ratio is <0.995. The I∶D<0.995 cutoff represents the statistical confidence interval for full-length *Alu* pair families (p<0.05). These curves permit the maximum inverted *Alu* pair interaction distance to be increased from the previously reported value of APSN = ±107 to APSN = ±110 (Cook et al. 2011). Any predicted I∶D ratio that is >0.995 is assigned a value of 1.0.

### *Alu* element stability and *i*Score determination

The stability of an *Alu* element is the grand product of the square root of the I∶D ratios calculated for each of the *Alu* pairs formed by its ±110 immediately flanking (5′ and 3′) *Alu* elements. This stability is expressed by the following equation.
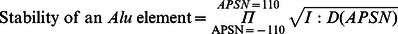
The stability of each of these 220 flanking *Alu* pairs is determined from the previously developed I∶D versus spacer size versus APSN algorithms. Direct oriented *Alu* pairs are considered stable and assigned a value of 1. The *i*Score is the inverse of the estimated stability of an *Alu* element and is used only in Figures 4 and S4 to illustrate the relative stabilities of the various *Alu* elements located within a gene's *Alu* landscape.

Since each end of an *Alu* element is subject to a potential deletion, the stability of only one end of each *Alu* element is the grand product of the fourth root of the I∶D ratio for all 220 potential *Alu-Alu* interactions. This stability is expressed as follows.
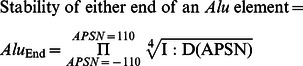
These algorithms are generated from the genome-wide human *Alu* element population. The individual *Alu* landscapes of the 100 genes examined in this study are of insufficient size to reveal statistically significant imbalances within their respective I∶D ratios. Using the average number of *Alus* within these landscapes (410 *Alus*), statistical relevance can only be recognized when the I∶D ratio is outside of the range of 0.82–1.22 (p<0.05).

### Estimation of deletion size probability

Two studies provided insight into the human deletion frequency distribution [26], [27]. Recent cancer studies also provide similar information. However, the unique nature of cancer cells precludes the use of this data in the characterization of DNA stability in healthy cells. In this study, human indel size frequency curves are treated as having the same shape as the corresponding human deletion size frequency curve.

The deletion size frequency curve in Figure 5 was prepared from a composite of data provided in the two studies mentioned, above. The first study, Wheeler et al., 2008, provides a deletion size frequency curve that was used to estimate the deletion size frequency for deletion sizes ≤75 bp. The second study [26], is used to estimate the deletion size frequency for deletion sizes >75 bp. Modeling of the deletion/indel size frequency data from both studies excluded the *Alu* insertion perturbation present between 250 and 350 bp. This permitted smoothing of the respective regression fits.

In the first study, deletion frequency data was regressed between 1 and 400 bp and for the second study, the indel frequency data was regressed between 50 and 10,000 bp. In both studies over 95% of deletions/indels were ≤50 bp. The second study (Mills et al., 2011) used a higher number of individuals (79) and thus supplied additional data for the more rare larger deletion sizes.

The sum of the 500,000 individual deletion size probabilities illustrated in Figure 5 equal 1.0. The probability of a specific deletion size occurring is lower than the probability of that same or larger deletion size occurring. This latter probability of a “minimum required deletion size or larger” required for loss of coding sequence is used in the model's algorithm.

The model's algorithm considers each end of each *Alu* element separately in its determination of exon and gene stability. Estimation of the risk that an *Alu* end poses to an exon coding sequence first requires that the distance between the end of the *Alu* element and the proximal end of the exon be determined. This distance is defined as D_Min_. The formula that describes the probability of a minimum deletion size is as follows.

D_Min_ = Probability of a specific deletion size (or larger) = P_Deletion_

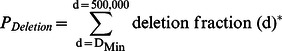
* Individual deletion fractions are taken from Figure 5

### Determination of relative exon instability

Individual exon instabilities are calculated through a five-step process. Step one is calculating the DSB risk posed by each end of each *Alu* element (Risk_End_) within a gene's ±250,000 bp *Alu* landscape. Step two is determining the potential deletion size risk, P_Deletion_, posed by each end of each *Alu* element within this landscape, to the coding exon of interest. Step three is multiplying each individual Risk_End_ value by its respective P_Deletion_ value. Step four calculates the grand product of these “Risk_End_×P_Deletion_” products. This estimated relative exon stability, Exon_RS_, is expressed by the following formula.
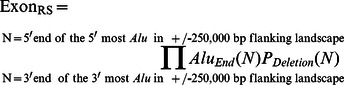
Step five determines the exon instability. Since exon stability plus exon instability equals 1.0, the exon instability is one minus the estimated exon stability derived from the formula above.

### Determination of relative gene instability

Relative gene instability is defined as the relative likelihood of a deletion occurring at some location within a gene's coding exons. This is determined through a four-step process. The first three steps are identical to the first three steps described under the “Determination of relative exon instability” heading above. Step three in this procedure is only performed for the closest exon to each *Alu* element end. This step determines the highest risk, Risk_Max_, that one end of an *Alu* element can pose to a gene. Step four multiplies each of these, Risk_Max_, values determined for each *Alu* end. This grand product produces the estimate of that gene's relative stability, Gene_RS_.
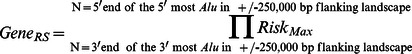
Step five determines the gene instability. Since the stability of a gene plus its instability equals 1.0, gene instability is one minus the estimated gene stability derived from the formula above.

### Gene selection

The 50 random human genes used in this study were selected from the list of 19,026 human protein-coding genes provided by the HUGO Gene Nomenclature Committee, HGNC. The source file containing these genes was downloaded from the HGNC website [38]. The 50 random genes were selected from this list using Minitab 16.

The 50 deletion-prone cancer genes were selected from [21], [23] and the Catalogue of Somatic Mutations in Cancer [22] (Wellcome Trust Sanger Institute Cancer Genome Project website. Available: http://www.sanger.ac.uk/genetics/CGP/Census/large_deletion.shtml. Accessed 2013 May 2). Only coding exons were selected for each gene. Exon loci were obtained from the RefSeq CDS Fasta Alignment page on the UCSC genome browser (UCSC Genome Browser website. Available: http://genome.ucsc.edu/cgi-bin/hgPal. Accessed 2013 May 2). Variant 1 isoforms of all genes were selected when more than one gene was listed under RefSeq genes.

### Variation in relative gene stability with variation in deletion size frequency

The deletion size frequency curve used in this study (Figure 5) illustrates that 95% of human deletions have lengths ≤50 bp. The availability of genome-wide human deletion size frequency data is limited. Consequently, the sensitivity of relative gene stabilities to the shape of this deletion frequency curve was examined. This examination was made by varying the fraction of deletions that were ≤50 bp from 0.90 to 0.99 in increments of 0.01 for the 50 deletion-prone cancer genes. The results of this examination showed that the relative stabilities of 48 of the 50 deletion-prone cancer genes remained essentially unchanged as the ≤50 bp increment was varied. Exceptions to this observation occurred with two genes, *ATM* and *CASP8*, which have the rare occurrence of *Alus* within 5 and 7 bp of their exons [28]. The results of this sensitivity analysis are shown in Figure S5.

## Supporting Information

Table S1 **Selected studies linking** ***Alu*** **-related deletions to disease phenotypes.**
(PDF)

Table S2 **Raw and fitted I∶D ratios for the ten spacer size groupings for Type 1** ***Alu*** **pairs.**
(PDF)

Table S3 **Characteristics of the 50 deletion-prone human cancer genes examined in this study.**
(PDF)

Table S4 **Characteristics of the 50 randomly chosen human genes examined in this study.**
(PDF)

Table S5 **Spacer size percentile samples sizes versus APSN for Type 1** ***Alu*** **pairs.**
(PDF)

Table S6 **Coefficients for equations describing the I∶D ratio versus spacer size for Type 1** ***Alu*** **pairs.**
(PDF)

Figure S1 **A proposed mechanism for the formation of a doomsday junction that is catalyzed by the ectopic invasion and annealing of complementary replication forks.** (A) Two *Alu* elements in opposite orientations form an inverted *Alu* pair. (B) Concomitant advancement of replication forks through each member of an inverted *Alu* pair. C) Bending of the DNA to permit alignment of the complementary replication forks. D) Ectopic invasion and annealing of single-stranded DNA associated between high-homology replication forks could potentially extend to the entire length of the *Alu* elements. The hypothetical conformation created by this interaction is termed a doomsday junction. As also illustrated in Figure 1, eight segments of single-stranded DNA are formed at the boundary of the doomsday junction and create the opportunity for single-strand nuclease attack. These sites are illustrated as yellow lightning bolts.
(PDF)

Figure S2 **The** ***Alu*** **pair I∶D ratio versus spacer size for Type 2 and Type 3** ***Alu*** **pairs for APSNs ±1–110.** This figure illustrates the ±110 APSN curves for full-length (275–325 bp) A) Type 2 *Alu* pairs and B) Type 3 human *Alu* pairs.
(PDF)

Figure S3 **I∶D ratios for Type 1, 2 and 3** ***Alu*** **pair families for APSNs 1–150.** Note that the departure of the I∶D ratio from unity is greatest for clustered (Type 1) *Alu* pairs and closest to unity for non-clustered (Type 3) *Alu* pairs.
(PDF)

Figure S4 ***Alu*** **landscapes for five deletion-prone cancer genes and three randomly chosen genes.** Each *Alu* element is plotted within and 500 kbp, 5′ and 3′ flanking each gene. The locus of each *Alu* is plotted against its respective instability score, *i*Score. The *i*Score is the inverse of the model's predicted *Alu* stability and thus larger values represent higher instabilities. The five selected deletion-prone cancer genes are A) *APC*, B) *ATM*, C) *MLH1*, D) *MSH2* and E) *TP53*. The three randomly chose genes are F) *GDPD2*, G) *KEAP1* and H) *SF3B3*.
(PDF)

Figure S5 **Sensitivity analysis of the relative stabilities of the deletion-prone cancer genes versus variation in the fraction of deletions that is ≤50 bp.** The shape of the deletion size frequency curve used to determine relative gene stabilities (Figure 5) places 95% of deletions with lengths of ≤50 bp. This figure examines the variation in relative deletion-prone cancer gene stabilities as the fraction of deletions ≤50 bp is varied between 90% and 99%. As can be seen from this figure, the relative stabilities of 48 of the 50 deletion-prone cancer genes (96%) remain essentially unchanged as the ≤50 bp increment is varied. Exceptions to this observation are observed with *ATM* and *CASP8* (bolded curves), which have the rare occurrence of *Alus* within 5 and 7 bp of their exons, respectively. These two genes exhibit higher relative stabilities as the fraction of deletions ≤50 bp in length increases.
(PDF)

Figure S6 **Estimated relative exon stability distributions for the 50 deletion-prone cancer genes and 50 randomly chosen genes.** A) Boxplot of the individual exon stabilities for the 50 deletion-prone cancer genes. The genes in this figure are ordered left-to-right on the basis of each gene's least stable exon. While individual exon stabilities vary widely, they tend to cluster in a gene specific manner. Exceptions to this pattern are illustrated by the presence of a single, outlying low stability exon within *ATM* and *CASP8*. These individual exon stabilities place these two genes at first and second place of highest instability among these 50 deletion-prone cancer genes. (B) Boxplot of the individual exon stabilities for the 50 randomly selected genes. Note that a broken Y-axis scale is required to capture the low stability of the putative exonized *Alu* in the 12^th^ exon of *GDPD2* (see text).
(PDF)

Figure S7 **Fitted curves for 2.5th percentile spacer size groupings for type 1, 2 and 3 for APSN families 1–115.** These three curves are part of thirty curves that are used to estimate the I∶D ratio for the Type 1, Type 2 and Type 3 *Alu* pairs. Ten different curves are used for each *Alu* Pair Type. These ten curves are used to construct an I∶D ratio curve for each APSN family versus spacer size (see Methods). The curves shown here for A) Type 1 B) Type 2 and C) Type 3 *Alu* pairs represent the I∶D ratio for the smallest median spacer size percentile (2.5^th^ percentile) of spacer size groupings.
(PDF)
