# Supplementary material for: A Comparison of 100 Human Genes Using an Alu Element-Based Instability Model
Source: PLoS One. 2013 Jun 3;8(6):e65188. doi: 10.1371/journal.pone.0065188 (PMC3670932; doi:10.1371/journal.pone.0065188)
Supplement: Table S1 — Selected studies linking Alu -related deletions to disease phenotypes. (PDF) [file pone.0065188.s001.pdf]

Studies Linking *Alu*-related Deletions to Disease Phenotypes

No.	First Author	Year	Locus	Phenotype[(1)]	Title	Journal[(2)]	Vol	Issue
1	F. Duraturo	2013	2p21	LS	Contribution of Large Genomic Rearrangements in Italian Lynch Syndrome Patients: Characterization of a Novel *Alu*-Mediated Deletion	BRI	'13	1
2	K. Kitada	2013	7q22.1	C	*Alu-Alu* Fusion Sequences Identified at Junction Sites of Copy Number Amplified Regions in Cancer Cell Lines	CGR	139	1
3	C. Vaughn	2013	7p22.1	LS	The Frequency of Previously Undetectable Deletions Involving 3' Exons of the *PMS2* Gene	GCC	52	1
4	M. Barbaro	2012	3q11.2	HCP	Identification of an *AluY* -mediated deletion of exon 5 in C*POX* gene by MLPA analysis in patients with hereditary coproporphyria	CG	81	3
5	N. Bondurand	2012	22q13.1	WS IV	*Alu*-mediated deletion of *SOX10* regulatory elements in Waardenburg syndrome type 4	EJHG	20	9
6	V. Chanavat	2012	11p11.2	HCM	Molecular characterization of a large *MYBPC3* rearrangement in a cohort of 100 Unrelated patients with hypertrophic cardiomyopathy	EJMG	55	3
7	M. Coutinho	2012	12q23.2	ML II	*Alu-Alu* Recombination Underlying the First Large Genomic Deletion in GlcNAc--Phosphotransferase Alpha/Beta (*GNPTAB*) Gene in a MLII Alpha/Beta Patient	JIMD	4	1
8	A. Eiden-Plach	2012	8p11.23	LCAH	*AluSx* repeat-induced homozygous deletion of the StAR gene causes lipoid congenital adrenal hyperplasia	JSBMB	130	1-2
9	A. Gonçalves	2012	17p13.1	LFS	Li-Fraumeni-like syndrome associated with a large BRCA1 intragenic deletion	BMCC	12	1
10	A. Jelassi	2012	19p13.2	ADH	Genomic characterization of two deletions in the *LDLR* gene in Tunisian patients with familial hypercholesterolemia	CCA	414	-
11	H. Mahmoudi	2012	13q14.2	HY	Identification of an *Alu*-mediated 12.2-kb deletion of the complete *LPAR6 (P2RY5)* gene in a Turkish family with hypotrichosis and wooly hair	ED	21	6
12	M. Pereira	2012	15q21.1	SPG 11	*Alu* elements mediate large *SPG11* gene rearrangements: further spatacsin mutations	GM	14	1
13	L. Pezzoli	2012	11p11.2	HCM	A new mutational mechanism for hypertrophic cardiomyopathy	GE	507	2
14	M. Vlckova	2012	6q	M	Mechanism and Genotype-Phenotype Correlation of Two Proximal 6q Deletions Characterized Using mBAND, FISH, Array CGH, and DNA Sequencing	CGR	136	1
15	T. Arai	2011	Xq22.1	XLA	Genetic analysis of contiguous X-chromosome deletion syndrome encompassing the *BTK* and *TIMM8A* genes	JHG	56	8

Studies Linking *Alu*-related Deletions to Disease Phenotypes

16	P. Boone	2011	2p22.3	SPG IV	*Alu*-specific microhomology-mediated deletion of the final exon of *SPAST* in three unrelated subjects with hereditary spastic paraplegia	GM	13	6
17	G. Borck	2011	6p24.3-2	CC	An *Alu* repeat-mediated genomic *GCNT2* deletion underlies congenital cataracts and adult i blood group	HG	131	2
18	M. Cozar	2011	1q22	GD	Molecular characterization of a new deletion of the GBA1 gene due to an inter *Alu* recombination event	MGM	102	2
19	I. Guella	2011	1q24.2	FVD	Identification of the first *Alu* -mediated large deletion involving the *F5* gene in a compound heterozygous patient with severe factor V deficiency	JTH	106	2
20	X. Guo	2011	22q11.2	DGS	Characterization of the past and current duplication activities in the human 22q11.2 region	BMCG	12	71
21	I. Jennes	2011	8q24.11	MO	Breakpoint characterization of large deletions in *EXT1* or *EXT2* in 10 Multiple Osteochondromas families	BMCMG	12	85
22	R. Kuiper	2011	2p21	LS	Recurrence and Variability of Germline *EPCAM* Deletions in Lynch Syndrome	HGVS	32	4
23	M. Kurnikova	2011	1q21.3	SCN	*Alu*-Mediated Recombination in the *HAX1* Gene as the Molecular Basis of Severe Congenital Neutropenia	AJMG	155A	3
24	M. Legarda	2011	16q22.2	T II	Large *TAT* deletion in tyrosinaemia type II patient	MGM	104	3
25	J. Oshima	2011	Xq28	MPS II	LCR-initiated rearrangements at the *IDS* locus, completed with *Alu*-mediated recombination or non-homologous end joining	JHG	56	7
26	L. Perez-Cabornero	2011	2p21	LS	Characterization of New Founder *Alu*-Mediated Rearrangements in *MSH2* Gene Associated with a Lynch Syndrome Phenotype	CPR	4	10
27	H. Raef	2011	11q13.1	MEN I	A novel deletion of the *MEN1* gene in a large family of multiple endocrine neoplasia type 1 (*MEN1*) with aggressive phenotype	CE	75	6
28	A. Rose	2011	19q13.42	RP	A 112kb deletion in chromosome 19q13.42 leads to retinitis pigmentosa	IOVS	52	9
29	M. Sluiter	2011	17q21.31	BC	Large genomic rearrangements of the *BRCA1 and BRCA2* genes: review of the Literature and report of a novel *BRCA1* mutation	BCRT	125	2
30	M. Soejima/Y. Koda	2011	19q13.33	BP	TaqMan-based real-time polymerase chain reaction for detection of FUT2 copy number Variations: identification of novel *Alu* -mediated deletion	T	51	4
31	J. Wan	2011	19p13.2	EA II	Large genomic deletions in *CACNA1A* cause episodic ataxia type 2	FN	2	-
32	K. Champion	2010	17q21.2	SS B	Identification and characterization of a novel homozygous deletion in the α-N-acetyl--glucosaminidase gene in a patient with Sanfilippo type B syndrome	MGM	100	1

Studies Linking *Alu*-related Deletions to Disease Phenotypes

33	M. DeRosa	2010	19p13.3	PJS	*Alu* -Mediated Genomic Deletion of the Serine/Threonine Protein Kinase 11 (*STK11)* Gene in Peutz-Jeghers Syndrome	G	138	7
34	M. Gentsch	2010	1q25.3	CGD	Alu-Repeat--Induced Deletions Within the *NCF2* Gene Causing p67-*phoxi*-Deficient Chronic Granulomatous Disease (CGD)	HGVS	31	2
35	A. Janecke	2010	11q23.1	PGL	Identification of a 4.9-kilo base-pair *Alu*-mediated founder *SDHD* deletion in two extended paraganglioma families from Austria	JHG	55	3
36	M. Kleppe	2010	18p11.21	T-ALL	Deletion of protein tyrosine phosphatase gene *PTPN2* in T-cell acute lymphoblastic leukemia	NG	42	6
37	A. Lindstrand	2010	10p14	HDR	Molecular and Clinical Characterization of Patients with Overlapping 10p Deletions	AJMG	152A	5
38	M. McCabe	2010	19p13.3	PJS	Homozygous Deletion of the *STK11/LKB1* Locus and the Generation of Novel Fusion Transcripts in Cervical Cancer Cells	CGC	197	2
39	M. Phylipsen	2010	16p13.3	αT	A new α^0-thalassemia deletion found in a Dutch family (- ^{AW})	BCMD	45	2
40	V. Picard	2010	1q25.1	AT I	Detection and characterization of large *SERPINC1* deletions in type I inherited antithrombin deficiency	HG	127	1
41	N. Resta	2010	19p13.3	PJS	Breakpoint determination of 15 large deletions in Peutz-Jeghers subjects	HG	128	4
42	Z. Yang	2010	Xq24	DD	LAMP2 Microdeletions in Patients with Danon Disease	CCG	3	2
43	F. Zhang	2010	17p12	N	Mechanisms for Nonrecurrent Genomic Rearrangements Associated with CMT1A or HNPP: Rare CNVs as a Cause for Missing Heritability	AJHG	86	6
44	L. Desviat	2009	13q32.3	PA	High frequency of large genomic deletions in the PCCA gene causing propionic acidemia	MGM	96	4
45	A. Erez	2009	Xp22.13	RTT	*Alu*-specific microhomology-mediated deletions in *CDKL5* in females with early-onset seizure disorder	N	10	4
46	G. Franke	2009	3p25.3	VHL	*Alu-Alu* Recombination Underlies the Vast Majority of Large *VHL* Germline Deletions: Molecular Characterization and Genotype--Phenotype Correlations in VHL Patients	HM	30	5
47	C. Oliveria	2009	16q22.1	HDGC	Germline *CDH1* deletions in hereditary diffuse gastric cancer families	HMG	18	9
48	A. Pangrazio	2009	11q13.2	ARO	Characterization of a Novel *Alu-Alu* Recombination-Mediated Genomic Deletion in the *TCIRG1* Gene in Five Osteopetrotic Patients	JBMR	24	1
49	R. Quental	2009	Xp11.4	OTCD	Molecular mechanisms underlying large genomic deletions in ornithine transcarbamylase (*OTC*) gene	CG	75	5

Studies Linking *Alu*-related Deletions to Disease Phenotypes

50	H. Singh	2009	Xq28	BS	A Novel *Alu*-Mediated Xq28 Microdeletion Ablates *TAZ* and Partially Deletes *DNL1L* in a Patient with Barth Syndrome	AJMG	149A	5
51	A. Mohl	2008	12p13.31	VWD	An *Alu* -mediated novel large deletion is the most frequent cause of type 3 von Willebrand disease in Hungary	JTH	6	10
52	S. Quental	2008	19q13.2	MSUD	Maple syrup urine disease due to a new large deletion at *BCKDHA* caused by non-homologous recombination	JIMD	31	2
53	M. Zikan	2008	17q21.31	BC	Novel complex genomic rearrangement of the *BRCA1* gene	MR	637	1-2
54	S. Armaou	2007	17q21.31	BC	Novel genomic rearrangements in the *BRCA1* gene detected in greek breast/ovarian cancer patients	EJC	43	2
55	E. Costa	2007	7q11.21	SDS	Identification of a novel *AluSx*-mediated deletion of exon 3 in the SBDS gene in a patient with Shwachman-Diamond syndrome	BCMD	39	1
56	T. Fukao	2007	Xp22.13	XLG	Identification of *Alu*-mediated, large deletion-spanning introns 19-26 in *PHKA2* in a patient with X-linked liver glycogenosis (hepatic phosphorylase kinase deficiency)	MGM	92	1-2
57	B. Hayward	2007	2p22.3	L	Extensive Gene Conversion at the *PMS2* DNA Mismatch Repair Locus	HM	28	5
58	M. Okubo	2007	8p21.3	LPL	A novel complex deletion--insertion mutation mediated by *Alu* repetitive elements leads to lipoprotein lipase deficiency	MGM	92	3
59	M. Smyk	2007	Xp21.2	AHC	Male-to-female sex reversal associated with ~250 kb deletion upstream of *NR0B1 (DAX1)*	HG	122	1
60	E. Di Pierro	2006	11q23.3	AIP	A large deletion on chromosome 11 in acute intermittent porphyria	BCMD	37	1
61	A. Fukuuchi	2006	11q13.1	MEN I	A Whole *MEN1* Gene Deletion Flanked by *Alu* Repeats in a Family with Multiple Endocrine Neoplasia Type 1	JJCO	36	11
62	C. Has	2006	20p12.3	KS	Molecular Basis of Kindler Syndrome in Italy: Novel and Recurrent *Alu/Alu* Recombination, Splice Site, Nonsense, and Frameshift Mutations in the *KIND1* Gene	JID	126	8
63	G. Humbert	2006	15q26.1	RPA	Homozygous Deletion Related to Alu Repeats in RLBP1 Causes Retinitis Punctata Albescens	IOVS	47	11
64	V. Matejas	2006	17p12	HNPP	Identification of *Alu* elements mediating a partial *PMP22* deletion	N	7	2
65	S. Preisler-Adams	2006	17q21.31	BC	Gross rearrangements in *BRCA1* but not *BRCA2* play a notable role in predisposition to breast and ovarian cancer in high-risk families of German origin	CGC	168	1
66	F. Xie	2006	12p13.31	VWD	A novel *Alu*-mediated 61-kb deletion of the von Willebrand factor (*VWF*) gene whose breakpoints co-locate with putative matrix attachment regions	BCMD	36	3

Studies Linking *Alu*-related Deletions to Disease Phenotypes

67	G. Zhang	2006	6q27	T2D	Identification of *Alu*-mediated, large deletion-spanning exons 2-4 in a patient with mitochondrial acetoacetyl-CoA thiolase deficiency	MGM	89	3
68	S. Agata	2005	13q13.1	BC	Large genomic deletions inactivate the *BRCA2* gene in breast cancer families	JMG	42	10
69	C. Bergmann	2005	6p12.3-2	ARPKD	Multi-exon deletions of the *PKHD1* gene cause autosomal recessive polycystic kidney disease (ARPKD)	JMG	42	10
70	F. Charbonnier	2005	2p21	HNPCC	The 5' Region of the *MSH2* Gene Involved in Hereditary Non-Polyposis Colorectal Cancer Contains a High Density of Recombinogenic Sequences	HM	26	3
71	F. del Castillo	2005	13q12.11	ARNSHI	A novel deletion involving the connexin-30 gene, del(*GJB6-d13s1854*), found in *trans* with mutations in the *GJB2* gene (connexin-26) in subjects with DFNB1 non-syndromic hearing impairment	JMG	42	7
72	C. Dobson-Stone	2005	9q21.2	ChAc	Identification of a *VPS13A* founder mutation in French Canadian families with chorea-acanthocytosis	N	6	3
73	J. Douglas	2005	5q35.2-3	SS	Partial *NSD1* deletions cause 5% of Sotos syndrome and are readily identifiable by multiplex ligation dependent probe amplification	JMG	42	9
74	C. Eng	2005	Xq22.1	FD	Molecular Basis of Fabry Disease: Mutations and Polymorphisms in the Human α-Galactosidase A Gene	HM	3	2
75	C. Giunta	2005	1p36.22	EDS	Mutation analysis of the *PLOD1* gene: An efficient multistep approach to the molecular diagnosis of the kyphoscoliotic type of Ehlers-Danlos syndrome (EDS VIA)	MGM	86	1-2
76	S. Hsieh	2005	1p36	HCC	High-freq. *Alu*-mediated recomb./del. within the *hCAD* in hepatoma	O	24	43
77	H. van der Klift	2005	2p21	HNPCC	Molecular Characterization of the Spectrum of Genomic Deletions in the Mismatch Repair Genes *MSH2, MLH1, MSH6,* and *PMS2* Responsible for HNPCC(1)	GCC	44	2
78	B. Baysal	2004	11q23.1	PGL	An *Alu*-mediated partial *SDHC* deletion causes familial and sporadic paraganglioma	JMG	41	9
79	U. Guenther	2004	11q13.3	SMARD1	Genomic rearrangements at the *IGHMBP2* gene locus in two patients with SMARD1	HG	115	4
80	C. Hartmann	2004	17q21.31	BC	Large *BRCA1* Gene Deletions Are Found in 3% of German High-risk Breast Cancer Families	HM	24	6
81	F. Laccone	2004	Xq28	RS	Large Deletions of the MECP2 Gene Detected by Gene Dosage Analysis in Patients With Rett Syndrome	HM	23	3
82	M. Mitchell	2004	4q35.2	FXID	An *Alu*-mediated 31.5-kb deletion as the cause of factor XI deficiency in 2 unrelated patients	B	104	8
83	S. Nakaya	2004	Xq28	HA	Severe HA(1) due to a 1.3 kb factor VIII gene deletion including exon 24: homologous recombination between 41 bp within an Alu repeat sequence in introns 23 and 24	JTH	2	11

Studies Linking *Alu*-related Deletions to Disease Phenotypes

84	L. Rossetti	2004	Xq28	HA	Homologous Recombination Between AluSx-Sequences as a Cause of Hemophilia	HM	24	5
85	C. Silao	2004	1p21.2	MSUD	A novel deletion creating a new terminal exon of the dihydrolipoyl transacylase gene is a founder mutation of Filipino maple syrup urine disease	MGM	81	2
86	I. Tournier	2004	13q13.1	BC	Significant Contribution of Germline *BRCA2* Rearrangements in Male Breast Cancer Familes	CR	64	22
87	M. Venturin	2004	17q11.2	NF1	Evidence for non-homologous end joining and non-allelic homologous recombination in atypical *NF1* microdeletions	HG	115	1
88	C. Bergmann	2003	Xq12	XMR	Oligophrenin 1 (*OPHN1*) gene mutation causes syndromic X-linked mental retardation with epilepsy, rostral ventricular enlargement and cerebellar hypoplasia	BJN	126	7
89	E. Jo	2003	Xq22.1	XLA	Identification of mutations in the Bruton’s tyrosine kinase gene, including a novel genomic rearrangements resulting in large deletion, in Korean XLA[(1)] patients	JHG	48	6
90	V. Ricci	2003	Xq28	HD	An *Alu*-mediated rearrangement as cause of exon skipping in Hunter disease	HG	112	4
91	R. Shaji	2003	16p13.3	HbH	Determination of the breakpoint and molecular diagnosis of a common α-thalassaemia-1 deletion in the Indian population	BJH	123	5
92	Y. Wang	2003	2p21	HNPCC	Hereditary Nonpolyposis Colorectal Cancer: Frequent Occurrence of Large Genomic Deletions in *MSH2* and *MLH1* Genes	IJC	103	5
93	W. Balemans	2002	17q21.31	VBD	Identification of a 52 kb deletion downstream of the *SOST* gene in patients with van Buchem disease	JMG	39	2
94	Z. Guo	2002	9q31.1	TD	Double deletions and missense mutations in the first nucleotide-binding fold of the ATP-binding cassette transporter A1 (*ABCA1*) gene in Japanese patients with TD[(1)]	JHG	47	6
95	M. Huber	2002	10q24-25	EB	Deletion of the Cytoplasmatic Domain of BP180/Collagen XVII Causes a Phenotype with Predominant Features of Epidermolysis Bullosa Simplex	JID	118	1
96	M. Lutskiy	2002	Xp11.23	WAS	An *Alu*-mediated *deletion* at Xp11.23 leading to Wiskott-Aldrich syndrome	HG	110	5
97	K. Staehling-Hampton	2002	17q12-q21	VBD	A 52-kb Deletion in the *SOST-MEOX1* Intergenic Region on 17q12-q21 Is Associated With van Buchem Disease in the Dutch Population	AJMG	110	2
98	F. Vidal	2002	Xq28	HA	First Molecular Characterization of an Unequal Homologous *Alu*-mediated Recombination Event Responsible for Hemophilia	JTH	88	1
99	T. Yabe	2002	6p21.32	BLS	A subject with a novel type I bare lymphocyte syndrome has tapasin deficiency due to deletion of 4 exons by *Alu*-mediated recombination	B	100	4
100	X. Cao	2001	5q22.2	FAP	Topoisomerase-I- and *Alu*-mediated genomic deletions of the *APC* gene in familial adenomatous polyposis	HG	108	5

Studies Linking *Alu*-related Deletions to Disease Phenotypes

101 F. Ringpfeil	2001	16p13.11	PE	Compound Heterozygosity for a Recurrent 16.5-kb *Alu*-Mediated Deletion Mutation and Single-Base-Pair Substitutions in the *ABCC6* Gene Results in PE[(1)]	AJHG	68	3
102 T. Wang	2001	13q13.1	BC	A Deletion/Insertion Mutation in the *BRCA2* Gene in a Breast Cancer Family: A Possible Role of the *Alu*-polyA Tail in the Evolution of the Deletion	GCC	31	1
103 S. Dabora	2000	16p13.3	TSC	Characterisation of six large deletions in *TSC2* identified using long range PCR suggests diverse mechanisms including Alu mediated recombination	JMG	37	11
104 M. Hiltunen	2000	14q24.2	EOAD	Identification of novel 4.6-kb genomic deletion in presenilin-1 gene which results in exclusion of exon 9 in a Finnish early onset Alzheimer's disease family: an *Alu* core sequence-stimulated recombination?	EJHG	8	4
105 Y. Koda	2000	19q13.33	BP	An *Alu*-mediated large deletion of the *FUT2* gene in individuals with the ABO-Bombay phenotype	HG	106	1
106 E. Rohlfs	2000	17q21.31	BC	An *Alu*-Mediated 7.1 kb Deletion of *BRCA1* Exons 8 and 9 in Breast and Ovarian Cancer Families That Results in Alternative Splicing of Exon 10	GCC	28	3
107 Y. Saikawa	2000	22q12.3	HO-1	Structural Evidence of Genomic Exon-Deletion Mediated by *Alu-Alu* Recombination in a Human Case with Heme Oxygenase-1 Deficiency	HM	16	2
108 R. Suminaga	2000	Xp21.1-2	DMD	Non-homologous recombination between *Alu* and LINE-1 repeats caused a 430-kb deletion in the dystrophin gene: a novel source of genomic instability	JHG	45	6

---

(1)

<u>Phenotype Abbreviations</u>

ADH - Autosomal Dominant Hypercholesterolemia
AHC - Congenital Adrenal Hypoplasia
AIP - Acute Intermittent Porphyria
ARNSHI - Autosomal Recessive Non-Syndromic Hearing Impairment
ARO - Autosomal Recessive Osteopetrosis
ARPKD - Autosomal Recessive Polycystic Kidney Disease
AT I - Antithrombin Deficiency Type I
BC - Breast Cancer

Studies Linking *Alu*-related Deletions to Disease Phenotypes

BLS -	Type I Bare Lymphocyte Syndrome
BP -	Bombay Phenotype
BS -	Barth Syndrome
C -	Cancer
CC -	Congenital Cataracts
CGD -	Chronic Granulomatous Disease
ChAc -	Chorea-acanthocytosis
DD -	Danon Disease
DGS -	DiGeorge Syndrome (in paper #20, LCR22's are related to 3 other phenotypes)
DMD -	Duchenne Muscular Dystrophy
EA II -	Episodic Ataxia Type 2
EB -	Epidermolysis Bullosa Simplex
EDS -	Ehlers-Danlos Syndrome
EOAD -	Early Onset Alzheimer's Disease
FAP -	Familial Adenomatous Polyposis
FD -	Fabry Disease
FVD -	Factor V Deficiency
FXID -	Factor XI Deficiency
GD -	Gaucher Disease
HA -	Hemophilia A
HbH -	Haemoglobin H Disease
HCC -	Hepatocellular Carcinoma
HCM -	Hypertrophic Cardiomyopathy
HCP -	Hereditary Coproporphyria (other phenotypes mentioned in paper 34)
HD -	Hunter Disease
HDGC -	Hereditary Diffuse Gastric Cancer
HDR -	HDR Sydrome (Hypoparathyroidism, Sensorineural Deafness, Renal Dysplasia)
HNPCC -	Hereditary Non-Polyposis Colorectal Cancer
HNPP -	Hereditary Neuropathy with Liability to Pressure Palsies
HY -	Hypotrichosis
KS -	Kindler Syndrome
L -	Leukemia
LCAH -	Lipoid Congenital Adrenal Hyperplasia
LFS -	Li-Fraumeni Syndrome
LPL -	Lipoprotein Lipase Deficiency

Studies Linking *Alu*-related Deletions to Disease Phenotypes

LS -	Lynch Syndrome
M -	Microcephaly (in paper #14, other phenotypes related to 6q deletions are mentioned)
MEN I -	Multiple Endocrine Neoplasia Type I
ML II -	Mucolipidosis Type II α/β
MO -	Multiple Osteochondromas
MPS II -	Mucopolysaccharidosis Type II
MSUD -	Maple Syrup Urine Disease
N -	Neuropathy
NF1 -	Neurofibromatosis Type I
OTCD -	Ornithine Transcarbamylase Deficiency
PA -	Propionic Acidemia
PE -	Pseudoxanthoma Elasticum
PGL -	Paraganglioma
PJS -	Peutz-Jeghers Syndrome
RP -	Retinitis Pigmentosa
RPA -	Retinitis Punctata Albescens
RS -	Rett Syndrome
RTT -	Rett Syndrome
SCN -	Severe Congenital Neutropenia
SDS -	Shwachman-Diamond Syndrome
SMARD1 -	Spinal Muscular atrophy with Respiratory Distress Type I
SPG 11 -	Spastic Paraplegia Type 11
SPG IV -	Spastic Paraplegia Type IV
SS -	Sotos Syndrome
SS B -	Sanfilippo Syndrome Type B
T II -	Tyrosinaemia Type II
T2D -	T2-Defiency
T-ALL -	T-cell Acute Lymphoblastic Leukemia
TD -	Tangier Disease
TSC -	Tuberous Sclerosis Complex
VBD -	van Buchem Disease
VHL -	Von Hippel-Lindau Disease
VWD -	von Willebrand Disease
WAS -	Wiskott-Aldrich syndrome
WS IV -	Waardenburg Syndrome type IV

XLA -	X-linked Agammaglobulinemia
XLG -	X-linked Liver Glycogenosis
XMR -	X-Linked Mental Retardation
αT -	Alpha-thalassemia

(2)

Journal Titles

AJHG -	*The American Journal of Human Genetics*
AJMG -	*American Journal of Medical Genetics*
B -	*Blood*
BCMD-	*Blood Cells, Molecules, and Diseases*
BCRT -	*Breast Cancer Research and Treatment*
BJH -	*British Journal of Haematology*
BJN -	*Brain. A Journal of Neurology*
BMCC -	*BioMed Central Cancer*
BMCG -	*BioMed Central Genomics*
BMCMG -	*BioMed Central Medical Genetics*
BRI -	*BioMed Research International*
CCA -	*Clinica Chimica Acta*
CCG -	*Circulation. Cardiovascular Genetics.*
CE -	*Clinical Endocrinology*
CG -	*Clinical Genetics*
CGC -	*Cancer Genetics and Cytogenetics*
CGR -	*Cytogenetic and Genome Research*
CPR -	*Cancer Prevention Research*
CR -	*Cancer Research*
ED -	*Experimental Dermatology*
EJC -	*European Journal of Cancer*
EJHG -	*European Journal of Human Genetics*
EJMG -	*European Journal of Medical Genetics*
FN -	*Frontiers in Neurology*

Studies Linking *Alu*-related Deletions to Disease Phenotypes

G -	*Gastroenterology*
GCC -	*Genes, Chromosomes & Cancer*
GE -	Gene
GM -	*Genetics in Medicine*
HG -	*Human Genetics*
HGVS -	*Human Genome Variation Society*
HM -	*Human Mutation*
HMG -	*Human Molecular Genetics*
IJC -	*International Journal of Cancer*
IOVS -	*Investigative Ophthalmology and Visual Science*
JBMR -	*Journal of Bone and Mineral Research*
JHG -	*Journal of Human Genetics*
JID -	*The Journal of Investigative Dermatology*
JIMD -	*Journal of Inherited Metabolic Disease*
JJCO -	*Japanese Journal of Clinical Oncology*
JMG -	*Journal of Medical Genetics*
JSBMB -	*Journal of Steroid Biochemistry and Molecular Biology*
JTH-	*Journal of Thrombosis and Haemostasis*
MGM -	*Molecular Genetics and Metabolism*
MR -	*Mutation Research*
N -	*Neurogenetics*
NG -	*Nature Genetics*
O -	*Oncogene*
T -	*Transfusion*

====================================
