# Supplementary material for: A Comparison of 100 Human Genes Using an Alu Element-Based Instability Model
Source: PLoS One. 2013 Jun 3;8(6):e65188. doi: 10.1371/journal.pone.0065188 (PMC3670932; doi:10.1371/journal.pone.0065188)
Supplement: Table S2 — Raw and fitted I∶D ratios for the ten spacer size groupings for Type 1 Alu pairs. (PDF) [file pone.0065188.s002.pdf]

Raw and fitted I:D ratios for the ten spacer size groupings for Type 1 *Alu* pairs (*Alu* length = 275-325 bp, hg19)

APSN	0-5th Percentile			6-15th Percentile			16-25th Percentile			26-35th Percentile			36-45th Percentile			46-55th Percentile			56-65th Percentile			66-75th Percentile			76-85th Percentile			86-95th Percentile		
	Median Spacer Size (bp)	Actual I:D Ratio	Fitted I:D Ratio	Median Spacer Size (bp)	Actual I:D Ratio	Fitted I:D Ratio	Median Spacer Size (bp)	Actual I:D Ratio	Fitted I:D Ratio	Median Spacer Size (bp)	Actual I:D Ratio	Fitted I:D Ratio	Median Spacer Size (bp)	Actual I:D Ratio	Fitted I:D Ratio	Median Spacer Size (bp)	Actual I:D Ratio	Fitted I:D Ratio	Median Spacer Size (bp)	Actual I:D Ratio	Fitted I:D Ratio	Median Spacer Size (bp)	Actual I:D Ratio	Fitted I:D Ratio	Median Spacer Size (bp)	Actual I:D Ratio	Fitted I:D Ratio	Median Spacer Size (bp)	Actual I:D Ratio	Fitted I:D Ratio
1	100	0.8007	0.7994	265	0.9194	0.9194	470	0.9117	0.9129	691	0.9236	0.9176	965	0.8848	0.8780	1,337	0.8339	0.8530	1,890	0.8452	0.8652	2,755	0.9074	0.8916	4,280	0.8999	0.9159	7,538	1.0042	0.9604
2	344	0.9194	0.9344	550	0.9768	0.9706	809	0.9462	0.9328	1,092	0.9098	0.9150	1,447	0.9006	0.8851	1,926	0.8759	0.8634	2,614	0.8698	0.8738	3,758	0.9140	0.8986	5,821	0.9224	0.9191	10,299	0.9039	0.9569
3	632	0.9751	0.9704	935	0.9471	0.9413	1,291	0.8922	0.9271	1,676	0.9063	0.9131	2,148	0.8994	0.8923	2,796	0.8808	0.8740	3,732	0.8952	0.8834	5,279	0.9232	0.9062	8,126	0.9163	0.9231	14,072	0.9453	0.9544
4	972	1.0094	0.9814	1,391	0.9282	0.9254	1,853	0.9307	0.9155	2,354	0.9040	0.9119	2,978	0.8930	0.8986	3,812	0.8909	0.8830	5,041	0.8837	0.8916	7,030	0.9192	0.9127	10,569	0.9123	0.9265	18,160	0.9478	0.9533
5	1,324	0.9920	0.9816	1,866	0.9177	0.9170	2,447	0.9276	0.9059	3,081	0.9063	0.9113	3,851	0.8841	0.9037	4,909	0.8876	0.8904	6,417	0.9132	0.8982	8,867	0.9283	0.9180	13,265	0.9346	0.9297	22,676	0.9473	0.9528
6	1,694	0.9800	0.9770	2,355	0.9019	0.9121	3,060	0.9086	0.8995	3,819	0.9466	0.9109	4,742	0.8843	0.9080	5,988	0.8822	0.8962	7,786	0.8836	0.9035	10,670	0.9062	0.9222	15,844	0.9783	0.9324	26,666	0.9630	0.9529
7	2,091	0.9520	0.9699	2,869	0.8808	0.9093	3,692	0.8774	0.8958	4,585	0.9074	0.9106	5,677	0.8782	0.9118	7,104	0.8843	0.9013	9,218	0.8825	0.9082	12,563	0.9117	0.9260	18,722	0.9099	0.9350	31,551	0.9687	0.9532
8	2,500	0.9419	0.9616	3,398	0.8949	0.9078	4,343	0.8935	0.8944	5,381	0.9144	0.9105	6,650	0.9143	0.9151	8,299	0.9086	0.9059	10,711	0.9287	0.9123	14,431	0.9257	0.9292	21,497	0.9601	0.9372	36,034	0.9431	0.9537
9	2,912	0.9458	0.9529	3,931	0.9183	0.9072	5,009	0.8873	0.8950	6,185	0.9025	0.9105	7,609	0.9236	0.9181	9,472	0.9221	0.9099	12,189	0.9159	0.9159	16,401	0.8822	0.9322	24,354	0.9214	0.9393	40,625	0.9563	0.9542
10	3,337	0.9453	0.9439	4,475	0.8944	0.9072	5,664	0.8943	0.8971	6,945	0.8927	0.9105	8,538	0.9392	0.9206	10,597	0.9166	0.9132	13,547	0.9368	0.9189	18,262	0.9191	0.9347	26,899	0.9569	0.9410	45,150	0.9901	0.9549
11	3,766	0.9259	0.9350	5,039	0.9030	0.9077	6,368	0.9037	0.9006	7,801	0.9058	0.9105	9,517	0.9310	0.9231	11,769	0.9277	0.9164	15,042	0.9244	0.9218	20,186	0.9313	0.9370	29,726	0.9652	0.9428	49,917	0.9537	0.9556
12	4,219	0.9162	0.9258	5,602	0.9175	0.9084	7,065	0.8998	0.9051	8,640	0.9234	0.9106	10,521	0.9039	0.9253	12,984	0.8858	0.9193	16,561	0.9217	0.9246	22,228	0.9263	0.9393	32,717	0.9783	0.9445	54,434	0.9788	0.9563
13	4,672	0.9483	0.9300	6,163	0.8912	0.9094	7,738	0.9248	0.9103	9,437	0.9359	0.9106	11,486	0.9006	0.9273	14,175	0.8995	0.9220	18,024	0.8899	0.9270	24,094	0.9330	0.9412	35,384	0.9567	0.9460	58,961	0.9295	0.9570
14	5,134	0.9526	0.9329	6,757	0.9286	0.9105	8,432	0.9042	0.9163	10,259	0.8857	0.9107	12,439	0.9281	0.9292	15,280	0.9368	0.9243	19,363	0.9153	0.9290	25,948	0.9463	0.9429	38,135	0.9429	0.9474	63,302	0.9540	0.9577
15	5,574	0.9543	0.9354	7,329	0.9159	0.9118	9,144	0.9335	0.9230	11,136	0.9016	0.9108	13,507	0.9338	0.9311	16,557	0.9112	0.9267	20,875	0.9241	0.9311	27,999	0.9263	0.9447	41,024	0.9391	0.9488	68,492	0.9530	0.9585
16	6,041	0.9793	0.9379	7,894	0.9139	0.9131	9,871	0.9196	0.9269	11,963	0.9080	0.9130	14,480	0.9502	0.9332	17,745	0.9157	0.9289	22,424	0.9082	0.9332	30,051	0.9477	0.9464	43,919	0.9371	0.9501	73,690	0.9884	0.9594
17	6,506	0.9395	0.9402	8,489	0.9234	0.9146	10,574	0.9588	0.9291	12,797	0.9374	0.9157	15,489	0.9676	0.9338	18,951	0.9335	0.9309	23,880	0.9720	0.9350	31,811	0.9392	0.9477	46,623	0.9508	0.9513	77,558	0.9515	0.9600
18	6,980	0.9216	0.9424	9,107	0.9518	0.9161	11,299	0.9336	0.9313	13,652	0.8990	0.9183	16,523	0.9586	0.9345	20,144	0.9517	0.9327	25,443	0.9350	0.9368	33,841	0.9495	0.9492	49,711	0.9412	0.9525	82,510	0.9600	0.9607
19	7,463	0.9439	0.9445	9,679	0.9483	0.9176	11,988	0.9168	0.9332	14,459	0.9178	0.9206	17,476	0.9471	0.9351	21,315	0.9467	0.9345	26,885	0.9503	0.9383	35,649	0.9377	0.9504	52,505	0.9256	0.9536	86,868	0.9513	0.9614
20	7,960	0.9581	0.9465	10,300	0.9201	0.9192	12,734	0.8894	0.9352	15,345	0.9479	0.9230	18,481	0.9499	0.9358	22,571	0.9653	0.9362	28,444	0.9401	0.9400	37,705	0.9704	0.9517	55,237	0.9489	0.9547	91,524	0.9744	0.9621
21	8,412	0.9655	0.9482	10,877	0.9287	0.9207	13,419	0.9379	0.9369	16,165	0.9619	0.9251	19,415	0.9592	0.9363	23,715	0.9382	0.9378	29,851	0.9528	0.9414	39,509	0.9453	0.9528	58,081	0.9552	0.9557	96,502	0.9542	0.9629
22	8,890	0.9337	0.9499	11,484	0.9294	0.9223	14,123	0.9369	0.9386	17,008	0.9679	0.9272	20,430	0.9537	0.9370	24,913	0.9383	0.9393	31,346	0.9227	0.9428	41,534	0.9694	0.9540	61,037	0.9402	0.9567	101,169	0.9289	0.9636
23	9,390	0.9700	0.9516	12,102	0.9079	0.9240	14,900	0.9399	0.9403	17,870	0.9579	0.9292	21,474	0.9645	0.9377	26,141	0.9591	0.9408	32,902	0.8980	0.9442	43,486	0.9750	0.9551	63,784	0.9693	0.9577	105,759	0.9578	0.9642
24	9,853	1.0072	0.9531	12,711	0.9088	0.9256	15,630	0.9521	0.9419	18,746	0.9510	0.9311	22,481	0.9045	0.9383	27,421	0.9422	0.9423	34,365	0.9605	0.9454	45,434	0.9578	0.9562	66,616	0.9562	0.9586	110,468	0.9720	0.9649
25	10,343	0.9742	0.9546	13,314	0.9271	0.9272	16,345	0.9597	0.9434	19,600	0.9423	0.9329	23,494	0.9409	0.9390	28,596	0.9517	0.9436	35,885	0.9472	0.9467	47,402	0.9691	0.9572	69,655	0.9426	0.9595	115,375	0.9615	0.9656
26	10,836	0.9437	0.9561	13,905	0.9491	0.9287	17,004	0.9609	0.9447	20,385	0.9427	0.9345	24,434	0.9238	0.9396	29,704	0.9343	0.9447	37,271	0.9360	0.9478	49,231	0.9643	0.9581	72,041	0.9836	0.9603	119,496	0.9716	0.9662
27	11,319	0.9724	0.9575	14,535	0.9323	0.9304	17,812	0.9484	0.9462	21,342	0.9469	0.9364	25,564	0.9555	0.9403	31,058	0.9468	0.9461	38,970	0.9391	0.9491	51,524	0.9603	0.9592	75,575	0.9705	0.9613	124,295	0.9759	0.9668
28	11,853	0.9424	0.9589	15,174	0.9074	0.9320	18,530	0.9327	0.9475	22,210	0.9429	0.9381	26,592	0.9505	0.9409	32,227	0.9551	0.9473	40,453	0.9683	0.9501	53,395	1.0016	0.9600	77,892	0.9552	0.9620	128,840	0.9598	0.9674
29	12,320	0.9546	0.9601	15,760	0.9395	0.9335	19,295	0.9320	0.9489	23,064	0.9486	0.9396	27,608	0.9533	0.9416	33,544	0.9723	0.9485	41,859	0.9515	0.9511	55,386	0.9549	0.9609	80,957	0.9637	0.9628	133,782	1.0005	0.9681
30	12,812	0.9736	0.9614	16,379	0.8980	0.9351	20,006	0.9578	0.9501	23,870	0.9236	0.9410	28,534	0.9417	0.9422	34,717	0.9526	0.9496	43,327	0.9834	0.9521	57,351	0.9385	0.9617	83,520	0.9786	0.9635	138,266	0.9860	0.9687
31	13,376	0.9364	0.9627	17,001	0.9277	0.9367	20,764	0.9715	0.9513	24,788	0.9055	0.9426	29,600	0.9644	0.9429	36,026	0.9545	0.9508	44,999	0.9774	0.9532	59,459	0.9486	0.9626	86,892	0.9764	0.9644	142,612	0.9877	0.9692
32	13,839	0.9130	0.9638	17,576	0.9289	0.9381	21,508	0.9521	0.9525	25,696	0.9350	0.9441	30,670	0.9598	0.9435	37,149	0.9452	0.9517	46,298	0.9427	0.9541	61,336	0.9630	0.9634	89,401	0.9647	0.9651	147,465	0.9772	0.9699
33	14,386	0.9118	0.9650	18,214	0.9506	0.9397	22,215	0.9606	0.9536	26,568	0.9420	0.9454	31,712	0.9324	0.9442	38,465	0.9801	0.9528	47,904	0.9449	0.9551	63,159	0.9320	0.9641	92,576	0.9514	0.9659	152,367	0.9439	0.9705
34	14,915	0.9817	0.9662	18,854	0.9527	0.9413	23,018	0.9633	0.9548	27,396	0.9269	0.9467	32,736	0.9318	0.9449	39,692	0.9928	0.9538	49,588	0.9665	0.9561	65,606	0.9418	0.9650	95,918	0.9838	0.9667	157,912	0.9601	0.9712
35	15,381	0.9854	0.9671	19,427	0.9451	0.9427	23,697	0.9526	0.9557	28,188	0.9336	0.9479	33,652	0.9489	0.9454	40,711	0.9719	0.9546	50,941	0.9564	0.9569	67,198	0.9561	0.9655	97,871	0.9805	0.9671	160,843	0.9526	0.9715
36	15,962	0.9464	0.9683	20,073	0.9821	0.9442	24,472	0.9799	0.9568	29,152	0.9329	0.9493	34,750	0.9453	0.9461	42,063	0.9327	0.9556	52,591	0.9768	0.9578	69,346	0.9808	0.9663	101,068	1.0127	0.9679	166,334	0.9711	0.9722
37	16,385	0.9368	0.9691	20,702	0.9416	0.9458	25,245	0.9856	0.9579	30,034	0.9427	0.9505	35,740	0.9249	0.9468	43,230	0.9218	0.9565	54,069	0.9777	0.9586	71,329	0.9696	0.9670	104,361	0.9587	0.9686	171,223	0.9884	0.9727
38	16,900	0.9381	0.9701	21,303	0.9174	0.9472	25,978	0.9565	0.9588	30,926	0.9334	0.9517	36,808	0.9439	0.9475	44,572	0.9208	0.9575	55,738	0.9575	0.9595	73,504	0.9822	0.9677	107,615	0.9963	0.9693	176,317	0.9880	0.9733
39	17,439	0.9640	0.9711	21,928	0.9334	0.9487	26,719	0.9511	0.9598	31,745	0.9339	0.9528	37,726	0.9267	0.9480	45,680	0.9480	0.9582	57,150	0.9931	0.9602	75,361	0.9641	0.9683	110,293	0.9599	0.9699	181,108	0.9752	0.9739
40	17,947	0.9706	0.9720	22,604	0.9619	0.9502	27,608	0.9386	0.9609	32,761	0.9444	0.9541	38,915	0.9593	0.9488	46,889	0.9380	0.9591	58,752	1.0055	0.9610	77,490	0.9762	0.9690	113,133	0.9810	0.9705	185,418	0.9328	0.9744
41	18,400	0.9748	0.9728	23,180	0.9375	0.9516	28,215	0.9530	0.9616	33,483	0.9652	0.9550	39,798	0.9703	0.9494	48,019	0.9624	0.9598	59,927	0.9881	0.9616	79,083	0.9597	0.9695	115,843	0.9671	0.9711	189,553	0.9917	0.9748
42	18,989	1.0036	0.9739	23,842	0.9659	0.9531	29,005	0.9641	0.9625	34,449	0.9468	0.9562	40,929	0.9828	0.9501	49,328	0.9818	0.9607	61,700	0.9742	0.9625	81,220	0.9926	0.9701	119,142	1.0021	0.9718	195,023	1.0023	0.9754
43	19,505	0.9617	0.9747	24,439	0.9594	0.9544	29,831	0.9570	0.9635	35,435	0.9287	0.9574	42,025	1.0115	0.9508	50,578	0.9743	0.9614	63,297	0.9586	0.9632	83,306	0.9809	0.9707	122,210	0.9941	0.9724	200,344	0.9532	0.9760
44	20,052	0.9538	0.9756	25,095	0.9461	0.9559	30,522	0.9496	0.9642	36,284	0.9374	0.9584	42,953	0.9623	0.9514	51,786	0.9630	0.9622	64,852	0.9458	0.9639	85,096	0.9917	0.9712	125,176	0.9728	0.9729	205,283	0.9707	0.9765
45	20,584	0.9689	0.9764	25,751	0.9514	0.9573	31,331	0.9337	0.9652	37,127	0.9578	0.9594	44,020	0.9569	0.9521	53,015	0.9669	0.9629	66,314	1.0026	0.9646	86,886	0.9791	0.9718	127,473	0.9448	0.9734	209,826	1.0201	0.9770
46	21,075	0.9892	0.9772	26,383	0.9608	0.9587	32,169	0.9582	0.9662	38,157	1.0058	0.9605	45,229	0.9696	0.9528	54,474	0.9819	0.9638	68,095	0.9621	0.9654	89,353	0.9630	0.9724	130,922	0.9444	0.9740	213,098	0.9751	0.9774
47	21,636	0.9668	0.9780	26,961	0.9832	0.9600	32,833	0.9617	0.9669	39,008	0.9593	0.9614	46,205	0.9498	0.9535	55,658	0.9775	0.9645	69,523	0.9691	0.9660	91,147	0.9558	0.9729	133,329	0.9506	0.9745	218,419	0.9629	0.9779
48	22,156	0.9593	0.9788	27,685	0.9687	0.9615	33,575	0.9826	0.9678	39,894	0.9655	0.9624	47,313	0.9612	0.9542	56,849	0.9699	0.9652	71,081	0.9748	0.9666	93,208	0.9609	0.9735	135,860	0.9647	0.9749	223,001	0.9634	0.9784
49	22,656	0.9893	0.9795	28,241	0.9612	0.9627	34,299	0.9724	0.9685	40,794	0.9865	0.9633	48,357	0.9804	0.9548	58,117	0.9770	0.9659	72,562	0.9585	0.9673	95,145	0.9790	0.9740	138,712	0.9422	0.9755	227,791	0.9722	0.9789
50	23,145	0.9552	0.9802	28,951	0.9597	0.9642	35,196	0.9521	0.9695	41,787	0.9785	0.9643	49,373	0.9546	0.9555	59,363	0.9835	0.9665	74,011	0.9625	0.9678	97,043	0.9765	0.9744	141,714	0.9662	0.9760	231,295	0.9616	0.9792
51	23,715	0.9828	0.9810	29,513	0.9671	0.9654	35,824	0.9563	0.9701	42,624	0.9747	0.9652	50,318	0.9730	0.9561	60,496	1.0023	0.9671	75,766	0.9665	0.9685	99,487	0.9784	0.9750	145,126	0.9740	0.9766	236,850	0.9958	0.9798
52	24,244	0.9873	0.9817	30,213	0.9773	0.9667	36,626	0.9881	0.9710	43,498	0.9655	0.9660	51,349	0.9737	0.9567	61,799	0.9828	0.9678	77,413	0.9344	0.9692	101,423	0.9997	0.9755	147,374	0.9652	0.9770	240,314	0.9845	0.9801
53	24,701	0.9834	0.9823	30,799	0.9838	0.9675	37,335	0.9589	0.9717	44,392	0.9486	0.9669	52,451	0.9645	0.9574	63,167	0.9452	0.9685	78,937	0.9879	0.9697	103,601	0.9866	0.9760	151,159	0.9825	0.9776	246,671	0.9911	0.9807
54	25,257	0.9500	0.9830	31,467	0.9649	0.9684	38,129	0.9670	0.9724	45,279	0.9428	0.9677	53,560	0.9793	0.9582	64,453	0.9194	0.9691	80,596	0.9834	0.9704	105,698	0.9612	0.9765	153,856	0.9811	0.9780	249,886	0.9769	0.9810
55	25,769	0.9464	0.9837	32,061	0.9368	0.9691	38,877	0.9730	0.9732	46,099	0.9650	0.9685	54,519	0.9540	0.9588	65,578	0.9736	0.9697	81,904	1.0057	0.9708	107,442	1.0034	0.9769	156,369	0.9888	0.9784	254,587	1.0018	0.9815
56	26,281	0.9882	0.9843	32,702	0.9895	0.9700	39,619	0.9859	0.9739	46,945	0.9353	0.9692	55,423	0.9419	0.9593	66,581	0.9722	0.9702	83,084	0.9463	0.9713	109,173	0.9696	0.9773	159,235	0.9790	0.9789	260,229	0.9548	0.9820
57	26,735	0.9805	0.9848	33,377	0.9876	0.9708	40,449	0.9712	0.9746	47,943	0.9876	0.9701	56,580	0.9460	0.9601	68,025	0.9671	0.9709	84,905	0.9592	0.9719	111,271	0.9850	0.9778	161,968	0.9702	0.9793	263,552	0.9841	0.9823
58	27,372	0.9876	0.9856	34,008	0.9660	0.9716	41,236	1.0003	0.9753	48,826	0.9499	0.9709	57,570	0.9665	0.9607	69,271	0.9875	0.9715	86,607	0.9497	0.9725	113,370	1.0311	0.9782	164,906	0.9746	0.9798	267,196	0.9790	0.9826
59	27,911	0.9519	0.9862	34,635	0.9947	0.9723	42,065	0.9679	0.9761	49,792	0.9801	0.9717	58,698	0.9892	0.9614	70,493	0.9667	0.9720	88,297	0.9605	0.9731	115,913	1.0005	0.9788	167,848	0.9692	0.9802	272,890	0.9879	0.9832
60	28,405	0.9747	0.9868	35,267	0.9806	0.9731	42,836	0.9745	0.9768	50,667	0.9734	0.9725	59,656	0.9936	0.9620	71,601	0.9414	0.9725	89,430	1.0083	0.9734	117,934	0.9853	0.9792	171,349	0.9503	0.9808	277,612	0.9824	0.9836
61	29,039	0.9722	0.9875	36,025	0.9767	0.9740	43,638	0.9737	0.9774	51,687	0.9758	0.9733	60,795	0.9496	0.9628	73,053	0.9773	0.9732	91,030	1.0044	0.9740	119,994	0.9813	0.9796	174,285	0.9880	0.9812	283,196	0.9663	0.9841
62	29,467	0.9918	0.9880	36,587	1.0015	0.9746	44,347	0.9733	0.9780	52,437	0.9781	0.9739	61,616	0.9585	0.9633	74,075	0.9827	0.9736	92,366	1.0305	0.9744	121,898	0.9724	0.9800	178,426	0.9618	0.9818	288,350	0.9817	0.9845
63	30,086	0.9866	0.9887	37,288	0.9701	0.9754	45,185	0.9695	0.9787	53,370	0.9814	0.9747	62,830	0.9966	0.9641	75,626	0.9576	0.9743	94,643	0.9732	0.9751	124,437	0.9676	0.9805	180,410	0.9647	0.9821	294,569	0.9824	0.9851
64	30,658	0.9846	0.9893	37,957	0.9945	0.9762	45,949	0.9568	0.9794	54,276	0.9897	0.9754	63,880	0.9775	0.9647	76,820	0.9816	0.9748	96,107	1.0042	0.9756	126,382	0.9709	0.9809	182,987	0.9721	0.9825	298,371	0.9571	0.9854
65	31,107	1.0214	0.9898	38,500	0.9982	0.9767	46,732	0.9890	0.9800	55,188	0.9781	0.9761	64,878	0.9994	0.9654	78,121	0.9858	0.9753	97,678	0.9664	0.9761	128,375	0.9702	0.9813	185,872	0.9795	0.9829	302,140	0.9857	0.9857
66	31,659	1.0249	0.9903	39,162	1.0124	0.9775	47,461	1.0032	0.9806	56,248	0.9906	0.9769	66,021	0.9889	0.9661	79,433	0.9768	0.9758	99,678	1.0058	0.9767	131,166	0.9890	0.9818	189,691	0.9795	0.9834	302,140	0.9857	0.9857
67	32,183	1.0053	0.9909	39,894	0.9590	0.9782	48,269	0.9578	0.9812	57,058	0.9973	0.9775	67,002	0.9899	0.9667	80,725	0.9709	0.9764	101,167	0.9832	0.9771	132,891	1.0120	0.9821	191,507	0.9733	0.9837	312,866	1.0039	0.9866
68	32,699	1.0324	0.9914	40,587	0.9894	0.9783	49,050	0.9401	0.9818	58,012	0.9914	0.9783	68,138	0.9836	0.9675	82,012	0.9675	0.9769	102,746	0.9951	0.9776	135,023	0.9693	0.9825	195,962	0.9843	0.9843	316,456	0.9745	0.9869
69	33,240	0.9484	0.9919	41,139	0.9792	0.9785	49,787	0.9730	0.9824	58,806	0.9695	0.9788	69,103	0.9803	0.9681	83,169	0.9869	0.9773	104,124	1.0024	0.9780	136,584	0.9436	0.9828	197,717	1.0186	0.9845	320,322	0.9868	0.9872
70	33,653	0.9787	0.9923	41,809	0.9561	0.9789	50,577	0.9400	0.9829	59,707	0.9755	0.9795	70,108	0.9459	0.9687	84,324	0.9557	0.9778	105,546	0.9606	0.9784	138,379	0.9631	0.9831	199,732	0.9905	0.9848	323,337	0.9722	0.9875
71	34,268	0.9301	0.9929	42,528	0.9705	0.9793	51,397	0.9807	0.9835	60,706	0.9576	0.9802	71,294	0.9744	0.9695	86,008	0.9643	0.9784	107,531	0.9690	0.9789	141,165	0.9932	0.9836	203,925	0.9705	0.9853	329,838	1.0034	0.9880
72	34,814	0.9599	0.9934	43,126	0.9797	0.9796	52,214	0.9748	0.9841	61,568	0.9139	0.9808	72,264	0.9619	0.9701	87,263	0.9561	0.9789	109,132	0.9947	0.9794	143,076	0.9972	0.9839	207,236	0.9759	0.9857	335,591	1.0219	0.9885
73	35,371	1.0125	0.9939	43,752	0.9714	0.9800	52,945	0.9918	0.9847	62,301	0.9606	0.9813	73,247	0.9639	0.9707	88,314	0.9507	0.9793	110,279	0.9794	0.9797	144,582	1.0015	0.9842	209,233	0.9737	0.9860	340,254	0.9982	0.9888
74	36,019	1.0167	0.9945	44,489	0.9668	0.9804	53,818	1.0108	0.9853	63,434	0.9616	0.9821	74,408	0.9605	0.9715	89,678	0.9694	0.9797	111,874	0.9836	0.9801	147,602	0.9709	0.9847	213,721	0.9817	0.9866	345,504	1.0353	0.9892
75	36,523	1.0070	0.9950	45,180	0.9571	0.9808	54,554	1.0103	0.9858	64,251	1.0003	0.9826	75,632	1.0038	0.9722	91,197	0.9806	0.9803	113,775	0.9662	0.9806	149,397	0.9633	0.9850	215,569	0.9855	0.9868	349,203	0.9388	0.9895
76	37,040	0.9908	0.9954	45,765	0.9447	0.9812	55,355	0.9987	0.9863	65,236	0.9828	0.9833	76,631	0.9877	0.9729	92,345	1.0157	0.9807	115,256	0.9648	0.9810	150,796	0.9710	0.9852	218,176	0.9721	0.9871	354,183	1.0052	0.9899
77	37,547	0.9402	0.9959	46,466	0.9713	0.9816	56,127	0.9881	0.9868	66,176	0.9890	0.9839	77,653	0.9457	0.9735	93,588	0.9874	0.9811	117,109	0.9984	0.9815	153,598	0.9712	0.9857	221,347	0.9560	0.9875	357,570	0.9855	0.9902
78	38,207	0.9635	0.9965	47,156	1.0037	0.9821	56,996	0.9937	0.9874	67,316	0.9702	0.9846	78,978	0.9887	0.9744	95,045	0.9801	0.9816	118,928	0.9740	0.9820	155,814	0.9937	0.9860	225,080	0.9877	0.9879	363,649	0.9877	0.9906
79	38,725	0.9713	0.9969	47,901	1.0091	0.9825	57,939	1.0003	0.9880	68,284	1.0083	0.9852	80,171	0.9998	0.9751	96,519	1.0083	0.9821	120,891	0.9509	0.9825	158,861	0.9945	0.9865	228,225	0.9814	0.9883	367,386	1.0062	0.9909
80	39,258	1.0129	0.9973	48,480	1.0145	0.9829	58,539	0.9885	0.9884	69,025	1.0044	0.9857	81,066	0.9586	0.9757	97,659	1.0065	0.9825	122,175	0.9500	0.9828	160,301	0.9841	0.9867	230,850	0.9943	0.9886	373,220	0.9953	0.9914
81	39,829	1.0420	0.9978	49,095	0.9607	0.9833	59,309	1.0159	0.9889	69,854	0.9853	0.9862	82,084	0.9507	0.9764	98,767	0.9865	0.9829	123,542	0.9916	0.9831	162,870	0.9885	0.9871	234,246	1.0000	0.9890	377,485	0.9727	0.9917
82	40,446	1.0123	0.9983	49,882	0.9871	0.9838	60,065	1.0481	0.9894	70,550	0.9958	0.9867	82,853	0.9858	0.9769	99,684	0.9914	0.9832	124,590	0.9719	0.9834	163,922	0.9871	0.9873	236,341	1.0192	0.9893	382,122	0.9974	0.9920
83	40,909	1.0379	0.9987	50,464	0.9740	0.9842	61,008	1.0177	0.9900	71,722	0.9734	0.9874	84,141	0.9905	0.9777	101,227	0.9620	0.9837	126,740	0.9836	0.9839	166,589	1.0226	0.9876	238,424	0.9873	0.9895	385,942	0.9713	0.9923
84	41,422	1.0246	0.9991	51,055	0.9930	0.9846	61,628	1.0193	0.9904	72,574	0.9688	0.9879	85,243	0.9733	0.9784	102,557	0.9688	0.9841	127,762	1.0015	0.9841	167,851	0.9769	0.9878	241,254	1.0003	0.9898	389,382	1.0158	0.9925
85	42,022	0.9879	0.9996	51,760	0.9765	0.9851	62,460	0.9966	0.9909	73,442	1.0026	0.9884	86,319	0.9647	0.9791	103,787	0.9637	0.9845	129,791	0.9729	0.9846	171,012	1.0147	0.9883	245,783	0.9992	0.9903	397,082	0.9759	0.9931
86	42,609	1.0545	1.0000	52,514	0.9868	0.9857	63,284	0.9778	0.9914	74,415	0.9951	0.9890	87,380	0.9848	0.9797	105,150	0.9484	0.9849	131,131	0.9754	0.9849	173,164	0.9956	0.9886	249,264	1.0195	0.9907	400,738	1.0085	0.9933
87	43,253	0.9964	1.0005	53,160	0.9666	0.9862	64,112	0.9745	0.9918	75,378	0.9820	0.9895	88,596	0.9749	0.9805	106,434	0.9683	0.9853	133,021	0.9628	0.9853	175,438	0.9864	0.9889	252,220	0.9795	0.9910	405,615	0.9845	0.9937
88	43,714	0.9594	1.0009	53,884	0.9391	0.9867	65,017	0.9708	0.9924	76,415	0.9642	0.9901	89,675	1.0111	0.9812	108,073	0.9835	0.9858	135,478	1.0018	0.9859	177,907	0.9908	0.9893	254,939	0.9984	0.9913	409,825	1.0093	0.9940
89	44,282	0.9957	1.0013	54,505	0.9801	0.9872	65,756	0.9670	0.9928	77,203	0.9972	0.9905	90,691	0.9787	0.9819	108,969	1.0062	0.9861	136,424	0.9990	0.9861	179,335	0.9671	0.9895	257,231	1.0139	0.9916	412,400	1.0052	0.9942
90	44,988	0.9865	1.0018	55,192	0.9992	0.9877	66,544	0.9779	0.9932	78,082	1.0180	0.9910	91,806	0.9606	0.9826	110,363	1.0158	0.9865	138,129	0.9743	0.9865	181,723	0.9922	0.9898	260,670	0.9791	0.9920	416,677	0.9831	0.9945
91	45,471	1.0173	1.0022	55,803	1.0000	0.9882	67,418	0.9858	0.9937	79,007	1.0110	0.9916	92,956	0.9567	0.9833	111,667	0.9867	0.9868	139,787	0.9665	0.9868	183,793	1.0064	0.9901	263,134	0.9669	0.9922	421,700	1.0103	0.9948
92	45,861	1.0089	1.0024	56,460	1.0015	0.9887	68,198	0.9756	0.9942	79,880	0.9697	0.9920	94,101	0.9707	0.9840	112,986	0.9780	0.9872	141,339	0.9767	0.9872	185,892	1.0054	0.9904	266,091	1.0114	0.9925	427,910	0.9821	0.9952
93	46,544	1.0090	1.0029	57,200	0.9731	0.9893	68,962	0.9880	0.9946	80,854	0.9977	0.9926	95,249	0.9848	0.9848	114,255	0.9723	0.9876	143,090	1.0026	0.9875	188,028	0.9997	0.9906	269,367	1.0142	0.9929	431,187	0.9872	0.9954
94	47,049	1.0184	1.0033	57,860	0.9848	0.9898	69,862	1.0103	0.9951	81,902	1.0023	0.9931	96,439	0.9997	0.9855	115,674	0.9589	0.9880	144,931	0.9854	0.9879	190,717	0.9885	0.9910	274,291	0.9711	0.9934	435,478	0.9885	0.9957
95	47,591	1.0258	1.0037	58,587	1.0140	0.9904	70,575	0.9888	0.9955	82,732	0.9821	0.9935	97,487	1.0049	0.9862	117,213	0.9980	0.9884	146,674	0.9539	0.9883	193,895	1.0010	0.9914	277,831	1.0070	0.9937	441,046	0.9901	0.9961
96	48,190	1.0220	1.0041	59,295	1.0123	0.9910	71,392	0.9946	0.9959	83,568	1.0016	0.9940	98,260	0.9824	0.9867	117,868	1.0194	0.9886	147,596	0.9880	0.9885	194,524	1.0348	0.9915	278,704	0.9872	0.9938	445,716	1.0047	0.9964
97	48,722	1.0187	1.0044	59,878	1.0094	0.9915	72,272	1.0161	0.9964	84,682	0.9987	0.9946	99,495	0.9800	0.9875	119,295	0.9881	0.9890	149,230	0.9728	0.9888	196,061	1.0116	0.9917	282,427	0.9819	0.9942	451,274	0.9715	0.9968
98	49,261	1.0234	1.0048	60,567	0.9855	0.9920	72,941	0.9438	0.9967	85,673	0.9994	0.9951	100,752	0.9706	0.9883	120,916	0.9788	0.9894	151,379	0.9604	0.9892	199,041	0.9742	0.9920	286,673	0.9676	0.9946	457,397	0.9974	0.9972
99	49,873	1.0258	1.0052	61,121	1.0019	0.9925	73,600	0.9590	0.9971	86,353	1.0080	0.9954	101,667	0.9981	0.9889	121,983	0.9743	0.9897	152,304	0.9840	0.9894	199,956	0.9632	0.9922	287,219	0.9961	0.9947	458,686	1.0165	0.9973
100	50,467	1.0053	1.0056	61,947	0.9943	0.9933	74,470	0.9780	0.9975	87,305	1.0112	0.9959	102,711	0.9875	0.9895	123,315	1.0154	0.9901	153,963	1.0148	0.9897	203,125	0.9645	0.9925	291,252	1.0005	0.9951	464,514	1.0174	0.9976
101	50,941	0.9703	1.0059	62,550	0.9909	0.9938	75,366	1.0184	0.9980	88,183	1.0054	0.9963	103,620	0.9849	0.9901	124,736	0.9899	0.9904	155,672	0.9876	0.9901	205,475	0.9643	0.9928	294,781	0.9878	0.9954	466,383	0.9585	0.9978
102	51,480	1.0425	1.0063	63,155	1.0046	0.9943	76,010	1.0103	0.9983	88,982	1.0069	0.9967	104,954	0.9622	0.9910	126,052	0.9925	0.9908	157,081	0.9922	0.9903	207,472	1.0079	0.9931	297,343	0.9900	0.9956	470,459	0.9674	0.9980
103	52,127	1.0776	1.0067	63,899	0.9898	0.9950	76,911	1.0115	0.9987	90,061	1.0046	0.9972	106,098	0.9739	0.9917	127,459	0.9663	0.9912	159,023	0.9928	0.9907	209,846	0.9935	0.9933	301,686	1.0093	0.9961	475,743	1.0067	0.9984
104	52,692	1.0426	1.0070	64,634	1.0297	0.9957	77,745	1.0166	0.9991	90,960	0.9925	0.9977	107,111	0.9661	0.9923	128,680	0.9744	0.9915	160,782	1.0137	0.9911	212,053	1.0277	0.9936	304,235	1.0184	0.9963	481,410	1.0015	0.9987
105	53,308	1.0232	1.0074	65,292	1.0067	0.9963	78,348	1.0325	0.9994	91,969	1.0010	0.9982	108,514	0.9809	0.9932	130,125	0.9993	0.9918	162,498	1.0029	0.9914	214,400	0.9935	0.9939	307,044	1.0028	0.9966	485,652	0.9773	0.9990
106	53,986	1.0386	1.0078	66,027	0.9609	0.9970	79,207	1.0474	0.9998	92,777	0.9778	0.9985	109,307	1.0024	0.9938	131,198	1.0069	0.9921	163,838	0.9929	0.9916	216,805	0.9580	0.9942	310,171	1.0289	0.9968	492,716	0.9919	0.9994
107	54,350	1.0328	1.0081	66,685	0.9901	0.9976	80,271	1.0250	1.0004	93,900	0.9870	0.9991	110,739	0.9739	0.9947	132,945	0.9887	0.9925	166,082	1.0028	0.9920	219,641	0.9833	0.9945	314,599	1.0271	0.9972	495,613	1.0033	0.9996
108	55,056	1.0123	1.0085	67,513	0.9985	0.9984	81,238	0.9911	1.0008	95,050	1.0065	0.9996	112,024	0.9861	0.9955	134,541	0.9610	0.9929	168,249	0.9846	0.9924	221,532	0.9583	0.9947	317,489	1.0144	0.9975	501,529	1.0250	1.0000
109	55,664	1.0050	1.0088	68,214	1.0090	0.9991	81,890	0.9771	1.0011	95,922	0.9951	1.0000	113,097	0.9874	0.9962	135,797	0.9945	0.9932	169,911	0.9878	0.9927	223,532	1.0083	0.9949	320,531	0.9977	0.9978	507,110	0.9908	1.0003
110	56,093	0.9892	1.0091	68,835	0.9695	0.9997	82,664	0.9638	1.0015	96,905	1.0172	1.0004	114,292	0.9954	0.9969	137,335	1.0021	0.9936	171,842	0.9582	0.9931	226,478	0.9744	0.9952	325,507	0.9962	0.9982	512,027	1.0142	1.0006
111	56,738	0.9382	1.0095	69,581	0.9919	1.0005	83,588	0.9989	1.0019	97,890	1.0244	1.0009	115,337	0.9846	0.9976	138,247	0.9716	0.9938	173,061	0.9676	0.9933	227,824	1.0094	0.9954	327,396	1.0168	0.9984	514,519	1.0128	1.0007
112	57,274	0.9784	1.0098	70,140	1.0231	1.0010	84,194	1.0090	1.0022	98,630	1.0112	1.0012	116,417	0.9739	0.9983	139,678	0.9927	0.9941	174,322	0.9772	0.9935	229,663	0.9841	0.9956	329,214	0.9843	0.9985	521,729	1.0191	1.0012
113	57,809	0.9735	1.0101	70,816	0.9953	1.0017	85,051	1.0147	1.0026	99,479	0.9969	1.0016	117,458	0.9799	0.9990	140,562	1.0415	0.9944	176,396	0.9715	0.9939	232,264	1.0091	0.9959	333,027	0.9771	0.9989	525,322	1.0059	1.0014
114	58,352	1.0438	1.0104	71,660	1.0095	1.0026	85,867	1.0155	1.0029	100,450	1.0047	1.0020	118,392	1.0200	0.9996	141,963	1.0038	0.9947	177,460	0.9778	0.9940	233,572	0.9896	0.9960	334,927	0.9899	0.9990	525,735	0.9909	1.0014
115	58,993	1.0299	1.0108	72,408	0.9977	1.0034	86,782	1.0031	1.0033	101,605	1.0036	1.0025	119,926	0.9995	1.0005	143,872	1.0274	0.9951	180,216	1.0018	0.9945	237,700	1.0016	0.9964	340,248	1.0151	0.9995	536,310	1.0194	1.0020
