# Supplementary material for: A Comparison of 100 Human Genes Using an Alu Element-Based Instability Model
Source: PLoS One. 2013 Jun 3;8(6):e65188. doi: 10.1371/journal.pone.0065188 (PMC3670932; doi:10.1371/journal.pone.0065188)
Supplement: Table S3 — Characteristics of the 50 deletion-prone human cancer genes examined in this study. (PDF) [file pone.0065188.s003.pdf]

Characteristics of 50 deletion-prone human cancer genes

Gene	Locus	Coding Region Length (bp)	Coding Exons	*Alu* Population Across *Alu* Landscape: 250 kbp 5' Flanking	*Alu* Population: Gene	*Alu* Population: 250 kbp 3' Flanking	*Alu* Population: Total	Raw Stability Scores: Coding Exon Scores Lowest	Raw Stability Scores: Coding Exon Scores Highest	Raw Stability Scores: Gene Scores
APC	chr5:112,073,556-112,181,936	136,409	14	77	68	191	336	0.902	0.975	0.709
ARID1A	chr1:27,022,522-27,108,601	84,353	20	375	76	324	775	0.903	0.944	0.755
ATM	chr11:108,093,559-108,239,826	137,884	62	262	82	193	537	0.767	0.970	0.359
BRCA1	chr17:41,196,312-41,277,500	78,419	22	298	138	325	761	0.808	0.903	0.357
BRCA2	chr13:32,889,617-32,973,809	82,310	26	89	55	182	326	0.917	0.964	0.610
BUB1B	chr15:40,453,210-40,513,337	59,939	23	130	60	127	317	0.869	0.951	0.593
CASP8	chr2:202,122,754-202,152,434	20,108	8	271	19	76	366	0.806	0.949	0.749
CDKN1B	chr12:12,870,302-12,875,305	1,107	2	186	0	269	455	0.925	0.953	0.952
CDKN2A	chr9:21,967,751-21,994,490	6,599	3	51	6	57	114	0.977	0.984	0.971
CDKN2C	chr1:51,435,642-51,440,309	3,902	2	145	0	241	386	0.947	0.967	0.965
CYLD	chr16:50,775,961-50,835,846	46,810	16	77	13	69	159	0.942	0.987	0.886
DICER1	chr14:95,552,565-95,608,085	42,961	26	81	6	82	169	0.964	0.992	0.941
FANCA	chr16:89,803,959-89,883,065	78,015	43	252	125	277	654	0.832	0.911	0.301
FANCB	chrX:14,861,529-14,891,184	21,944	8	63	7	29	99	0.974	0.991	0.949
FANCD2	chr3:10,068,113-10,141,344	70,293	42	264	80	301	645	0.847	0.925	0.410
FBXO11	chr2:48,034,059-48,132,932	31,632	22	116	101	245	462	0.879	0.934	0.671
FBXW7	chr4:153,242,410-153,456,185	88,923	11	132	73	79	284	0.985	0.990	0.968
FH	chr1:241,660,857-241,683,085	21,895	10	116	4	69	189	0.973	0.989	0.949
GPC3	chrX:132,669,776-133,119,673	449,325	10	110	175	190	475	0.957	0.979	0.821
IKZF1	chr7:50,344,378-50,472,798	109,668	7	45	7	54	106	0.993	0.997	0.988
KDM6A	chrX:44,732,423-44,971,845	237,859	29	338	138	62	538	0.942	0.982	0.731
MAP2K4	chr17:11,924,135-12,047,051	120,374	11	134	7	149	290	0.968	0.983	0.904
MAP3K1	chr5:56,110,900-56,191,978	78,107	20	60	28	204	292	0.951	0.985	0.933
MAP3K13	chr3:185,080,836-185,206,882	53,875	13	152	80	263	495	0.913	0.952	0.729
MEN1	chr11:64,570,986-64,578,188	5,776	9	276	2	96	374	0.916	0.948	0.909
MLH1	chr3:37,034,841-37,092,337	57,106	19	131	43	216	390	0.904	0.954	0.695
MSH2	chr2:47,630,263-47,710,360	79,578	16	285	105	169	559	0.856	0.943	0.537
NCOR1	chr17:15,933,408-16,118,874	162,274	45	376	141	208	725	0.905	0.962	0.510
NF1	chr17:29,421,945-29,704,695	278,846	58	327	172	208	707	0.929	0.977	0.592
NF2	chr22:29,999,545-30,094,589	90,804	16	324	86	246	656	0.894	0.952	0.685
PAX5	chr9:36,838,531-37,034,476	193,472	10	171	54	142	367	0.972	0.991	0.910
PBRM1	chr3:52,579,368-52,713,739	131,649	29	150	156	102	408	0.867	0.938	0.376
PRDM1	chr6:106,534,195-106,557,814	20,933	7	119	5	119	234	0.970	0.981	0.954
PTGFRN	chr1:117,452,689-117,532,972	76,764	9	118	18	106	242	0.967	0.986	0.930
RB1	chr13:48,877,883-49,056,026	176,159	27	145	57	72	274	0.922	0.989	0.774
SBDS	chr7:66,452,690-66,460,588	7,047	5	253	9	360	622	0.861	0.902	0.787
SDHD	chr11:111,957,571-111,966,518	8,063	4	195	8	157	360	0.910	0.950	0.888
SMARCB1	chr22:24,129,150-24,176,705	47,011	9	253	61	213	527	0.946	0.969	0.748
SMARCD1	chr12:50,478,983-50,494,494	13,631	13	170	13	373	556	0.874	0.950	0.854
SMAD4	chr18:48,556,583-48,611,411	31,421	11	201	32	154	387	0.905	0.947	0.881
SPRED1	chr15:38,545,052-38,649,450	98,479	7	81	29	67	177	0.974	0.986	0.938
STK11	chr19:1,205,798-1,228,434	19,734	9	261	17	215	493	0.918	0.956	0.874
SUFU	chr10:104,263,719-104,393,214	126,003	12	263	128	286	677	0.913	0.957	0.781
TBX3	chr12:115,108,059-115,121,969	11,360	7	232	0	182	414	0.967	0.969	0.963
TNFAIP3	chr6:138,188,581-138,204,449	10,092	8	82	2	82	166	0.977	0.985	0.969
TP53	chr17:7,571,720-7,590,863	6,986	10	193	33	313	539	0.873	0.895	0.791
TRIM36	chr5:114,460,459-114,516,243	53,535	10	46	22	51	119	0.969	0.988	0.912
TSC1	chr9:135,766,735-135,820,020	32,638	21	174	23	159	356	0.928	0.967	0.838
TSC2	chr16:2,097,990-2,138,713	39,995	41	275	25	218	518	0.910	0.972	0.749
VHL	chr3:10,183,319-10,195,354	8,118	3	292	21	213	526	0.871	0.853	0.812
