# Supplementary material for: A Comparison of 100 Human Genes Using an Alu Element-Based Instability Model
Source: PLoS One. 2013 Jun 3;8(6):e65188. doi: 10.1371/journal.pone.0065188 (PMC3670932; doi:10.1371/journal.pone.0065188)
Supplement: Table S4 — Characteristics of the 50 randomly chosen human genes examined in this study. (PDF) [file pone.0065188.s004.pdf]

Characteristics of 50 randomly chosen human genes

		Coding Region		*Alu* Population Across *Alu* Landscape				Raw Stability Scores		
		Length	Coding	250 kbp		250 kbp		Coding Exon Scores		Gene
Gene	Locus	(bp)	Exons	5' Flanking	Gene	3' Flanking	Total	Lowest	Highest	Score
ADGB	chr6:146,920,136-147,136,597	216,177	36	30	48	48	126	0.954	0.995	0.779
ARSH	chrX:2,924,654-2,951,426	26,773	9	239	28	158	425	0.884	0.935	0.726
BLK	chr8:11,351,521-11,422,108	20,884	12	80	24	101	205	0.969	0.987	0.950
C19orf76	chr19:50,191,942-50,194,247	656	2	361	0	300	661	0.925	0.928	0.925
CDH13	chr16:82,660,399-83,830,215	1,167,938	15	41	317	129	487	0.954	0.994	0.802
CHAMP1	chr13:115,079,965-115,092,803	2,439	1	95	9	6	110	only coding exon = 0.968		
CHRNA1	chr2:175,612,323-175,629,200	16,271	10	99	9	120	228	0.949	0.979	0.911
CHST9	chr18:24,495,595-24,765,289	226,551	5	60	47	82	189	0.979	.994	0.959
CYB5B	chr16:69,458,498-69,500,167	37,835	3	350	38	285	673	0.906	0.934	0.828
DCAF6	chr1:167,905,797-168,045,083	138,524	19	201	67	147	415	0.930	0.978	0.773
DTNBP1	chr6:15,523,032-15,663,289	139,895	10	158	16	129	303	0.943	0.983	0.841
GDPD2	chrX:69,642,881-69,653,241	8,102	16	142	2	185	329	0.072	0.966	0.071
GFI1	chr1:92,940,318-92,951,628	7,459	6	130	3	117	250	0.946	0.967	0.938
GRPEL2	chr5:148,724,977-148,734,146	5,743	4	59	6	236	301	0.928	0.964	0.913
H2AFB3	chrX:154,113,317-154,113,833	348	1	92	0	76	168	only coding exon = 0.986		
HDGFL1	chr6:22,569,678-22,570,750	756	1	60	0	39	99	only coding exon = 0.995		
HSPB9	chr17:40,274,756-40,275,371	480	1	289	0	285	574	only coding exon = 0.911		
IL17D	chr13:21,277,482-21,297,237	17,953	2	194	9	257	460	0.955	0.961	0.940
JMY	chr5:78,531,925-78,623,038	79,657	10	212	92	189	493	0.896	0.949	0.726
KCNA6	chr12:4,918,342-4,960,278	1,590	1	81	9	43	133	only coding exon = 0.991		
KEAP1	chr19:10,596,796-10,614,054	13,382	5	493	43	454	990	0.784	0.830	0.621
KIAA1598	chr10:118,644,306-118,765,088	118,736	15	140	44	96	280	0.946	0.986	0.821
MADCAM1	chr19:496,490-505,343	8,466	5	163	11	268	442	0.897	0.940	0.861
MAP9	chr4:156,263,812-156,298,122	28,068	13	81	6	34	121	0.972	0.989	0.934
MFSD4	chr1:205,538,112-205,572,046	31,392	10	153	14	192	359	0.943	0.969	0.882
MIA3	chr1:222,791,444-222,841,351	47,509	28	132	16	126	274	0.960	0.980	0.885
MRPS9	chr2:105,654,483-105,716,418	61,667	11	64	17	91	172	0.976	0.989	0.942
MUT	chr6:49,398,073-49,431,041	27,739	12	100	8	41	149	0.969	0.990	0.951
NANOS3	chr19:13,988,063-13,991,571	3,255	2	429	6	393	828	0.872	0.896	0.858
NCF1	chr7:74,188,309-74,203,659	15,126	11	432	21	466	919	0.876	0.893	0.723
NGB	chr14:77,731,834-77,737,655	4,402	4	215	1	152	368	0.974	0.978	0.970
OPRD1	chr1:29,138,654-29,190,208	50,900	3	473	72	301	846	0.898	0.928	0.844
OR6P1	chr1:158,532,441-158,533,394	954	1	36	0	42	78	only coding exon = 0.993		
PACSIN1	chr6:34,482,649-34,504,039	6,225	9	323	0	320	643	0.950	0.951	0.973
PATE4	chr11:125,703,211-125,709,967	5,068	3	95	0	110	205	0.980	0.988	0.977
PHKA2	chrX:18,910,416-19,002,480	90,448	33	147	47	187	381	0.898	0.971	0.724
PSG2	chr19:43,568,362-43,586,893	16,013	5	47	3	71	121	0.971	0.990	0.961
SET	chr9:131,451,509-131,458,675	10,769	8	403	2	377	782	0.872	0.907	0.821
SF3B3	chr16:70,557,691-70,611,571	45,157	25	447	64	168	679	0.840	0.926	0.499
SFRP5	chr10:99,526,508-99,531,756	4,320	3	80	1	257	338	0.960	0.965	0.952
SPATA7	chr14:88,851,742-88,904,804	52,604	12	75	20	164	259	0.968	0.980	0.924
TAGLN2	chr1:159,887,903-159,895,284	1,710	4	80	0	58	138	0.984	0.987	0.984
THYN1	chr11:134,118,173-134,123,260	4,445	7	62	0	108	170	0.906	0.913	0.978
TMEM136	chr11:120,195,838-120,204,388	3,140	3	73	2	129	204	0.975	0.979	0.973
TRNP1	chr1:27,320,195-27,327,377	684	1	317	4	334	655	only coding exon = 0.917		
TUBA1C	chr12:49,658,865-49,667,113	8,046	4	333	9	307	649	0.873	0.899	0.817
USMG5	chr10:105,148,809-105,156,270	264	2	168	8	376	552	0.893	0.902	0.892
XPNPEP3	chr22:41,253,085-41,328,823	69,254	10	332	109	416	857	0.859	0.926	0.563
ZNF296	chr19:45,574,758-45,579,688	4,773	3	333	6	407	746	0.896	0.917	0.885
ZNF567	chr19:37,180,303-37,212,225	8,199	3	282	29	270	581	0.888	0.917	0.853
