# Supplementary material for: A Comparison of 100 Human Genes Using an Alu Element-Based Instability Model
Source: PLoS One. 2013 Jun 3;8(6):e65188. doi: 10.1371/journal.pone.0065188 (PMC3670932; doi:10.1371/journal.pone.0065188)
Supplement: Table S5 — Spacer size percentile samples sizes versus APSN for Type 1 Alu pairs. (PDF) [file pone.0065188.s005.pdf]

Table S5

Spacer size percentile samples sizes versus APSN for Type 1 *Alu* pairs

APSN	Percentile[1]									
	2.5th	10th	20th	30th	40th	50th	60th	70th	80th	90th
1	2,611	5,263	5,307	5,263	5,317	5,287	5,255	5,316	5,274	5,299
2	4,643	9,297	9,406	9,358	9,410	9,340	9,360	9,365	9,358	9,375
3	5,475	11,050	11,130	11,047	11,060	11,108	11,051	11,068	11,076	11,061
4	5,765	11,675	11,681	11,704	11,716	11,720	11,690	11,713	11,694	11,685
5	5,952	12,026	12,028	12,029	12,060	12,003	12,082	12,048	12,039	12,042
6	6,027	12,216	12,198	12,223	12,229	12,272	12,140	12,289	12,210	12,186
7	6,102	12,244	12,284	12,240	12,291	12,276	12,306	12,260	12,294	12,271
8	6,084	12,383	12,344	12,342	12,391	12,322	12,363	12,340	12,333	12,372
9	6,139	12,425	12,430	12,444	12,382	12,434	12,388	12,445	12,422	12,436
10	6,112	12,435	12,455	12,422	12,417	12,435	12,407	12,428	12,403	12,440
11	6,163	12,379	12,401	12,485	12,434	12,372	12,455	12,418	12,465	12,371
12	6,195	12,479	12,451	12,460	12,488	12,471	12,520	12,467	12,463	12,496
13	6,141	12,463	12,417	12,409	12,487	12,432	12,439	12,458	12,423	12,455
14	6,141	12,424	12,448	12,453	12,448	12,411	12,390	12,464	12,429	12,453
15	6,164	12,400	12,440	12,412	12,417	12,461	12,420	12,463	12,408	12,427
16	6,108	12,467	12,422	12,488	12,444	12,446	12,491	12,450	12,444	12,479
17	6,121	12,512	12,423	12,463	12,498	12,413	12,475	12,471	12,481	12,462
18	6,107	12,470	12,400	12,446	12,429	12,458	12,392	12,481	12,408	12,434
19	6,131	12,399	12,415	12,414	12,405	12,424	12,357	12,413	12,470	12,387
20	6,176	12,417	12,438	12,478	12,481	12,413	12,477	12,433	12,463	12,470
21	6,150	12,415	12,443	12,413	12,400	12,472	12,441	12,409	12,451	12,423
22	6,095	12,404	12,394	12,431	12,320	12,430	12,357	12,415	12,427	12,351
23	6,113	12,411	12,386	12,425	12,357	12,448	12,360	12,415	12,428	12,391

APSN	Percentile(1)									
	**2.5th**	**10th**	**20th**	**30th**	**40th**	**50th**	**60th**	**70th**	**80th**	**90th**
24	6,150	12,411	12,460	12,422	12,429	12,434	12,398	12,479	12,412	12,479
25	6,120	12,418	12,415	12,363	12,373	12,473	12,359	12,415	12,390	12,438
26	6,115	12,406	12,377	12,383	12,403	12,370	12,406	12,365	12,421	12,360
27	6,142	12,361	12,425	12,388	12,449	12,403	12,383	12,438	12,420	12,361
28	6,103	12,419	12,412	12,417	12,415	12,405	12,400	12,402	12,400	12,421
29	6,114	12,347	12,394	12,401	12,386	12,400	12,363	12,345	12,389	12,401
30	6,126	12,356	12,354	12,332	12,359	12,354	12,392	12,302	12,414	12,331
31	6,123	12,368	12,395	12,340	12,425	12,401	12,363	12,387	12,392	12,399
32	6,089	12,364	12,402	12,353	12,376	12,379	12,410	12,353	12,348	12,385
33	6,049	12,470	12,428	12,382	12,354	12,435	12,399	12,394	12,395	12,408
34	6,080	12,427	12,406	12,390	12,352	12,435	12,381	12,445	12,365	12,427
35	6,099	12,336	12,358	12,377	12,366	12,348	12,353	12,337	12,370	12,315
36	6,137	12,315	12,438	12,396	12,349	12,346	12,430	12,374	12,392	12,396
37	6,101	12,393	12,370	12,373	12,385	12,378	12,394	12,395	12,385	12,360
38	6,076	12,398	12,357	12,370	12,396	12,376	12,346	12,371	12,367	12,379
39	6,114	12,374	12,327	12,408	12,333	12,403	12,343	12,413	12,365	12,357
40	6,091	12,362	12,382	12,374	12,369	12,370	12,366	12,359	12,403	12,364
41	6,102	12,398	12,382	12,410	12,391	12,408	12,394	12,393	12,373	12,416
42	6,135	12,407	12,409	12,440	12,450	12,402	12,416	12,426	12,445	12,430
43	6,150	12,411	12,425	12,415	12,393	12,436	12,431	12,416	12,423	12,389
44	6,086	12,422	12,427	12,407	12,384	12,418	12,342	12,456	12,399	12,382
45	6,076	12,419	12,403	12,330	12,393	12,376	12,398	12,383	12,408	12,375
46	6,103	12,355	12,356	12,372	12,312	12,349	12,361	12,367	12,345	12,386
47	6,101	12,393	12,404	12,373	12,354	12,397	12,376	12,396	12,408	12,382
48	6,113	12,381	12,399	12,349	12,399	12,351	12,396	12,422	12,362	12,401
49	6,147	12,379	12,448	12,406	12,417	12,441	12,425	12,438	12,422	12,415
50	6,104	12,358	12,349	12,358	12,349	12,351	12,362	12,381	12,350	12,352

APSN	Percentile(1)									
	2.5th	10th	20th	30th	40th	50th	60th	70th	80th	90th
51	6,107	12,389	12,391	12,332	12,353	12,380	12,385	12,373	12,373	12,386
52	6,081	12,380	12,390	12,402	12,365	12,369	12,353	12,416	12,351	12,391
53	6,083	12,341	12,349	12,321	12,333	12,399	12,343	12,335	12,329	12,333
54	6,127	12,312	12,398	12,401	12,329	12,382	12,394	12,371	12,390	12,403
55	6,135	12,341	12,444	12,342	12,404	12,400	12,347	12,393	12,426	12,399
56	6,070	12,293	12,370	12,322	12,298	12,340	12,330	12,312	12,365	12,331
57	6,098	12,337	12,399	12,325	12,365	12,383	12,376	12,343	12,371	12,373
58	6,094	12,331	12,362	12,372	12,328	12,386	12,320	12,345	12,383	12,337
59	6,086	12,365	12,337	12,310	12,355	12,329	12,404	12,293	12,388	12,333
60	6,098	12,323	12,317	12,336	12,382	12,295	12,343	12,327	12,334	12,356
61	6,037	12,372	12,369	12,315	12,312	12,289	12,361	12,365	12,308	12,329
62	6,085	12,303	12,272	12,355	12,260	12,281	12,323	12,300	12,316	12,328
63	6,089	12,319	12,341	12,330	12,317	12,325	12,356	12,323	12,358	12,295
64	6,069	12,276	12,330	12,328	12,296	12,308	12,326	12,318	12,288	12,322
65	6,038	12,405	12,342	12,379	12,332	12,322	12,357	12,379	12,378	12,333
66	6,093	12,328	12,404	12,316	12,369	12,351	12,356	12,342	12,378	12,333
67	6,052	12,416	12,350	12,365	12,355	12,332	12,369	12,366	12,400	12,336
68	6,093	12,340	12,312	12,299	12,318	12,395	12,306	12,306	12,360	12,313
69	6,081	12,348	12,349	12,386	12,369	12,315	12,379	12,344	12,382	12,348
70	6,120	12,312	12,356	12,345	12,339	12,323	12,348	12,354	12,323	12,344
71	6,045	12,310	12,314	12,321	12,340	12,285	12,316	12,340	12,296	12,331
72	6,066	12,385	12,305	12,337	12,358	12,288	12,371	12,343	12,314	12,356
73	6,110	12,335	12,383	12,301	12,408	12,350	12,328	12,375	12,383	12,339
74	6,022	12,334	12,342	12,323	12,271	12,301	12,346	12,265	12,362	12,295
75	6,035	12,322	12,293	12,282	12,275	12,276	12,318	12,288	12,284	12,319
76	6,066	12,273	12,290	12,303	12,268	12,302	12,235	12,319	12,298	12,288
77	6,096	12,244	12,356	12,288	12,301	12,302	12,304	12,338	12,325	12,294

APSN	Percentile(1)									
	2.5th	10th	20th	30th	40th	50th	60th	70th	80th	90th
78	6,077	12,313	12,383	12,296	12,354	12,340	12,310	12,357	12,332	12,310
79	6,117	12,340	12,394	12,337	12,385	12,387	12,388	12,354	12,352	12,364
80	6,101	12,339	12,327	12,343	12,343	12,324	12,353	12,337	12,347	12,347
81	6,081	12,309	12,291	12,315	12,293	12,354	12,264	12,319	12,330	12,298
82	6,055	12,316	12,346	12,300	12,298	12,319	12,344	12,292	12,307	12,336
83	6,069	12,363	12,308	12,330	12,345	12,296	12,366	12,346	12,343	12,291
84	6,086	12,319	12,342	12,354	12,333	12,366	12,305	12,383	12,326	12,343
85	6,067	12,294	12,349	12,312	12,305	12,332	12,309	12,322	12,327	12,298
86	6,069	12,306	12,292	12,262	12,308	12,275	12,281	12,289	12,329	12,300
87	6,071	12,311	12,311	12,298	12,363	12,308	12,281	12,312	12,350	12,276
88	6,031	12,321	12,333	12,274	12,328	12,260	12,291	12,353	12,320	12,283
89	6,091	12,326	12,286	12,307	12,327	12,302	12,358	12,310	12,307	12,338
90	6,013	12,411	12,273	12,308	12,307	12,347	12,290	12,310	12,336	12,325
91	6,052	12,312	12,278	12,279	12,300	12,256	12,312	12,287	12,295	12,295
92	6,081	12,283	12,310	12,295	12,307	12,315	12,313	12,309	12,338	12,289
93	6,027	12,312	12,308	12,292	12,292	12,246	12,308	12,252	12,311	12,311
94	6,037	12,300	12,253	12,244	12,244	12,304	12,236	12,281	12,258	12,303
95	6,051	12,336	12,307	12,287	12,296	12,266	12,302	12,340	12,323	12,295
96	6,072	12,271	12,263	12,258	12,301	12,300	12,256	12,284	12,277	12,327
97	6,040	12,338	12,369	12,270	12,351	12,334	12,328	12,305	12,359	12,316
98	6,044	12,332	12,320	12,320	12,322	12,334	12,313	12,315	12,345	12,298
99	6,041	12,394	12,279	12,355	12,300	12,347	12,287	12,364	12,306	12,341
100	6,080	12,219	12,325	12,258	12,261	12,312	12,266	12,280	12,291	12,274
101	6,047	12,300	12,306	12,249	12,318	12,242	12,319	12,263	12,336	12,274
102	6,060	12,242	12,263	12,232	12,289	12,220	12,260	12,260	12,280	12,269
103	6,048	12,277	12,270	12,286	12,240	12,309	12,236	12,324	12,263	12,249
104	6,042	12,219	12,269	12,212	12,245	12,283	12,221	12,245	12,278	12,239

APSN	Percentile[1]									
	**2.5th**	**10th**	**20th**	**30th**	**40th**	**50th**	**60th**	**70th**	**80th**	**90th**
105	6,023	12,307	12,244	12,256	12,260	12,290	12,252	12,248	12,265	12,267
106	6,024	12,346	12,315	12,310	12,319	12,276	12,304	12,355	12,305	12,312
107	6,066	12,223	12,290	12,258	12,260	12,276	12,209	12,324	12,264	12,276
108	6,049	12,323	12,305	12,294	12,304	12,278	12,281	12,296	12,346	12,292
109	6,055	12,257	12,339	12,262	12,288	12,272	12,269	12,323	12,264	12,319
110	6,065	12,207	12,264	12,222	12,246	12,281	12,213	12,251	12,241	12,226
111	6,020	12,286	12,273	12,258	12,247	12,271	12,252	12,241	12,248	12,308
112	6,042	12,264	12,237	12,254	12,264	12,281	12,239	12,260	12,273	12,254
113	6,039	12,275	12,296	12,245	12,222	12,304	12,249	12,300	12,262	12,254
114	6,019	12,296	12,228	12,263	12,245	12,263	12,227	12,274	12,272	12,252
115	6,041	12,318	12,325	12,258	12,335	12,270	12,311	12,312	12,302	12,306

(1) The percentile groupings for this table are as follows.

Percentile Name	Lower Limit	Percentile Midpoint	Upper Limit
2.5th	0th	2.5th	5th
10th	5th	10th	15th
20th	15th	20th	15th
30th	25th	30th	35th
40th	35th	40th	45th
50th	45th	50th	55th
60th	55th	60th	65th
70th	65th	70th	75th
80th	75th	80th	85th
90th	85th	90th	95th
