# Supplementary material for: A Comparison of 100 Human Genes Using an Alu Element-Based Instability Model
Source: PLoS One. 2013 Jun 3;8(6):e65188. doi: 10.1371/journal.pone.0065188 (PMC3670932; doi:10.1371/journal.pone.0065188)
Supplement: Table S6 — Coefficients for equations describing the I∶D ratio versus spacer size for Type 1 Alu pairs. (PDF) [file pone.0065188.s006.pdf]

Table S6

Coefficients for equations describing the I:D ratio versus spacer size for Type 1 *Alu* pairs

APSN	Spacer Size Range, bp			Equation Type	Equation Coefficients			
	Range ID	Range Start (bp)	Range End (bp)		C1	C2	C3	C4
1	A	51	99	Constant	0.79941			
	B	100	264	Line (C1= m and C2= b)	0.9194	0.0007272379		
	C	265	469	Line (C1= m and C2= b)	0.91287	-0.0000318732		
	D	470	690	Line (C1= m and C2= b)	0.91759	0.0000213801		
	E	691	7,538	Log10-Log10 Cubic	7.78075	-6.7456467666	1.91217	-0.1783
	F	7,539	21,717	Line (C1= m and C2= b)	0.95928	0.0000028717		
2	A	51	343	Constant	0.93436			
	B	344	549	Line (C1= m and C2= b)	0.9772	0.0002079614		
	C	550	10,299	Log10-Log10 Cubic	2.32578	-1.7777937965	0.42427	-0.0316
	D	10,300	29,292	Line (C1= m and C2= b)	0.96048	0.0000020805		
3	A	51	631	Constant	0.97516			
	B	632	14,072	Log10-Log10 Cubic	0.64634	-0.3094873157	0.00741	0.00689
	C	14,071	37,466	Line (C1= m and C2= b)	0.95926	0.0000017414		
4	A	51	971	Constant	0.97879			
	B	972	18,160	Log10-Log10 Cubic	3.30368	-2.4948824775	0.60811	-0.0483
	C	18,161	46,577	Line (C1= m and C2= b)	0.95435	0.0000016063		
5	A	51	1,323	Constant	0.97511			
	C	1,324	22,676	Log10-Log10 Cubic	5.31725	-4.0530801705	1.01082	-0.083
	D	22,677	55,728	Line (C1= m and C2= b)	0.95187	0.0000014562		
6	A	51	1,693	Constant	0.96868			
	B	1,694	26,666	Log10-Log10 Cubic	6.78571	-5.1303849864	1.27409	-0.1045
	C	26,667	63,564	Line (C1= m and C2= b)	0.95072	0.0000013357		
7	A	51	2,090	Constant	0.96075			
	B	2,091	8,100	Log10-Log10 Cubic	7.67666	-5.7385946167	1.41142	-0.1147
	C	8,101	31,551	Log10-Log10 Quadratic	0.95541	-0.5177208724	0.06707	
	D	31,552	72,428	Line (C1= m and C2= b)	0.96339	0.0000008957		
8	A	51	2,499	Constant	0.95258			
	B	2,500	9,500	Log10-Log10 Cubic	8.03752	-5.9433109647	1.44697	-0.1165

	C	9,501	36,034	Log10-Log10 Quadratic	0.84396	-0.4550433066	0.05843	
	D	36,035	80,576	Line (C1= m and C2= b)	0.96309	0.0000008286		
**9**	A	51	2,911	Constant	0.94465			
	B	2,912	10,750	Log10-Log10 Cubic	7.87334	-5.7646659812	1.38977	-0.1108
	C	10,751	40,625	Log10-Log10 Quadratic	0.71591	-0.3868429278	0.04945	
	D	40,626	89,224	Line (C1= m and C2= b)	0.9627	0.0000007675		
**10**	A	51	3,336	Constant	0.93683			
	B	3,337	12,100	Log10-Log10 Cubic	7.3152	-5.3114112439	1.26928	-0.1003
	C	12,101	45,150	Log10-Log10 Quadratic	0.5754	-0.3149352684	0.04033	
	D	45,151	97,408	Line (C1= m and C2= b)	0.96219	0.0000007235		
**11**	A	51	3,765	Constant	0.92948			
	B	3,766	13,400	Log10-Log10 Cubic	6.33025	-4.5652093163	1.08231	-0.0848
	C	13,401	49,917	Log10-Log10 Quadratic	0.4264	-0.2407097699	0.03115	
	D	49,918	106,671	Line (C1= m and C2= b)	0.96162	0.0000006763		
**12**	A	51	4,218	Constant	0.92198			
	B	4,219	14,800	Log10-Log10 Cubic	4.98994	-3.5857176421	0.84491	-0.0657
	C	14,801	54,434	Log10-Log10 Quadratic	0.26715	-0.1634456012	0.02183	
	D	54,435	114,298	Line (C1= m and C2= b)	0.96084	0.0000006541		
**13**	A	51	4,671	Constant	0.92562			
	B	4,672	12,900	Log10-Log10 Cubic	5.62479	-4.0015748693	0.93521	-0.0722
	C	12,901	58,961	Log10-Log10 Quadratic	0.2831	-0.1684096940	0.02212	
	D	58,962	122,454	Line (C1= m and C2= b)	0.96183	0.0000006011		
**14**	A	51	5,133	Constant	0.92835			
	B	5,134	13,800	Log10-Log10 Cubic	5.95354	-4.1982490417	0.97354	-0.0746
	C	13,801	63,302	Log10-Log10 Quadratic	0.28973	-0.1691164402	0.02194	
	D	63,303	130,613	Line (C1= m and C2= b)	0.96256	0.0000005562		
**15**	A	51	5,573	Constant	0.93092			
	B	5,574	15,000	Log10-Log10 Cubic	6.01221	-4.2039766602	0.96714	-0.0736
	C	15,001	68,492	Log10-Log10 Quadratic	0.2881	-0.1658382799	0.02128	
	D	68,493	139,999	Line (C1= m and C2= b)	0.96336	0.0000005562		
**16**	A	51	6,040	Constant	0.93299			
	B	6,041	20,000	Log10-Log10 Cubic	6.03715	-4.1900057268	0.95715	-0.0723
	C	20,001	73,690	Log10-Log10 Quadratic	0.2771	-0.1584836840	0.02019	
	D	73,691	148,620	Line (C1= m and C2= b)	0.96404	0.0000004799		
**17**	A	51	6,505	Constant	0.93515			
	B	6,506	21,000	Log10-Log10 Cubic	6.3595	-4.3846910584	0.9958	-0.0748
	C	21,001	77,558	Log10-Log10 Quadratic	0.28357	-0.1597125440	0.02015	
	D	77,559	155,268	Line (C1= m and C2= b)	0.96466	0.0000004548		

**18**	A	51	6,979	Constant	0.93723			
	B	6,980	22,700	Log10-Log10 Cubic	6.60112	-4.5212397393	1.02062	-0.0762
	C	22,701	82,510	Log10-Log10 Quadratic	0.28491	-0.1585531188	0.01983	
	D	82,511	163,997	Line (C1= m and C2= b)	0.96547	0.0000004238		
**19**	A	51	7,462	Constant	0.93911			
	B	7,463	23,900	Log10-Log10 Cubic	6.86596	-4.6745775179	1.04952	-0.078
	C	23,901	86,868	Log10-Log10 Quadratic	0.28819	-0.1584532865	0.01965	
	D	86,869	170,083	Line (C1= m and C2= b)	0.96613	0.0000004070		
**20**	A	51	7,959	Constant	0.94104			
	B	7,960	25,500	Log10-Log10 Cubic	7.08419	-4.7943952397	1.07051	-0.0791
	C	25,501	91,524	Log10-Log10 Quadratic	0.292	-0.1585728258	0.0195	
	D	91,525	177,631	Line (C1= m and C2= b)	0.96686	0.0000003849		
**21**	A	51	8,411	Constant	0.94274			
	B	8,412	26,750	Log10-Log10 Cubic	7.22215	-4.8615843620	1.08005	-0.0795
	C	26,751	96,502	Log10-Log10 Quadratic	0.29317	-0.1576419079	0.01925	
	D	96,503	186,522	Line (C1= m and C2= b)	0.9676	0.0000003599		
**22**	A	51	8,889	Constant	0.9445			
	B	8,890	28,100	Log10-Log10 Cubic	7.4093	-4.9616460858	1.09696	-0.0803
	C	28,101	101,169	Log10-Log10 Quadratic	0.29772	-0.1582725229	0.01918	
	D	101,170	194,649	Line (C1= m and C2= b)	0.96831	0.0000003390		
**23**	A	51	9,389	Constant	0.94623			
	B	9,390	29,500	Log10-Log10 Cubic	7.57276	-5.0453328033	1.11018	-0.0809
	C	29,501	105,759	Log10-Log10 Quadratic	0.30285	-0.1591522224	0.01914	
	D	105,760	201,592	Line (C1= m and C2= b)	0.96895	0.0000003240		
**24**	A	51	9,852	Constant	9853			
	B	9,853	31,000	Log10-Log10 Cubic	7.68002	-5.0928725095	1.1157	-0.081
	C	31,001	110,468	Log10-Log10 Quadratic	0.307	-0.1596808311	0.01907	
	D	110,469	207,881	Line (C1= m and C2= b)	0.96962	0.0000003118		
**25**	A	51	10,342	Constant	0.94941			
	B	10,343	32,300	Log10-Log10 Cubic	7.80893	-5.1544774357	1.12428	-0.0813
	C	32,301	115,375	Log10-Log10 Quadratic	0.31133	-0.1603210874	0.01901	
	D	115,376	217,907	Line (C1= m and C2= b)	0.97031	0.0000002896		
**26**	A	51	10,835	Constant	0.95089			
	B	10,836	33,500	Log10-Log10 Cubic	8.00558	-5.2620537953	1.14327	-0.0823
	C	33,501	119,496	Log10-Log10 Quadratic	0.31836	-0.1622678128	0.01911	
	D	119,497	225,030	Line (C1= m and C2= b)	0.97087	0.0000002760		
**27**	A	51	11,318	Constant	0.95243			
	B	11,319	35,000	Log10-Log10 Cubic	8.06202	-5.2767565134	1.14185	-0.0819

	C	35,001	124,295	Log10-Log10 Quadratic	0.32327	-0.1631763677	0.01909	
	D	124,296	233,993	Line (C1= m and C2= b)	0.97154	0.0000002595		
**28**	A	51	11,852	Constant	0.95393			
	B	11,853	36,300	Log10-Log10 Cubic	8.22296	-5.3592619123	1.15509	-0.0825
	C	36,301	128,840	Log10-Log10 Quadratic	0.33068	-0.1652284960	0.0192	
	D	128,841	241,529	Line (C1= m and C2= b)	0.97212	0.0000002474		
**29**	A	51	12,319	Constant	0.95524			
	B	12,320	37,700	Log10-Log10 Cubic	8.24358	-5.3522305553	1.14935	-0.0818
	C	37,701	133,782	Log10-Log10 Quadratic	0.33379	-0.1654797210	0.01912	
	D	133,783	249,163	Line (C1= m and C2= b)	0.97275	0.0000002362		
**30**	A	51	12,811	Constant	0.95663			
	B	12,812	39,000	Log10-Log10 Cubic	8.35346	-5.4032014313	1.15617	-0.082
	C	39,001	138,266	Log10-Log10 Quadratic	0.3413	-0.1676708908	0.01926	
	D	138,267	239,318	Line (C1= m and C2= b)	0.97333	0.0000002639		
**31**	A	51	13,375	Constant	0.958			
	B	13,376	40,500	Log10-Log10 Cubic	8.52009	-5.4904964718	1.17077	-0.0828
	C	40,501	142,612	Log10-Log10 Quadratic	0.34866	-0.1697504644	0.01938	
	D	142,613	265,420	Line (C1= m and C2= b)	0.97388	0.0000002127		
**32**	A	51	13,838	Constant	0.95917			
	B	13,839	41,500	Log10-Log10 Cubic	8.48164	-5.4468271723	1.15756	-0.0816
	C	41,501	147,465	Log10-Log10 Quadratic	0.35274	-0.1704882715	0.01936	
	D	147,466	271,220	Line (C1= m and C2= b)	0.97444	0.0000002066		
**33**	A	51	14,385	Constant	0.9605			
	B	14,386	43,200	Log10-Log10 Cubic	8.60465	-5.5059347391	1.16614	-0.0819
	C	43,201	152,367	Log10-Log10 Quadratic	0.35969	-0.1724239548	0.01947	
	D	152,368	279,208	Line (C1= m and C2= b)	0.97503	0.0000001969		
**34**	A	51	14,914	Constant	0.96179			
	B	14,915	44,600	Log10-Log10 Cubic	8.64496	-5.5117456963	1.16331	-0.0815
	C	44,601	157,912	Log10-Log10 Quadratic	0.36526	-0.1737900390	0.01952	
	D	157,913	287,054	Line (C1= m and C2= b)	0.97572	0.0000001880		
**35**	A	51	15,380	Constant	0.96293			
	B	15,381	45,800	Log10-Log10 Cubic	8.75243	-5.5650925696	1.17158	-0.0818
	C	45,801	160,843	Log10-Log10 Quadratic	0.37522	-0.1771714507	0.0198	
	D	160,844	294,503	Line (C1= m and C2= b)	0.97604	0.0000001792		
**36**	A	51	15,961	Constant	0.96414			
	B	15,962	47,300	Log10-Log10 Cubic	8.80507	-5.5789392078	1.17055	-0.0815
	C	47,301	166,334	Log10-Log10 Quadratic	0.38036	-0.1783330903	0.01983	
	D	166,335	303,528	Line (C1= m and C2= b)	0.97666	0.0000001701		

**37**	A	51	16,384	Constant	0.96533			
	B	16,385	49,000	Log10-Log10 Cubic	8.72895	-5.5142209618	1.1536	-0.0801
	C	49,001	171,223	Log10-Log10 Quadratic	0.38752	-0.1804542509	0.01997	
	D	171,224	310,150	Line (C1= m and C2= b)	0.97723	0.0000001639		
**38**	A	51	16,899	Constant	0.96641			
	B	16,900	50,000	Log10-Log10 Cubic	8.74565	-5.5081973951	1.149	-0.0795
	C	50,001	176,317	Log10-Log10 Quadratic	0.39136	-0.1811777640	0.01996	
	D	176,318	318,342	Line (C1= m and C2= b)	0.9778	0.0000001563		
**39**	A	51	17,438	Constant	0.96756			
	B	17,439	51,500	Log10-Log10 Cubic	8.80864	-5.5309815528	1.1504	-0.0794
	C	51,501	181,108	Log10-Log10 Quadratic	0.40054	-0.1841547520	0.02019	
	D	181,109	327,005	Line (C1= m and C2= b)	0.97832	0.0000001486		
**40**	A	51	17,946	Constant	0.96871			
	B	17,947	52,750	Log10-Log10 Cubic	8.74909	-5.4776029778	1.13609	-0.0782
	C	52,751	185,418	Log10-Log10 Quadratic	0.40868	-0.1866418148	0.02037	
	D	185,419	301,516	Line (C1= m and C2= b)	0.97877	0.0000001828		
**41**	A	51	18,399	Constant	0.96973			
	B	18,400	54,000	Log10-Log10 Cubic	8.79886	-5.4949973504	1.13697	-0.0781
	C	54,001	189,553	Log10-Log10 Quadratic	0.41743	-0.1895402963	0.0206	
	D	189,554	339,349	Line (C1= m and C2= b)	0.97922	0.0000001387		
**42**	A	51	18,988	Constant	0.97085			
	B	18,989	55,500	Log10-Log10 Cubic	8.82416	-5.4939691984	1.13342	-0.0776
	C	55,501	195,023	Log10-Log10 Quadratic	0.42413	-0.1914173967	0.02071	
	D	195,024	348,417	Line (C1= m and C2= b)	0.97978	0.0000001318		
**43**	A	51	19,504	Constant	0.97179			
	B	19,505	57,000	Log10-Log10 Cubic	8.73067	-5.4205615811	1.11521	-0.0762
	C	57,001	200,344	Log10-Log10 Quadratic	0.42797	-0.1921694132	0.02071	
	D	200,345	356,317	Line (C1= m and C2= b)	0.98031	0.0000001263		
**44**	A	51	20,051	Constant	0.97288			
	B	20,052	58,300	Log10-Log10 Cubic	8.79712	-5.4469249713	1.11771	-0.0761
	C	58,301	205,283	Log10-Log10 Quadratic	0.43743	-0.1952772019	0.02096	
	D	205,284	362,245	Line (C1= m and C2= b)	0.98082	0.0000001222		
**45**	A	51	20,583	Constant	0.97391			
	B	20,584	59,700	Log10-Log10 Cubic	8.76577	-5.4128755524	1.10782	-0.0753
	C	59,701	209,826	Log10-Log10 Quadratic	0.44597	-0.1979802132	0.02116	
	D	209,827	370,629	Line (C1= m and C2= b)	0.98123	0.0000001167		
**46**	A	51	21,074	Constant	0.9749			
	B	21,075	61,300	Log10-Log10 Cubic	8.71724	-5.3710822314	1.09697	-0.0744

	C	61,301	213,098	Log10-Log10 Quadratic	0.4517	-0.1995403580	0.02125	
	D	213,099	378,013	Line (C1= m and C2= b)	0.98157	0.0000001118		
**47**	A	51	21,635	Constant	0.97582			
	B	21,636	62,600	Log10-Log10 Cubic	8.71833	-5.3574844497	1.09137	-0.0738
	C	62,601	218,419	Log10-Log10 Quadratic	0.4586	-0.2016072791	0.0214	
	D	218,420	384,952	Line (C1= m and C2= b)	0.98206	0.0000001077		
**48**	A	51	22,155	Constant	0.97689			
	B	22,156	64,000	Log10-Log10 Cubic	8.68622	-5.3238643715	1.08179	-0.073
	C	64,001	223,001	Log10-Log10 Quadratic	0.4686	-0.2049163494	0.02166	
	D	223,002	392,076	Line (C1= m and C2= b)	0.98249	0.0000001035		
**49**	A	51	22,655	Constant	0.97772			
	B	22,656	65,300	Log10-Log10 Cubic	8.61097	-5.2653979547	1.06746	-0.0719
	C	65,301	227,791	Log10-Log10 Quadratic	0.47297	-0.2059762289	0.02171	
	D	227,792	399,869	Line (C1= m and C2= b)	0.98292	0.0000000993		
**50**	A	51	23,144	Constant	0.97874			
	B	23,145	66,700	Log10-Log10 Cubic	8.51836	-5.1965486331	1.05112	-0.0706
	C	66,701	231,295	Log10-Log10 Quadratic	0.48343	-0.2094683826	0.02199	
	D	231,296	407,231	Line (C1= m and C2= b)	0.98324	0.0000000952		
**51**	A	51	23,714	Constant	0.97959			
	B	23,715	68,100	Log10-Log10 Cubic	8.58137	-5.2228939903	1.05409	-0.0706
	C	68,101	236,850	Log10-Log10 Quadratic	0.48924	-0.2111573475	0.02211	
	D	236,851	415,131	Line (C1= m and C2= b)	0.9838	0.0000000909		
**52**	A	51	24,243	Constant	0.98053			
	B	24,244	69,600	Log10-Log10 Cubic	8.56614	-5.2022787638	1.04775	-0.0701
	C	69,601	240,314	Log10-Log10 Quadratic	0.49771	-0.2138742516	0.02232	
	D	240,315	422,838	Line (C1= m and C2= b)	0.9841	0.0000000871		
**53**	A	51	24,700	Constant	0.98118			
	B	24,701	71,100	Log10-Log10 Cubic	8.41163	-5.0969237864	1.02421	-0.0684
	C	71,101	246,671	Log10-Log10 Quadratic	0.4961	-0.2125883238	0.02213	
	D	246,672	428,614	Line (C1= m and C2= b)	0.98467	0.0000000843		
**54**	A	51	25,256	Constant	0.98196			
	B	25,257	72,500	Log10-Log10 Cubic	8.43964	-5.1044200744	1.02392	-0.0682
	C	72,501	249,886	Log10-Log10 Quadratic	0.50133	-0.2140452025	0.02222	
	D	249,887	437,018	Line (C1= m and C2= b)	0.98494	0.0000000805		
**55**	A	51	25,768	Constant	0.98259			
	B	25,769	73,700	Log10-Log10 Cubic	8.38483	-5.0609738063	1.01319	-0.0674
	C	73,701	254,587		0.50468	-0.2148067115	0.02225	
	D	254,588	442,936	Line (C1= m and C2= b)	0.98534	0.0000000779		

**56**	A	51	26,280	Constant	0.98332			
	B	26,281	74,800	Log10-Log10 Cubic	8.30592	-5.0018314423	0.99908	-0.0663
	C	74,801	260,229	Log10-Log10 Quadratic	0.51098	-0.2167404331	0.02239	
	D	260,230	451,854	Line (C1= m and C2= b)	0.98582	0.0000000740		
**57**	A	51	26,734	Constant	0.98397			
	B	26,735	76,500	Log10-Log10 Cubic	8.16592	-4.9088811922	0.97883	-0.0648
	C	76,501	263,552	Log10-Log10 Quadratic	0.51027	-0.2158201160	0.02224	
	D	263,553	458,089	Line (C1= m and C2= b)	0.98606	0.0000000717		
**58**	A	51	27,371	Constant	0.9847			
	B	27,372	77,900	Log10-Log10 Cubic	8.26716	-4.9609748182	0.98759	-0.0653
	C	77,901	267,196	Log10-Log10 Quadratic	0.51649	-0.2177068384	0.02238	
	D	267,197	466,019	Line (C1= m and C2= b)	0.98638	0.0000000685		
**59**	A	51	27,910	Constant	0.98531			
	B	27,911	79,400	Log10-Log10 Cubic	8.14568	-4.8776797063	0.96897	-0.0639
	C	79,401	272,890	Log10-Log10 Quadratic	0.51628	-0.2170096407	0.02225	
	D	272,891	475,040	Line (C1= m and C2= b)	0.98684	0.0000000651		
**60**	A	51	28,404	Constant	0.98596			
	B	28,405	80,500	Log10-Log10 Cubic	8.07343	-4.8250858639	0.9567	-0.063
	C	80,501	277,612	Log10-Log10 Quadratic	0.52625	-0.2197938338	0.02243	
	D	277,613	481,867	Line (C1= m and C2= b)	0.98725	0.0000000624		
**61**	A	51	29,038	Constant	0.98672			
	B	29,039	82,000	Log10-Log10 Cubic	8.02976	-4.7881649218	0.9473	-0.0623
	C	82,001	283,196	Log10-Log10 Quadratic	0.52807	-0.2200778579	0.02242	
	D	283,197	489,064	Line (C1= m and C2= b)	0.98768	0.0000000599		
**62**	A	51	29,466	Constant	0.98724			
	B	29,467	83,200	Log10-Log10 Cubic	7.96352	-4.7408392322	0.9364	-0.0614
	C	83,201	288,350	Log10-Log10 Quadratic	0.52694	-0.2189759548	0.02225	
	D	288,351	497,233	Line (C1= m and C2= b)	0.98814	0.0000000568		
**63**	A	51	30,085	Constant	0.98789			
	B	30,086	85,100	Log10-Log10 Cubic	7.85936	-4.6683287516	0.92004	-0.0602
	C	85,101	294,569	Log10-Log10 Quadratic	0.53231	-0.2205501273	0.02236	
	D	294,570	504,300	Line (C1= m and C2= b)	0.98858	0.0000000545		
**64**	A	51	30,657	Constant	0.98855			
	B	30,658	86,500	Log10-Log10 Cubic	7.83885	-4.6478952633	0.91445	-0.0598
	C	86,501	298,371	Log10-Log10 Quadratic	0.52993	-0.2191099262	0.02218	
	D	298,372	510,396	Line (C1= m and C2= b)	0.98886	0.0000000526		
**65**	A	51	31,106	Constant	0.98900			
	B	31,107	87,900	Log10-Log10 Cubic	7.70823	-4.5635749962	0.89653	-0.0585

	C	87,901	302,140	Log10-Log10 Quadratic	0.53295	-0.2197758341	0.0222	
	D	302,141	519,956	Line (C1= m and C2= b)	0.98912	0.0000000499		
**66**	A	51	31,658	Constant	0.98965			
	B	31,659	89,600	Log10-Log10 Cubic	7.81415	-4.6228578772	0.90766	-0.0592
	C	89,601	302,140	Log10-Log10 Quadratic	0.53306	-0.2192671932	0.0221	
	D	302,141	524,828	Line (C1= m and C2= b)	0.98918	0.0000000486		
**67**	A	51	32,182	Constant	0.99021			
	B	32,183	90,900	Log10-Log10 Cubic	7.5079	-4.4276041070	0.86647	-0.0563
	C	90,901	312,866	Log10-Log10 Quadratic	0.5126	-0.2023614945	0.01973	
	D	312,867	536,209	Line (C1= m and C2= b)	0.98991	0.0000000452		
**68**	A	51	32,698	Constant	0.9906			
	B	32,699	92,400	Log10-Log10 Cubic	7.47995	-4.4061127434	0.86133	-0.0559
	C	92,401	316,456	Log10-Log10 Quadratic	0.52987	-0.2175731016	0.0219	
	D	316,457	539,552	Line (C1= m and C2= b)	0.99021	0.0000000439		
**69**	A	51	33,239	Constant	0.99096			
	B	33,240	93,600	Log10-Log10 Cubic	7.42255	-4.3659427914	0.85227	-0.0553
	C	93,601	320,322	Log10-Log10 Quadratic	0.52894	-0.2167723751	0.02179	
	D	320,323	548,261	Line (C1= m and C2= b)	0.99044	0.0000000419		
**70**	A	51	33,652	Constant	0.99138			
	B	33,653	94,900	Log10-Log10 Cubic	7.26012	-4.2641052988	0.83118	-0.0538
	C	94,901	323,337	Log10-Log10 Quadratic	0.52708	-0.2155854502	0.02163	
	D	323,338	554,659	Line (C1= m and C2= b)	0.99061	0.0000000406		
**71**	A	51	34,267	Constant	0.99186			
	B	34,268	96,800	Log10-Log10 Cubic	7.1923	-4.2169301442	0.82058	-0.0531
	C	96,801	329,838	Log10-Log10 Quadratic	0.52351	-0.2136827375	0.0214	
	D	329,839	561,106	Line (C1= m and C2= b)	0.9911	0.0000000385		
**72**	A	51	34,813	Constant	0.99225			
	B	34,814	98,200	Log10-Log10 Cubic	7.09779	-4.1545002169	0.80707	-0.0521
	C	98,201	335,591	Log10-Log10 Quadratic	0.52197	-0.2126534244	0.02127	
	D	335,592	575,895	Line (C1= m and C2= b)	0.9915	0.0000000354		
**73**	A	51	35,370	Constant	0.9927			
	B	35,371	99,300	Log10-Log10 Cubic	7.03376	-4.1099144784	0.79705	-0.0514
	C	99,301	340,254	Log10-Log10 Quadratic	0.52539	-0.2135764048	0.02132	
	D	340,255	575,765	Line (C1= m and C2= b)	0.9918	0.0000000348		
**74**	A	51	36,018	Constant	0.9932			
	B	36,019	100,800	Log10-Log10 Cubic	6.99902	-4.0832060246	0.79067	-0.0509
	C	100,801	345,504	Log10-Log10 Quadratic	0.52529	-0.2130659515	0.02124	
	D	345,505	585,587	Line (C1= m and C2= b)	0.99221	0.0000000325		

**75**	A	51	36,522	Constant	0.99361			
	B	36,523	102,500	Log10-Log10 Cubic	6.86536	-3.9991967230	0.77326	-0.0497
	C	102,501	349,203	Log10-Log10 Quadratic	0.52159	-0.2111564225	0.02101	
	D	349,204	591,502	Line (C1= m and C2= b)	0.9924	0.0000000314		
**76**	A	51	37,039	Constant	0.99399			
	B	37,040	103,800	Log10-Log10 Cubic	6.69448	-3.8924377046	0.75121	-0.0482
	C	103,801	354,183	Log10-Log10 Quadratic	0.52094	-0.2104695232	0.02091	
	D	354,184	601,662	Line (C1= m and C2= b)	0.99269	0.0000000295		
**77**	A	51	37,546	Constant	0.99443			
	B	37,547	105,300	Log10-Log10 Cubic	6.64494	-3.8591717288	0.74398	-0.0477
	C	105,301	357,570	Log10-Log10 Quadratic	0.51858	-0.2091401787	0.02074	
	D	357,571	604,617	Line (C1= m and C2= b)	0.99294	0.0000000286		
**78**	A	51	38,206	Constant	0.99489			
	B	38,207	107,000	Log10-Log10 Cubic	6.49795	-3.7661270101	0.72457	-0.0463
	C	107,001	363,649	Log10-Log10 Quadratic	0.51757	-0.2082500928	0.02062	
	D	363,650	617,499	Line (C1= m and C2= b)	0.99332	0.0000000263		
**79**	A	51	38,724	Constant	0.99532			
	B	38,725	108,700	Log10-Log10 Cubic	6.37182	-3.6878824123	0.70856	-0.0452
	C	108,701	367,386	Log10-Log10 Quadratic	0.51291	-0.2059898343	0.02036	
	D	367,387	621,255	Line (C1= m and C2= b)	0.99356	0.0000000254		
**80**	A	51	39,257	Constant	0.9957			
	B	39,258	109,900	Log10-Log10 Cubic	6.29415	-3.6365939507	0.69748	-0.0445
	C	109,901	373,220	Log10-Log10 Quadratic	0.51513	-0.2065133822	0.02039	
	D	373,221	631,524	Line (C1= m and C2= b)	0.99394	0.0000000235		
**81**	A	51	39,828	Constant	0.99612			
	B	39,829	111,200	Log10-Log10 Cubic	6.26297	-3.6137926603	0.69222	-0.0441
	C	111,201	377,485	Log10-Log10 Quadratic	0.51609	-0.2065195574	0.02036	
	D	377,486	639,864	Line (C1= m and C2= b)	0.99424	0.0000000220		
**82**	A	51	40,445	Constant	0.99665			
	B	40,446	112,100	Log10-Log10 Cubic	6.26441	-3.6082197217	0.68996	-0.0438
	C	112,101	382,122	Log10-Log10 Quadratic	0.52572	-0.2098929314	0.02065	
	D	382,123	645,202	Line (C1= m and C2= b)	0.99454	0.0000000207		
**83**	A	51	40,908	Constant	0.99695			
	B	40,909	114,000	Log10-Log10 Cubic	5.96683	-3.4303842131	0.65471	-0.0415
	C	114,001	385,942	Log10-Log10 Quadratic	0.51611	-0.2056923349	0.02021	
	D	385,943	652,058	Line (C1= m and C2= b)	0.9947	0.0000000199		
**84**	A	51	41,421	Constant	0.99731			
	B	41,422	115,200	Log10-Log10 Cubic	5.85731	-3.3622734215	0.64074	-0.0406

	C	115,201	389,382	Log10-Log10 Quadratic	0.51654	-0.2054922329	0.02016	
	D	389,383	655,475	Line (C1= m and C2= b)	0.99489	0.0000000192		
**85**	A	51	42,021	Constant	0.99777			
	B	42,022	116,800	Log10-Log10 Cubic	5.78353	-3.3138643315	0.63036	-0.0398
	C	116,801	397,082	Log10-Log10 Quadratic	0.51653	-0.2051295229	0.0201	
	D	397,083	667,679	Line (C1= m and C2= b)	0.99543	0.0000000169		
**86**	A	51	42,608	Constant	0.99827			
	B	42,609	118,100	Log10-Log10 Cubic	5.71191	-3.2675558679	0.62058	-0.0392
	C	118,101	400,738	Log10-Log10 Quadratic	0.51954	-0.2058833134	0.02014	
	D	400,739	673,287	Line (C1= m and C2= b)	0.99566	0.0000000159		
**87**	A	51	43,252	Constant	0.99869			
	B	43,253	119,700	Log10-Log10 Cubic	5.59695	-3.1959122189	0.60587	-0.0382
	C	119,701	405,615	Log10-Log10 Quadratic	0.51916	-0.2053154653	0.02005	
	D	405,616	684,223	Line (C1= m and C2= b)	0.99594	0.0000000146		
**88**	A	51	43,713	Constant	0.99907			
	B	43,714	121,800	Log10-Log10 Cubic	5.38796	-3.0709798288	0.58112	-0.0365
	C	121,801	409,825	Log10-Log10 Quadratic	0.5126	-0.2023614945	0.01973	
	D	409,826	687,322	Line (C1= m and C2= b)	0.99615	0.0000000139		
**89**	A	51	44,281	Constant	0.99949			
	B	44,282	122,700	Log10-Log10 Cubic	5.31758	-3.0258954677	0.57167	-0.0359
	C	122,701	412,400	Log10-Log10 Quadratic	0.51762	-0.2039370947	0.01985	
	D	412,401	696,136	Line (C1= m and C2= b)	0.9963	0.0000000130		
**90**	A	51	44,987	Constant	0.99995			
	B	44,988	124,300	Log10-Log10 Cubic	5.30199	-3.0127527785	0.56842	-0.0356
	C	124,301	416,677	Log10-Log10 Quadratic	0.51964	-0.2043220234	0.01986	
	D	416,678	702,364	Line (C1= m and C2= b)	0.99657	0.0000000120		
**91**	A	51	45,470	Constant	1.0003			
	B	45,471	125,700	Log10-Log10 Cubic	5.06191	-2.8692896607	0.53999	-0.0338
	C	125,701	421,700	Log10-Log10 Quadratic	0.51717	-0.2029876934	0.0197	
	D	421,701	706,736	Line (C1= m and C2= b)	0.99682	0.0000000111		
**92**	A	51	45,860	Constant	1.00064			
	B	45,861	127,200	Log10-Log10 Cubic	4.80507	-2.7164303587	0.50981	-0.0318
	C	127,201	427,910	Log10-Log10 Quadratic	0.51405	-0.2014490218	0.01952	
	D	427,911	719,371	Line (C1= m and C2= b)	0.99716	0.0000000098		
**93**	A	51	46,543	Constant	1.00112			
	B	46,544	128,700	Log10-Log10 Cubic	4.76421	-2.6890651276	0.5039	-0.0314
	C	128,701	431,187	Log10-Log10 Quadratic	0.51694	-0.2021510738	0.01956	
	D	431,188	724,404	Line (C1= m and C2= b)	0.99735	0.0000000090		

**94**	A	51	47,048	Constant	1.0015			
	B	47,049	130,300	Log10-Log10 Cubic	4.60981	-2.5970888947	0.48576	-0.0302
	C	130,301	435,478	Log10-Log10 Quadratic	0.51295	-0.2002558995	0.01935	
	D	435,479	729,043	Line (C1= m and C2= b)	0.99763	0.0000000081		
**95**	A	51	47,590	Constant	1.00194			
	B	47,591	131,900	Log10-Log10 Cubic	4.52941	-2.5473245684	0.47562	-0.0295
	C	131,901	441,046	Log10-Log10 Quadratic	0.51258	-0.1997728719	0.01928	
	D	441,047	737,941	Line (C1= m and C2= b)	0.99797	0.0000000068		
**96**	A	51	48,189	Constant	1.00241			
	B	48,190	132,700	Log10-Log10 Cubic	4.38688	-2.4593100901	0.45769	-0.0283
	C	132,701	445,716	Log10-Log10 Quadratic	0.52324	-0.2035166958	0.01961	
	D	445,717	743,210	Line (C1= m and C2= b)	0.9982	0.0000000061		
**97**	A	51	48,721	Constant	1.00274			
	B	48,722	134,300	Log10-Log10 Cubic	4.11951	-2.3013317358	0.42672	-0.0263
	C	134,301	451,274	Log10-Log10 Quadratic	0.52083	-0.2022207466	0.01945	
	D	451,275	751,297	Line (C1= m and C2= b)	0.99848	0.0000000051		
**98**	A	51	49,260	Constant	1.00309			
	B	49,261	136,100	Log10-Log10 Cubic	3.97696	-2.2166865876	0.41008	-0.0252
	C	136,101	457,397	Log10-Log10 Quadratic	0.51606	-0.2000528899	0.01922	
	D	457,398	762,524	Line (C1= m and C2= b)	0.99883	0.0000000038		
**99**	A	51	49,872	Constant	1.00347			
	B	49,873	137,100	Log10-Log10 Cubic	3.89447	-2.1659010308	0.39979	-0.0245
	C	137,101	458,686	Log10-Log10 Quadratic	0.52107	-0.2016149260	0.01934	
	D	458,687	764,863	Line (C1= m and C2= b)	0.99885	0.0000000038		
**100**	A	51	50,466	Constant	1.00401			
	B	50,467	138,600	Log10-Log10 Cubic	3.8001	-2.1077345298	0.38799	-0.0237
	C	138,601	464,514	Log10-Log10 Quadratic	0.52555	-0.2029655287	0.01944	
	D	464,515	775,268	Line (C1= m and C2= b)	0.99922	0.0000000025		
**101**	A	51	50,940	Constant	1.00436			
	B	50,941	466,383	Log10-Log10 Cubic	3.68304	-2.0388595083	0.37459	-0.0229
	C	466,384	779,185	Line (C1= m and C2= b)	0.99793	0.0000000066		
**102**	A	51	51,479	Constant	1.00469			
	B	51,480	470,459	Log10-Log10 Cubic	3.50742	-1.9353228578	0.35436	-0.0215
	D	470,460	787,993	Line (C1= m and C2= b)	0.99821	0.0000000056		
**103**	A	51	52,126	Constant	1.00517			
	B	52,127	475,743	Log10-Log10 Cubic	3.37018	-1.8532160896	0.33812	-0.0205
	C	475,744	791,354	Line (C1= m and C2= b)	0.99857	0.0000000045		
**104**	A	51	52,691	Constant	1.00561			

	B	52,692	481,410	Log10-Log10 Cubic	3.20418	-1.7543260275	0.31861	-0.0192
	D	481,411	799,404	Line (C1= m and C2= b)	0.99894	0.0000000033		
**105**	A	51	53,307	Constant	1.00598			
	B	53,308	485,652	Log10-Log10 Cubic	3.03169	-1.6530714656	0.29893	-0.0179
	C	485,653	806,287	Line (C1= m and C2= b)	0.99921	0.0000000025		
**106**	A	51	53,985	Constant	1.00649			
	B	53,986	492,716	Log10-Log10 Cubic	2.97155	-1.6143284675	0.29078	-0.0174
	D	492,717	813,671	Line (C1= m and C2= b)	0.99967	0.0000000010		
**107**	A	51	54,349	Constant	1.00679			
	B	54,350	495,613	Log10-Log10 Cubic	2.64157	-1.4246620667	0.2546	-0.0151
	C	495,614	823,601	Line (C1= m and C2= b)	0.9999	0.0000000003		
**108**	A	51	55,055	Constant	1.00729			
	B	55,056	501,529	Log10-Log10 Cubic	2.4058	-1.2861637866	0.22764	-0.0133
	D	501,530	829,938	Line (C1= m and C2= b)	1.00028	-0.0000000008		
**109**	A	51	55,663	Constant	1.00729			
	B	55,664	507,110	Log10-Log10 Cubic	2.44434	-1.3070106312	0.23141	-0.0136
	C	507,111	833,015	Line (C1= m and C2= b)	1.00027	-0.0000000008		
**110**	A	51	56,092	Constant	1.00805			
	B	56,093	512,027	Log10-Log10 Cubic	2.08966	-1.1016563185	0.19195	-0.0111
	D	512,028	842,529	Line (C1= m and C2= b)	1.00097	-0.0000000029		
**111**	A	51	56,737	Constant	1.00855			
	B	56,738	514,519	Log10-Log10 Cubic	1.91349	-0.9975894506	0.17161	-0.0097
	C	514,520	847,529	Line (C1= m and C2= b)	1.00116	-0.0000000035		
**112**	A	51	57,273	Constant	1.00885			
	B	57,274	521,729	Log10-Log10 Cubic	1.71257	-0.8805040568	0.14897	-0.0083
	D	521,730	860,157	Line (C1= m and C2= b)	1.00158	-0.0000000047		
**113**	A	51	57,808	Constant	1.00928			
	B	57,809	525,322	Log10-Log10 Cubic	1.60704	-0.8187515723	0.13703	-0.0075
	C	525,323	863,992	Line (C1= m and C2= b)	1.00182	-0.0000000054		
**114**	A	51	58,351	Constant	1.00978			
	B	58,352	525,735	Log10-Log10 Cubic	1.41992	-0.7094195331	0.11587	-0.0062
	D	525,736	870,703	Line (C1= m and C2= b)	1.00189	-0.0000000055		
**115**	A	51	58,992	Constant	1.0102			
	B	58,993	536,310	Log10-Log10 Cubic	1.18247	-0.5726831283	0.08975	-0.0045
	C	536,311	882,203	Line (C1= m and C2= b)	1.00252	-0.0000000073		
