# Supplementary figures and images for: A Comparison of 100 Human Genes Using an Alu Element-Based Instability Model
Source: PLoS One. 2013 Jun 3;8(6):e65188. doi: 10.1371/journal.pone.0065188 (PMC3670932; doi:10.1371/journal.pone.0065188)

# Ectopic Invasion and Annealing of Complementary DNA Replication Forks

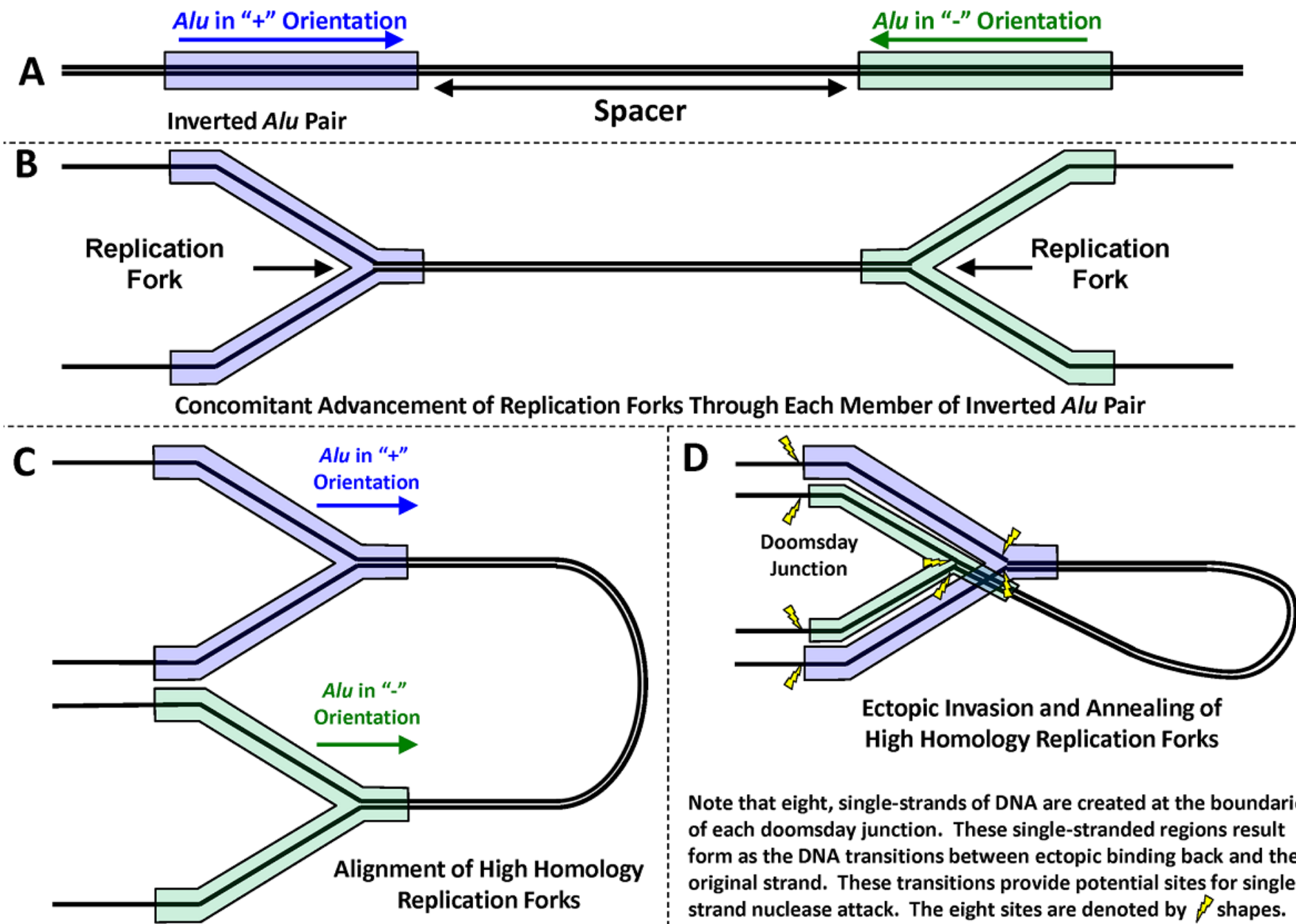

Supplement: Figure S1 — A proposed mechanism for the formation of a doomsday junction that is catalyzed by the ectopic invasion and annealing of complementary replication forks. (A) Two Alu elements in opposite orientations form an inverted Alu pair. (B) Concomitant advancement of replication forks through each member of an inverted Alu pair. C) Bending of the DNA to permit alignment of the complementary replication forks. D) Ectopic invasion and annealing of single-stranded DNA associated between high-homology replication forks could potentially extend to the entire length of the Alu elements. The hypothetical conformation created by this interaction is termed a doomsday junction. As also illustrated in Figure 1, eight segments of single-stranded DNA are formed at the boundary of the doomsday junction and create the opportunity for single-strand nuclease attack. These sites are illustrated as yellow lightning bolts. (PDF) [file pone.0065188.s007.pdf]

## *Alu* Pair I:D Ratio vs Type 1, 2 and 3 *Alu* Pairs

*(actual vs regressed data for large-large *Alu* pairs, 275-375 bp)*

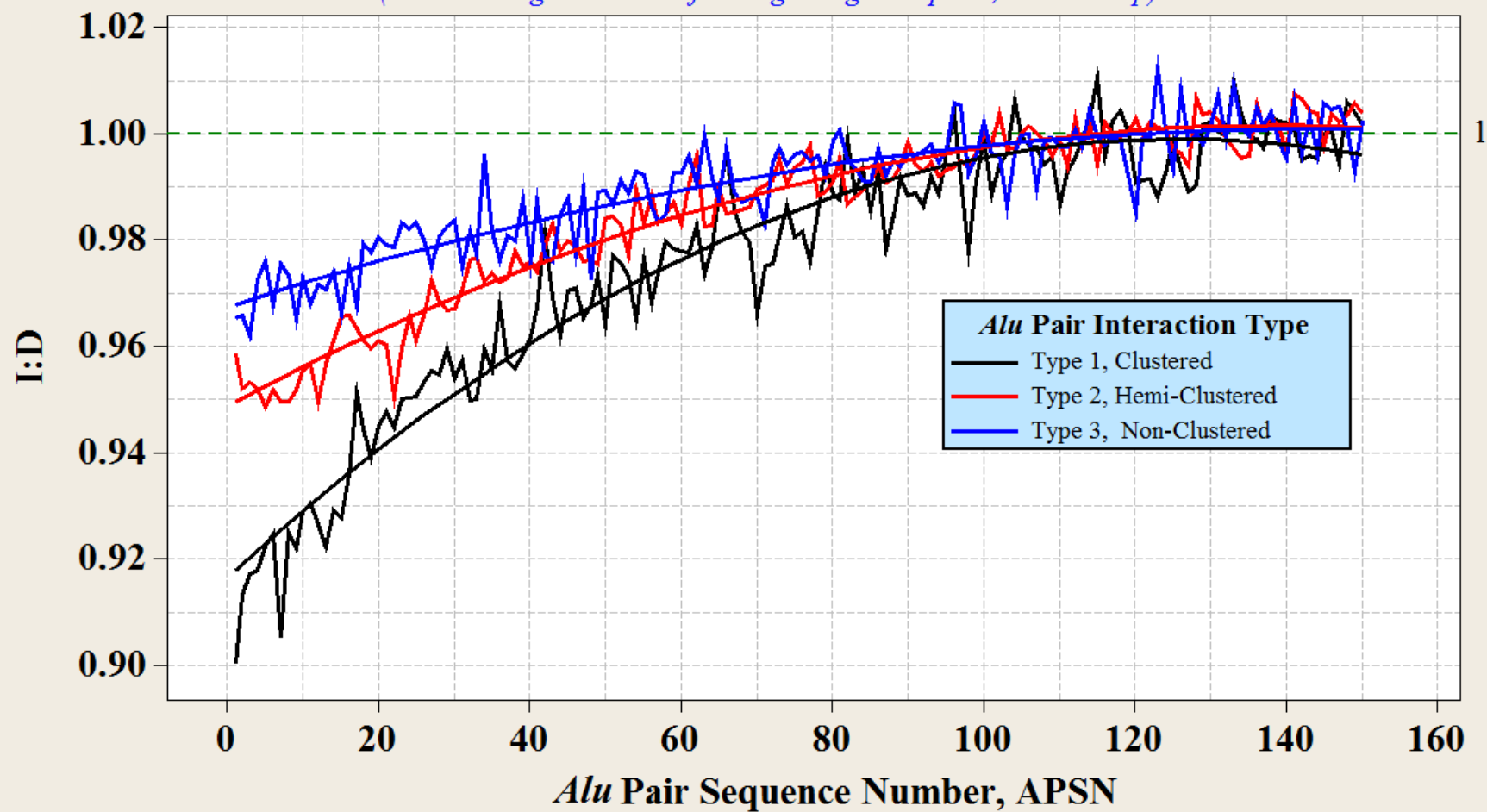

Supplement: Figure S3 — I∶D ratios for Type 1, 2 and 3 Alu pair families for APSNs 1–150. Note that the departure of the I∶D ratio from unity is greatest for clustered (Type 1) Alu pairs and closest to unity for non-clustered (Type 3) Alu pairs. (PDF) [file pone.0065188.s009.pdf]

# Deletion-Prone Cancer Gene Stabilities

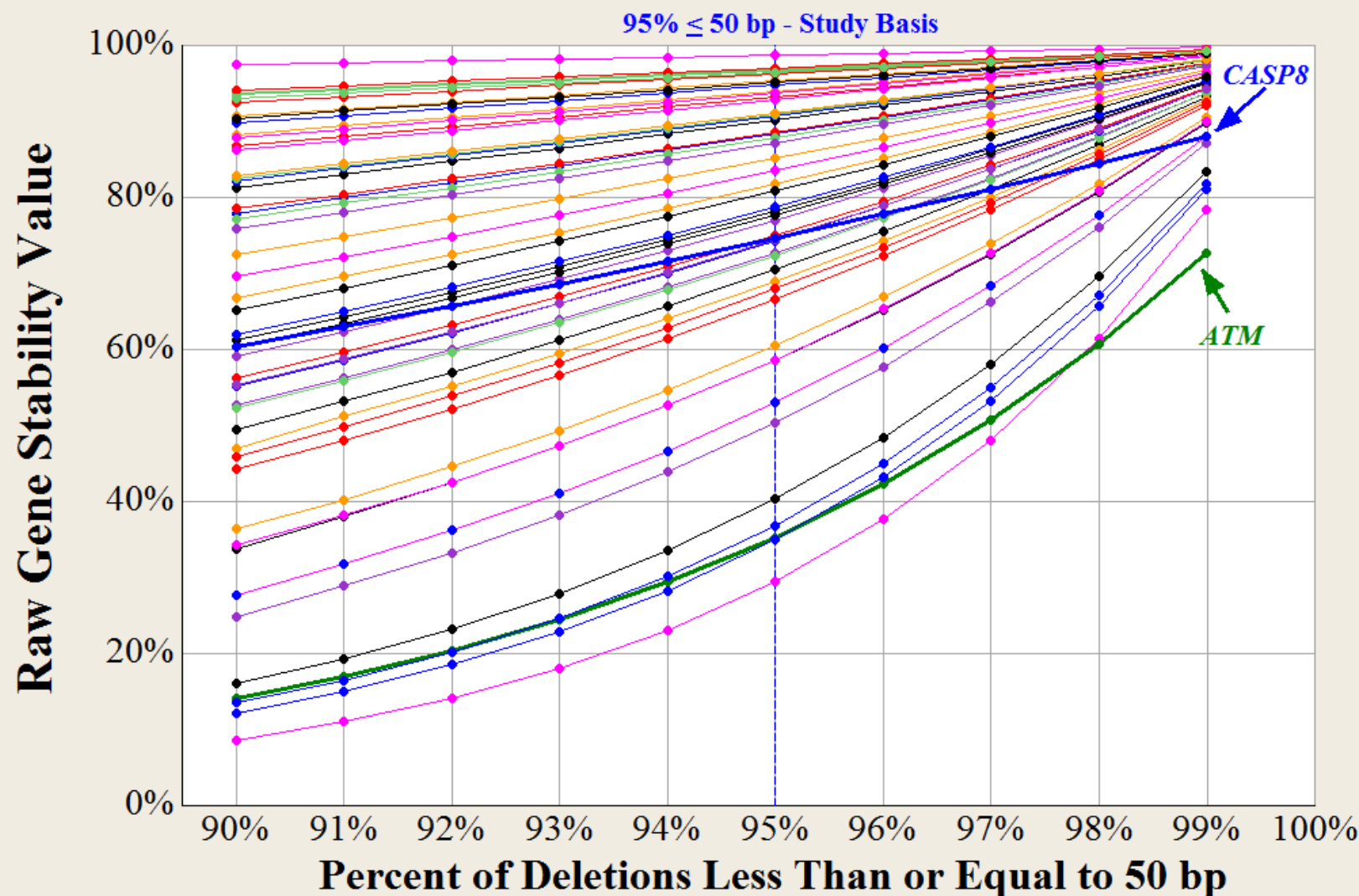

Supplement: Figure S5 — Sensitivity analysis of the relative stabilities of the deletion-prone cancer genes versus variation in the fraction of deletions that is ≤50 bp. The shape of the deletion size frequency curve used to determine relative gene stabilities (Figure 5) places 95% of deletions with lengths of ≤50 bp. This figure examines the variation in relative deletion-prone cancer gene stabilities as the fraction of deletions ≤50 bp is varied between 90% and 99%. As can be seen from this figure, the relative stabilities of 48 of the 50 deletion-prone cancer genes (96%) remain essentially unchanged as the ≤50 bp increment is varied. Exceptions to this observation are observed with ATM and CASP8 (bolded curves), which have the rare occurrence of Alus within 5 and 7 bp of their exons, respectively. These two genes exhibit higher relative stabilities as the fraction of deletions ≤50 bp in length increases. (PDF) [file pone.0065188.s011.pdf]
