# Supplementary material for: A Comparison of 100 Human Genes Using an Alu Element-Based Instability Model
Source: PLoS One. 2013 Jun 3;8(6):e65188. doi: 10.1371/journal.pone.0065188 (PMC3670932; doi:10.1371/journal.pone.0065188)
Supplement: Figure S2 — The Alu pair I∶D ratio versus spacer size for Type 2 and Type 3 Alu pairs for APSNs ±1–110. This figure illustrates the ±110 APSN curves for full-length (275–325 bp) A) Type 2 Alu pairs and B) Type 3 human Alu pairs. (PDF) [file pone.0065188.s008.pdf]

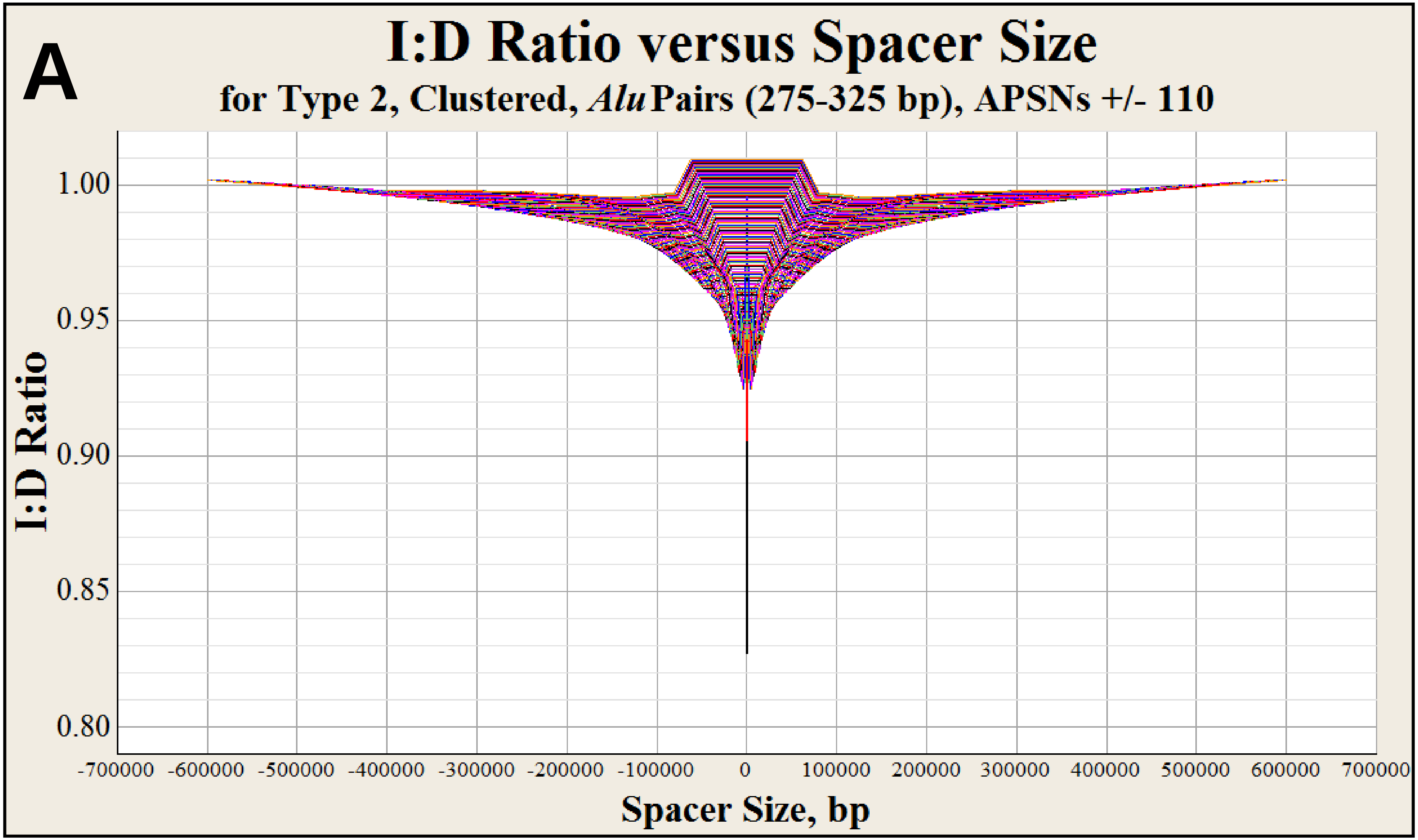
A
I:D Ratio versus Spacer Size
for Type 2, Clustered, Alu Pairs (275-325 bp), APSNs +/- 110
I:D Ratio
1.00
0.95
0.90
0.85
0.80
-700000
-600000
-500000
-400000
-300000
-200000
-100000
0
100000
200000
300000
400000
500000
600000
700000
Spacer Size, bp

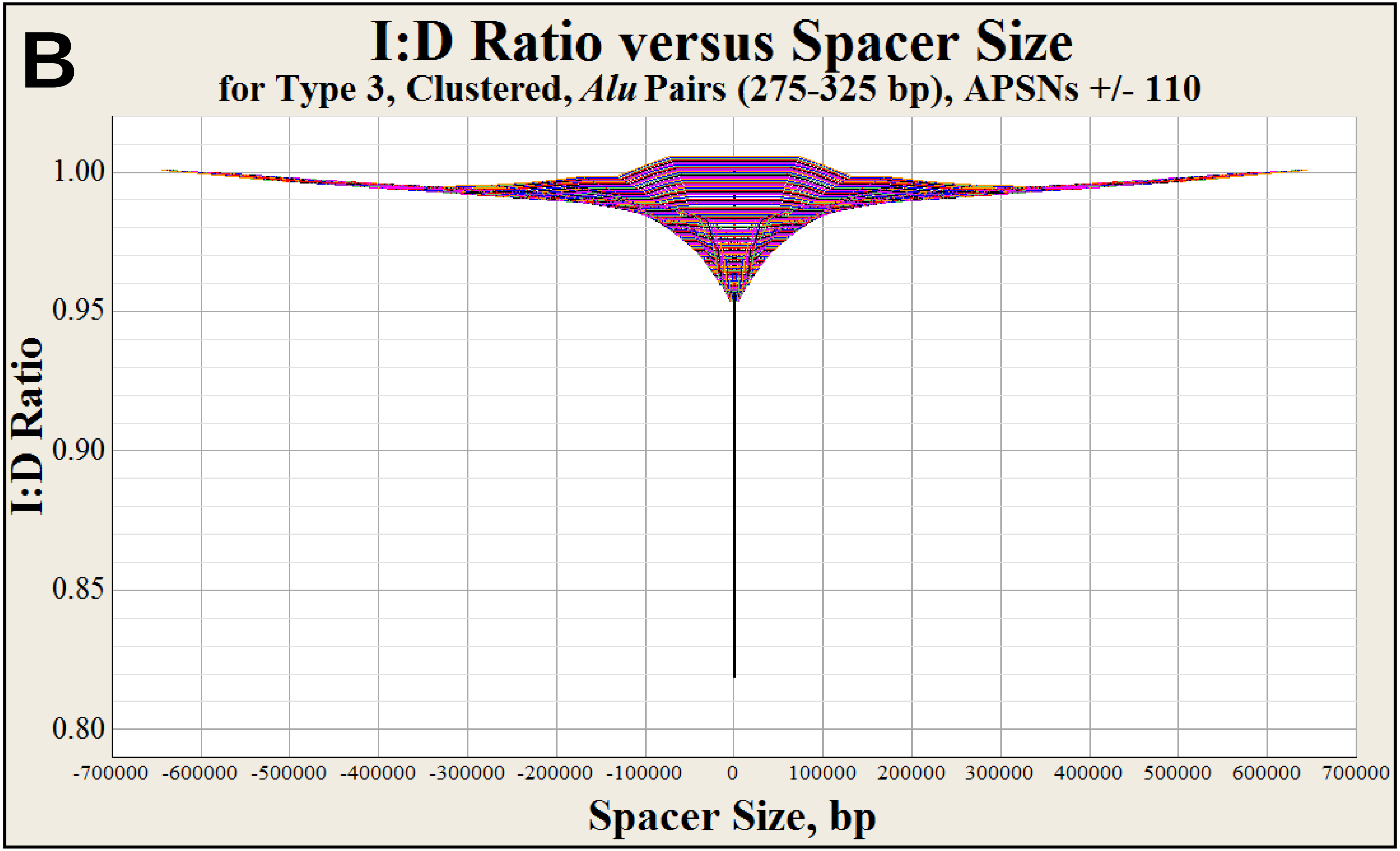
B
I:D Ratio versus Spacer Size
for Type 3, Clustered, *Alu* Pairs (275-325 bp), APSNs +/- 110
I:D Ratio
1.00
0.95
0.90
0.85
0.80
-700000
-600000
-500000
-400000
-300000
-200000
-100000
0
100000
200000
300000
400000
500000
600000
700000
Spacer Size, bp
