# Supplementary material for: A Comparison of 100 Human Genes Using an Alu Element-Based Instability Model
Source: PLoS One. 2013 Jun 3;8(6):e65188. doi: 10.1371/journal.pone.0065188 (PMC3670932; doi:10.1371/journal.pone.0065188)
Supplement: Figure S4 — Alu landscapes for five deletion-prone cancer genes and three randomly chosen genes. Each Alu element is plotted within and 500 kbp, 5′ and 3′ flanking each gene. The locus of each Alu is plotted against its respective instability score, iScore. The iScore is the inverse of the model's predicted Alu stability and thus larger values represent higher instabilities. The five selected deletion-prone cancer genes are A) APC, B) ATM, C) MLH1, D) MSH2 and E) TP53. The three randomly chose genes are F) GDPD2, G) KEAP1 and H) SF3B3. (PDF) [file pone.0065188.s010.pdf]

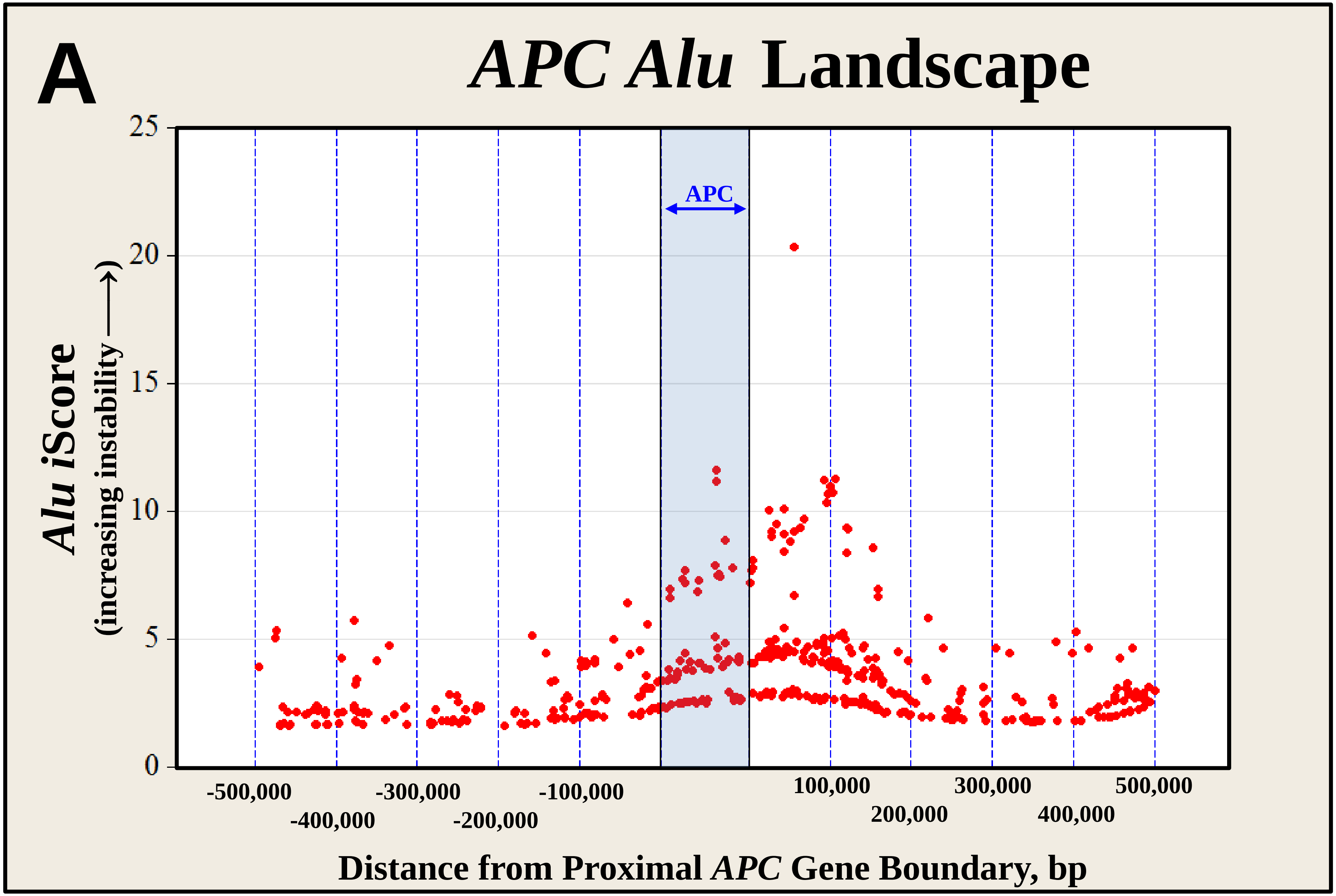
A
APC Alu Landscape
Alu iScore
(increasing instability ⟶)
25
20
15
10
5
0
APC
-500,000
-400,000
-300,000
-200,000
-100,000
100,000
200,000
300,000
400,000
500,000
Distance from Proximal APC Gene Boundary, bp

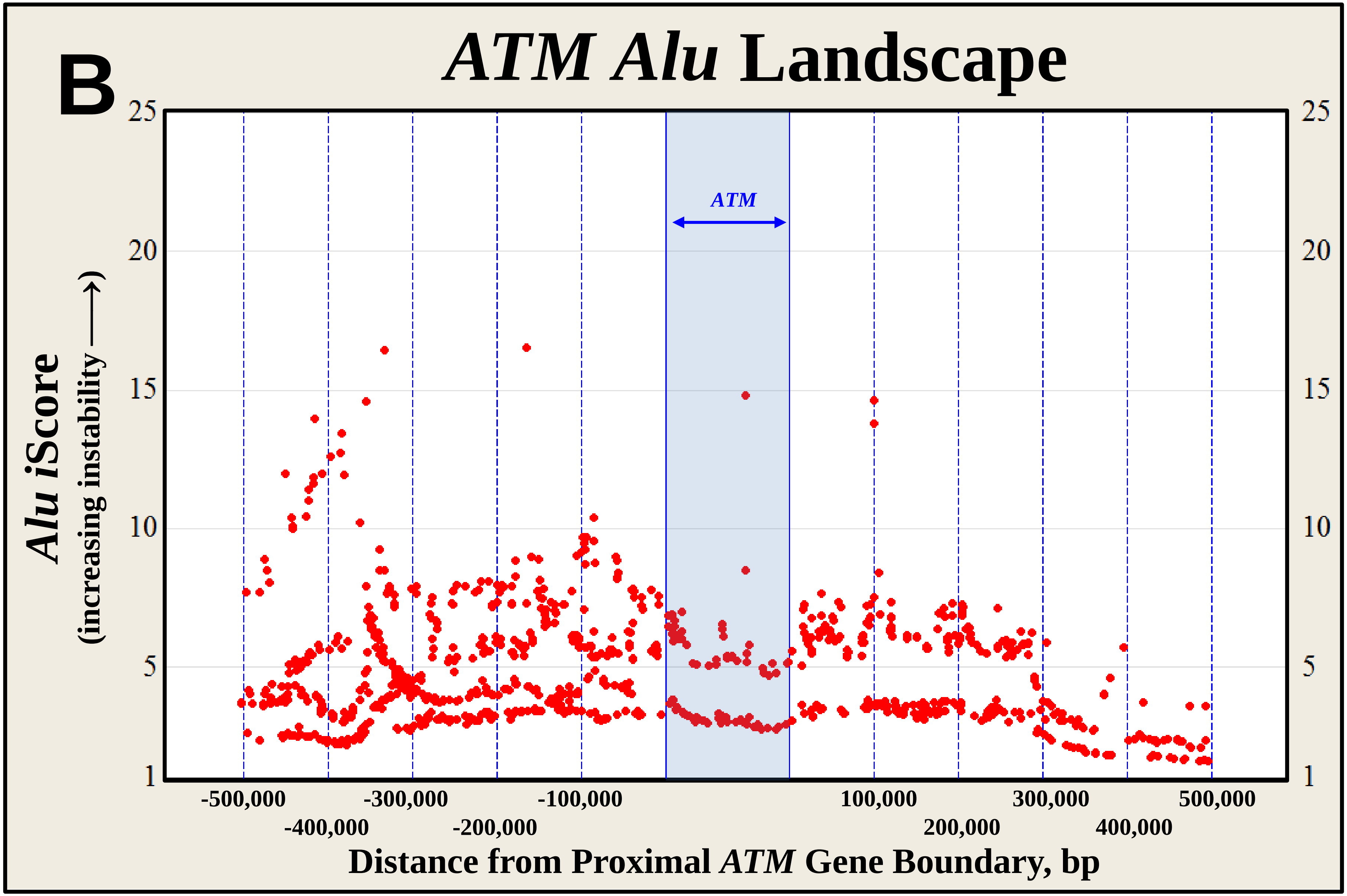
B
ATM Alu Landscape
ATM
Alu iScore
(increasing instability ⟶)
25
20
15
10
5
1
-500,000
-400,000
-300,000
-200,000
-100,000
100,000
200,000
300,000
400,000
500,000
Distance from Proximal ATM Gene Boundary, bp

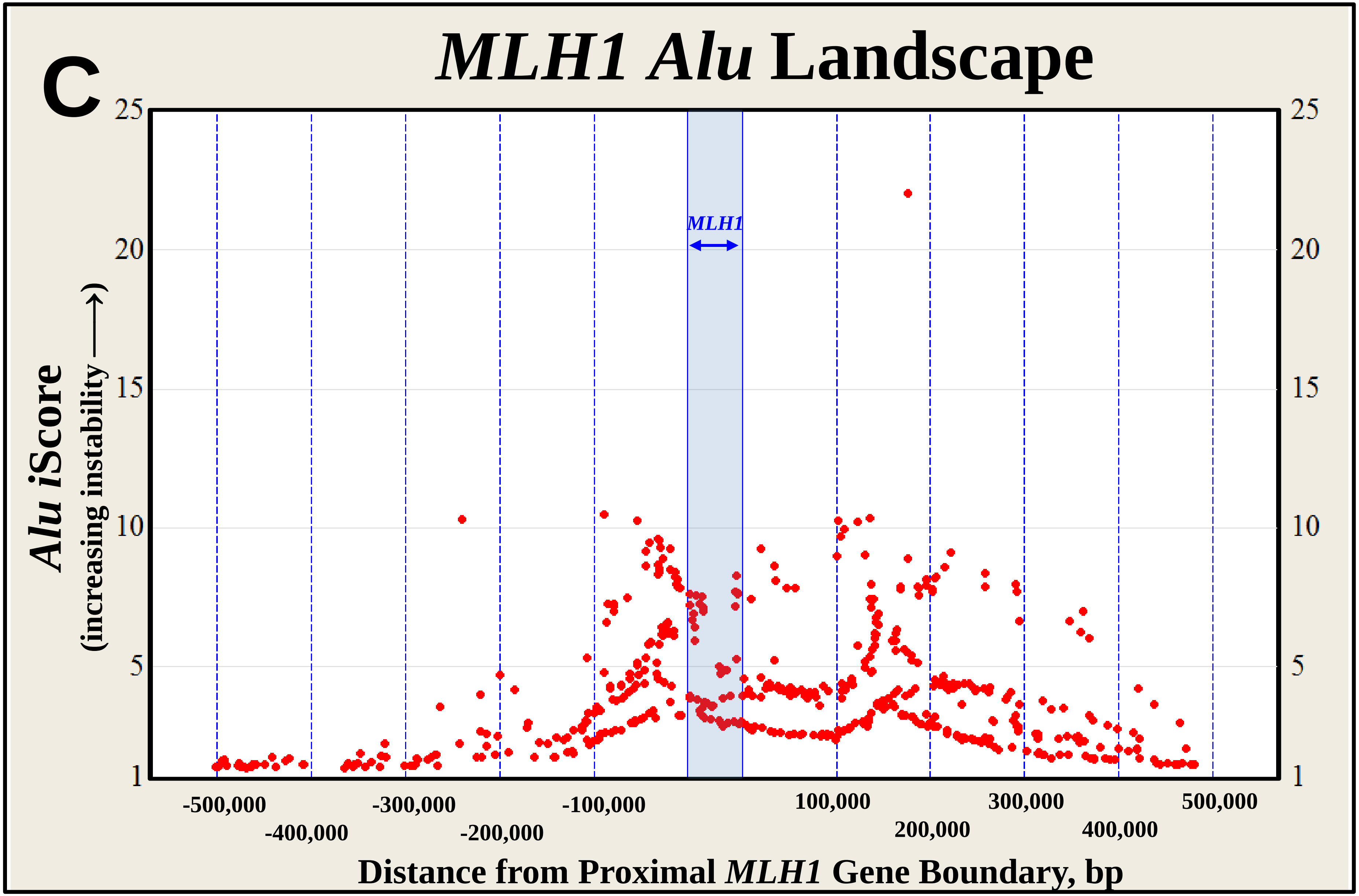
C
MLH1 Alu Landscape
MLH1
Alu iScore
(increasing instability ⟶)
25
20
15
10
5
1
-500,000
-400,000
-300,000
-200,000
-100,000
100,000
200,000
300,000
400,000
500,000
Distance from Proximal MLH1 Gene Boundary, bp

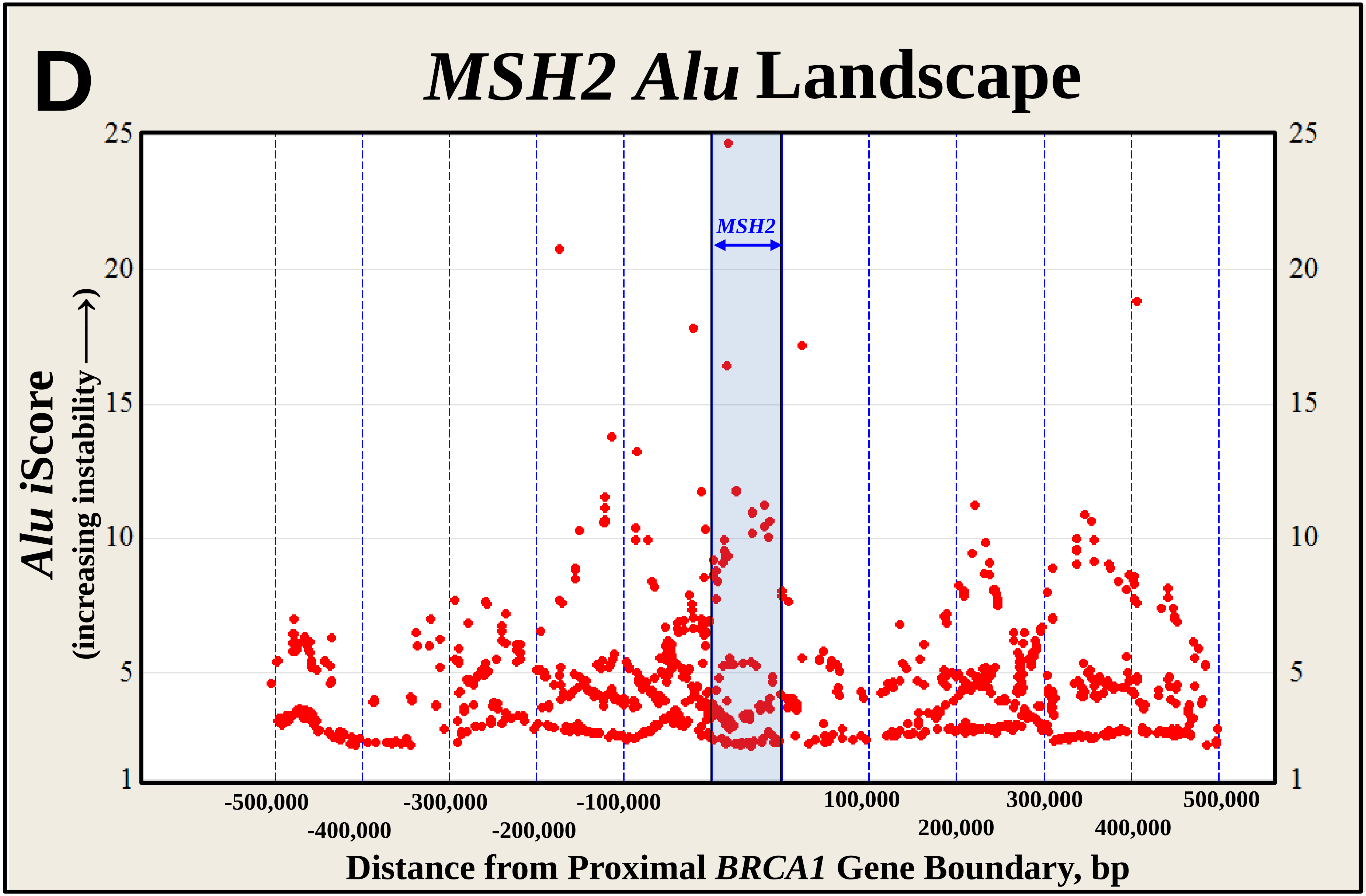
D
MSH2 Alu Landscape
Alu iScore
(increasing instability ⟶)
MSH2
25
20
15
10
5
1
-500,000
-400,000
-300,000
-200,000
-100,000
100,000
200,000
300,000
400,000
500,000
Distance from Proximal BRCA1 Gene Boundary, bp

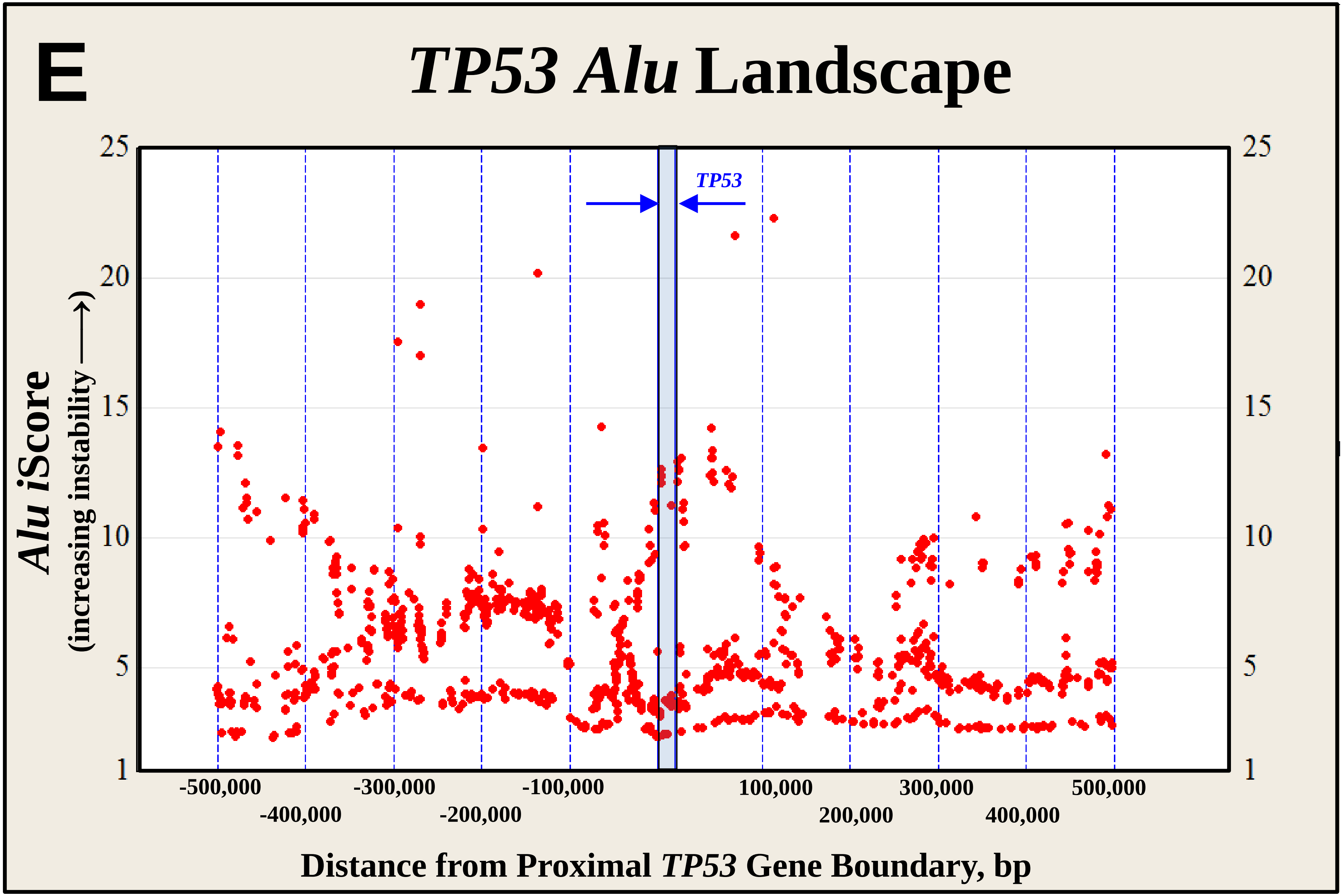
E
TP53 Alu Landscape
TP53
Alu iScore
(increasing instability ⟶)
25
20
15
10
5
1
-500,000
-400,000
-300,000
-200,000
-100,000
100,000
200,000
300,000
400,000
500,000
Distance from Proximal TP53 Gene Boundary, bp

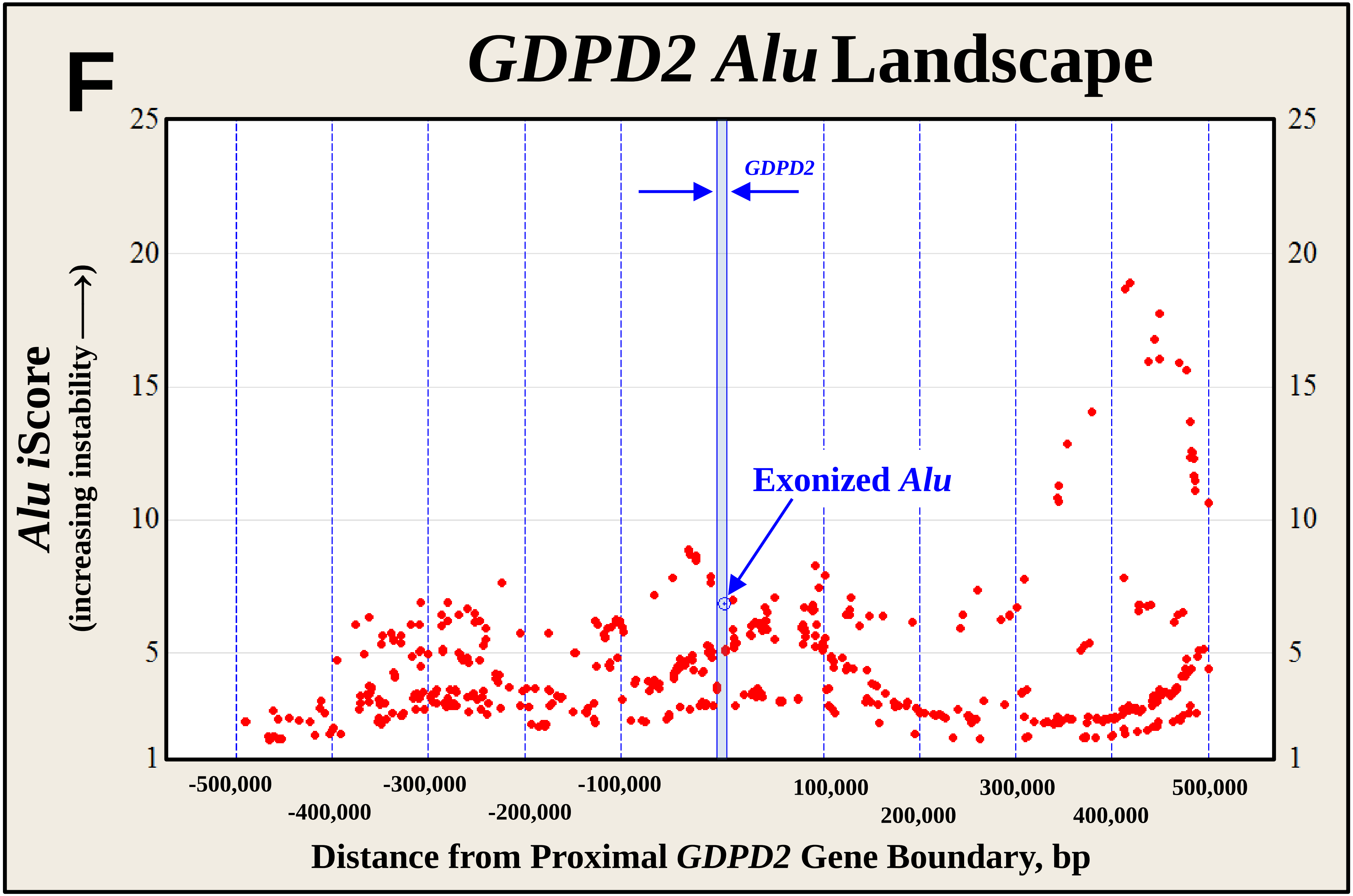
F
GDPD2 Alu Landscape
Alu iScore
(increasing instability ⟶)
25
20
15
10
5
1
GDPD2
Exonized Alu
-500,000
-400,000
-300,000
-200,000
-100,000
100,000
200,000
300,000
400,000
500,000
Distance from Proximal GDPD2 Gene Boundary, bp

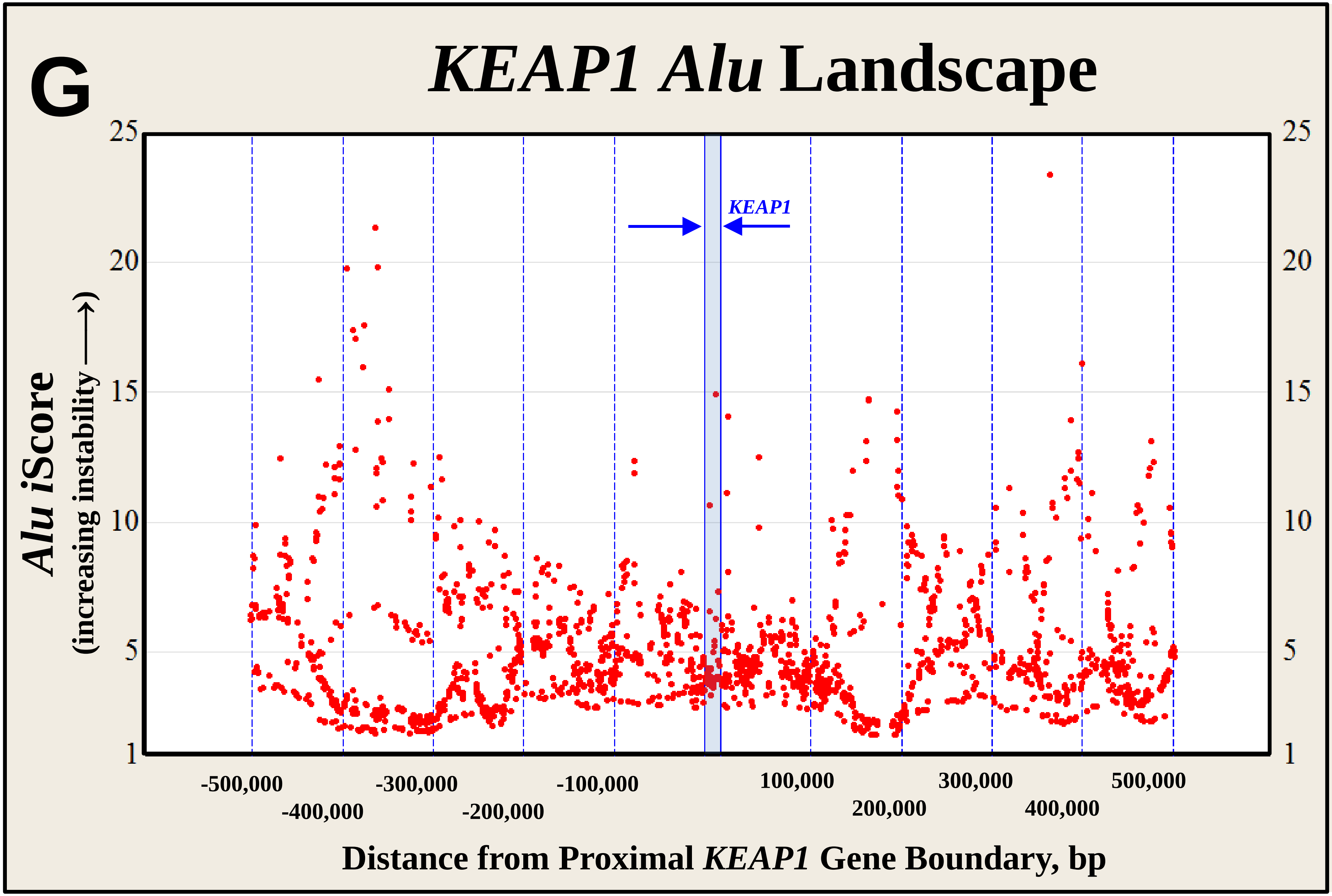
G
KEAP1 Alu Landscape
KEAP1
Alu iScore
(increasing instability ⟶)
25
20
15
10
5
1
-500,000
-400,000
-300,000
-200,000
-100,000
100,000
200,000
300,000
400,000
500,000
Distance from Proximal KEAP1 Gene Boundary, bp

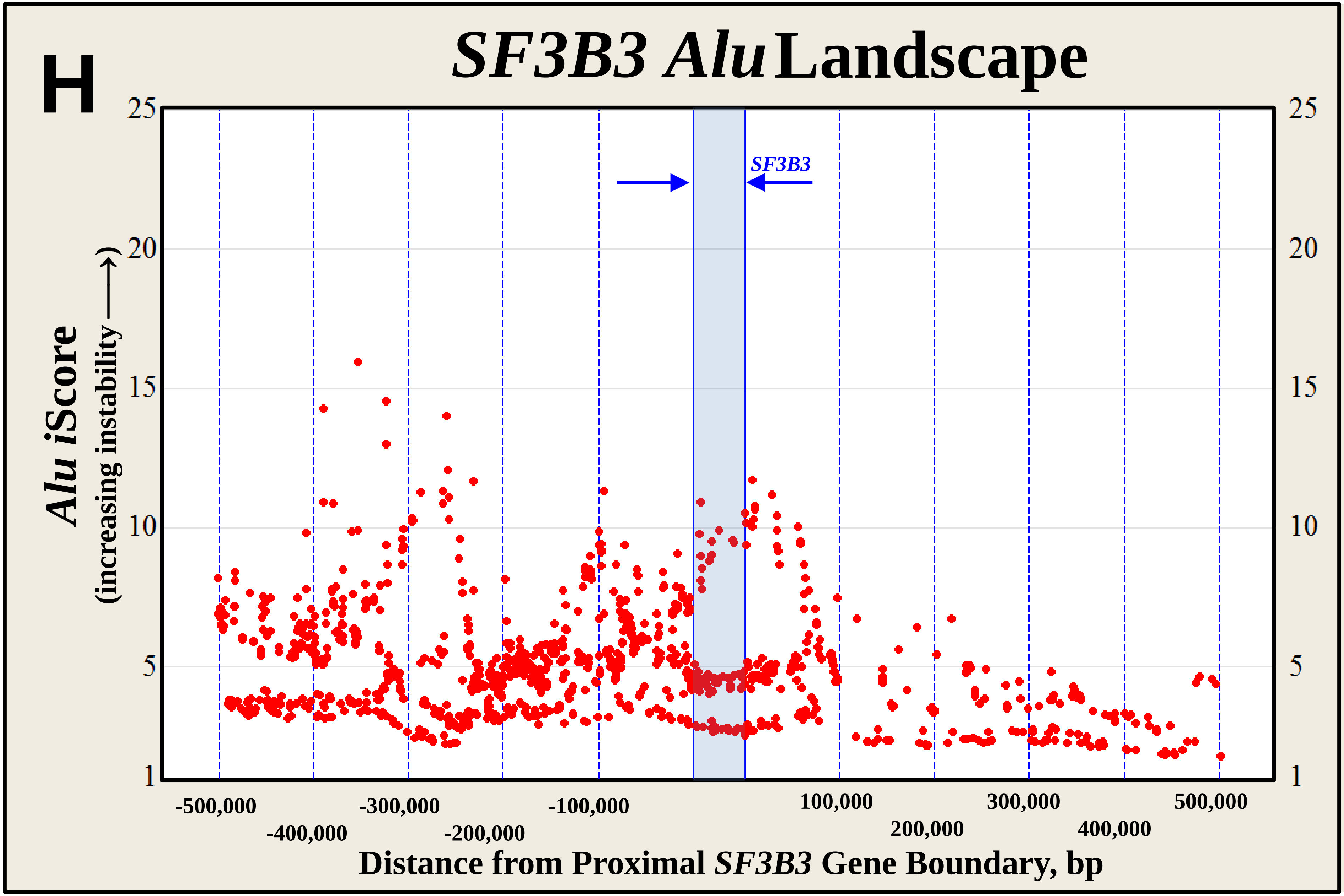
H
SF3B3 Alu Landscape
Alu iScore
(increasing instability ⟶)
25
20
15
10
5
1
SF3B3
-500,000
-400,000
-300,000
-200,000
-100,000
100,000
200,000
300,000
400,000
500,000
Distance from Proximal SF3B3 Gene Boundary, bp
