# Supplementary material for: A Comparison of 100 Human Genes Using an Alu Element-Based Instability Model
Source: PLoS One. 2013 Jun 3;8(6):e65188. doi: 10.1371/journal.pone.0065188 (PMC3670932; doi:10.1371/journal.pone.0065188)
Supplement: Figure S6 — Estimated relative exon stability distributions for the 50 deletion-prone cancer genes and 50 randomly chosen genes. A) Boxplot of the individual exon stabilities for the 50 deletion-prone cancer genes. The genes in this figure are ordered left-to-right on the basis of each gene's least stable exon. While individual exon stabilities vary widely, they tend to cluster in a gene specific manner. Exceptions to this pattern are illustrated by the presence of a single, outlying low stability exon within ATM and CASP8. These individual exon stabilities place these two genes at first and second place of highest instability among these 50 deletion-prone cancer genes. (B) Boxplot of the individual exon stabilities for the 50 randomly selected genes. Note that a broken Y-axis scale is required to capture the low stability of the putative exonized Alu in the 12th exon of GDPD2 (see text). (PDF) [file pone.0065188.s012.pdf]

A Relative Exon Stability Distributions, Deletion-Prone Genes

(plotted in order of most unstable exon, left-to-right)

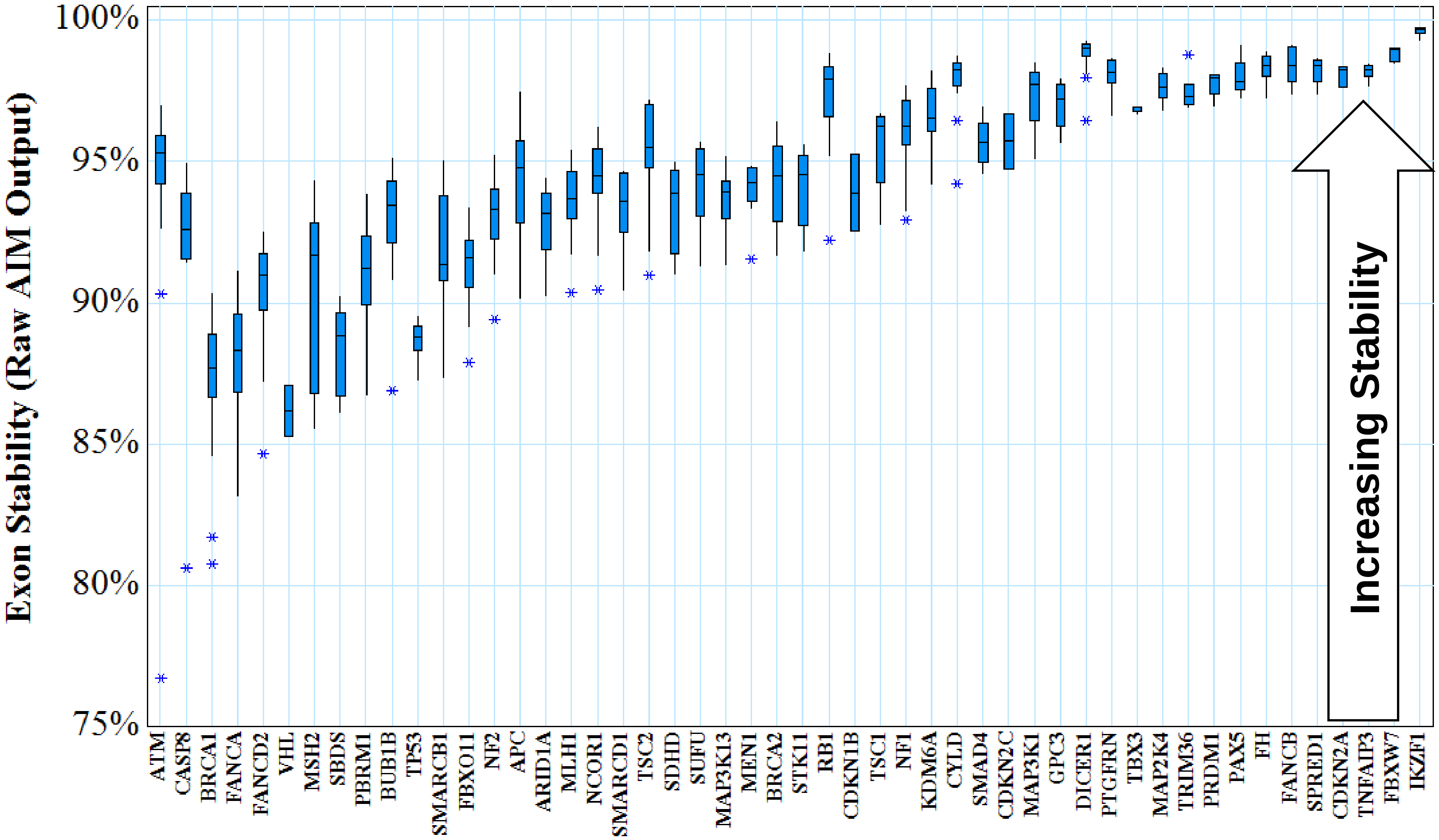

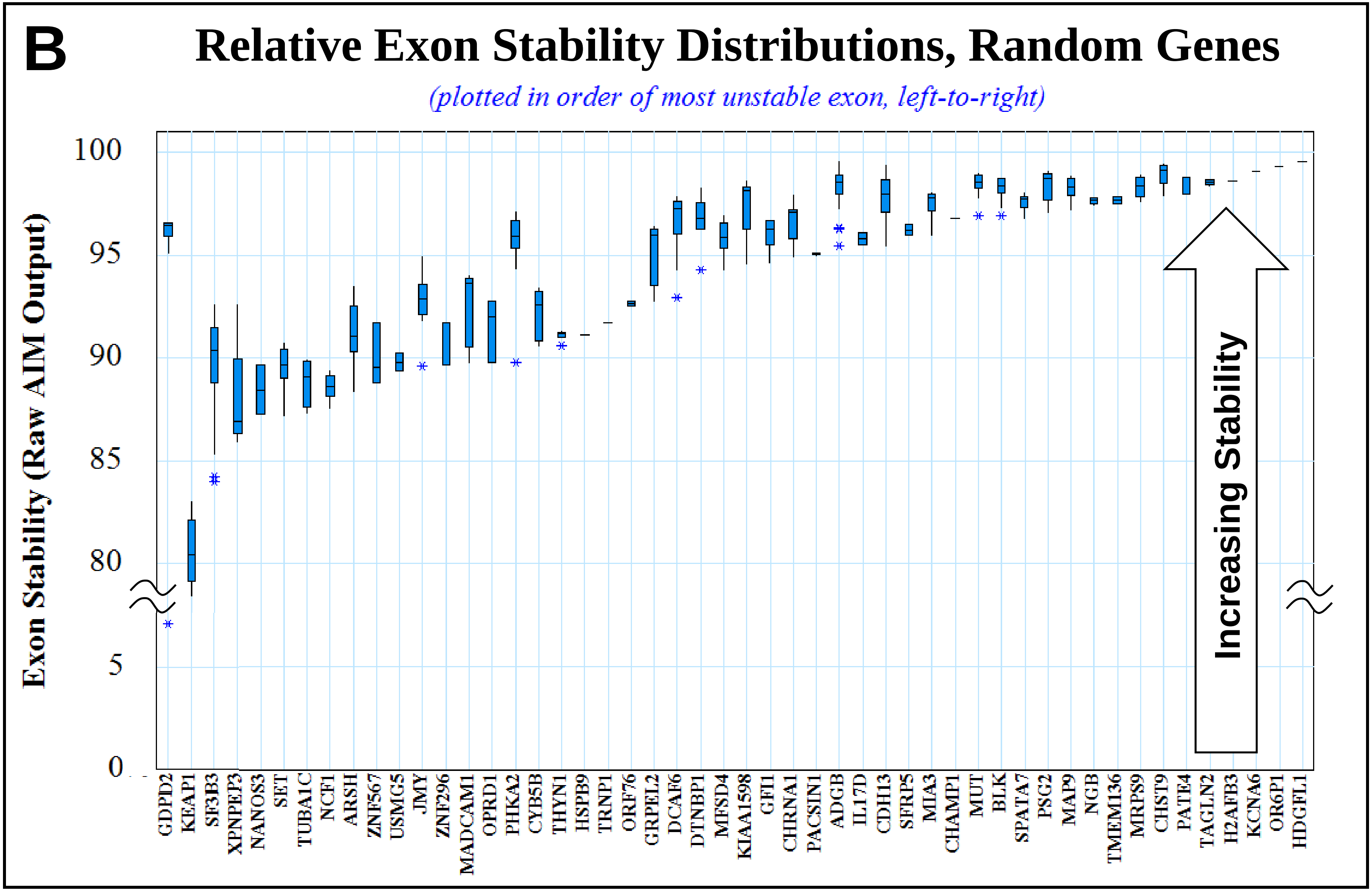

B
Relative Exon Stability Distributions, Random Genes
(plotted in order of most unstable exon, left-to-right)
Exon Stability (Raw AIM Output)
100
95
90
85
80
5
0
Increasing Stability
GDPD2
KEAP1
SF3B3
XPNPEP3
NANOS3
SET
TUBA1C
NCF1
ARSH
ZNF567
USMG5
JMY
ZNF296
MADCAM1
OPRD1
PHKA2
CYB5B
THYN1
HSPB9
TRNP1
ORF76
GRPEL2
DCAF6
DTNBP1
MFSD4
KIAA1598
GFI1
CHRNA1
PACSIN1
ADGB
IL17D
CDH13
SFRP5
MIA3
CHAMP1
MUT
BLK
SPATA7
PSG2
MAP9
NGB
TMEM136
MRPS9
CHST9
PATE4
TAGLN2
H2AFB3
KCNA6
OR6P1
HDGFL1
