# Supplementary material for: A Comparison of 100 Human Genes Using an Alu Element-Based Instability Model
Source: PLoS One. 2013 Jun 3;8(6):e65188. doi: 10.1371/journal.pone.0065188 (PMC3670932; doi:10.1371/journal.pone.0065188)
Supplement: Figure S7 — Fitted curves for 2.5th percentile spacer size groupings for type 1, 2 and 3 for APSN families 1–115. These three curves are part of thirty curves that are used to estimate the I∶D ratio for the Type 1, Type 2 and Type 3 Alu pairs. Ten different curves are used for each Alu Pair Type. These ten curves are used to construct an I∶D ratio curve for each APSN family versus spacer size (see Methods). The curves shown here for A) Type 1 B) Type 2 and C) Type 3 Alu pairs represent the I∶D ratio for the smallest median spacer size percentile (2.5th percentile) of spacer size groupings. (PDF) [file pone.0065188.s013.pdf]

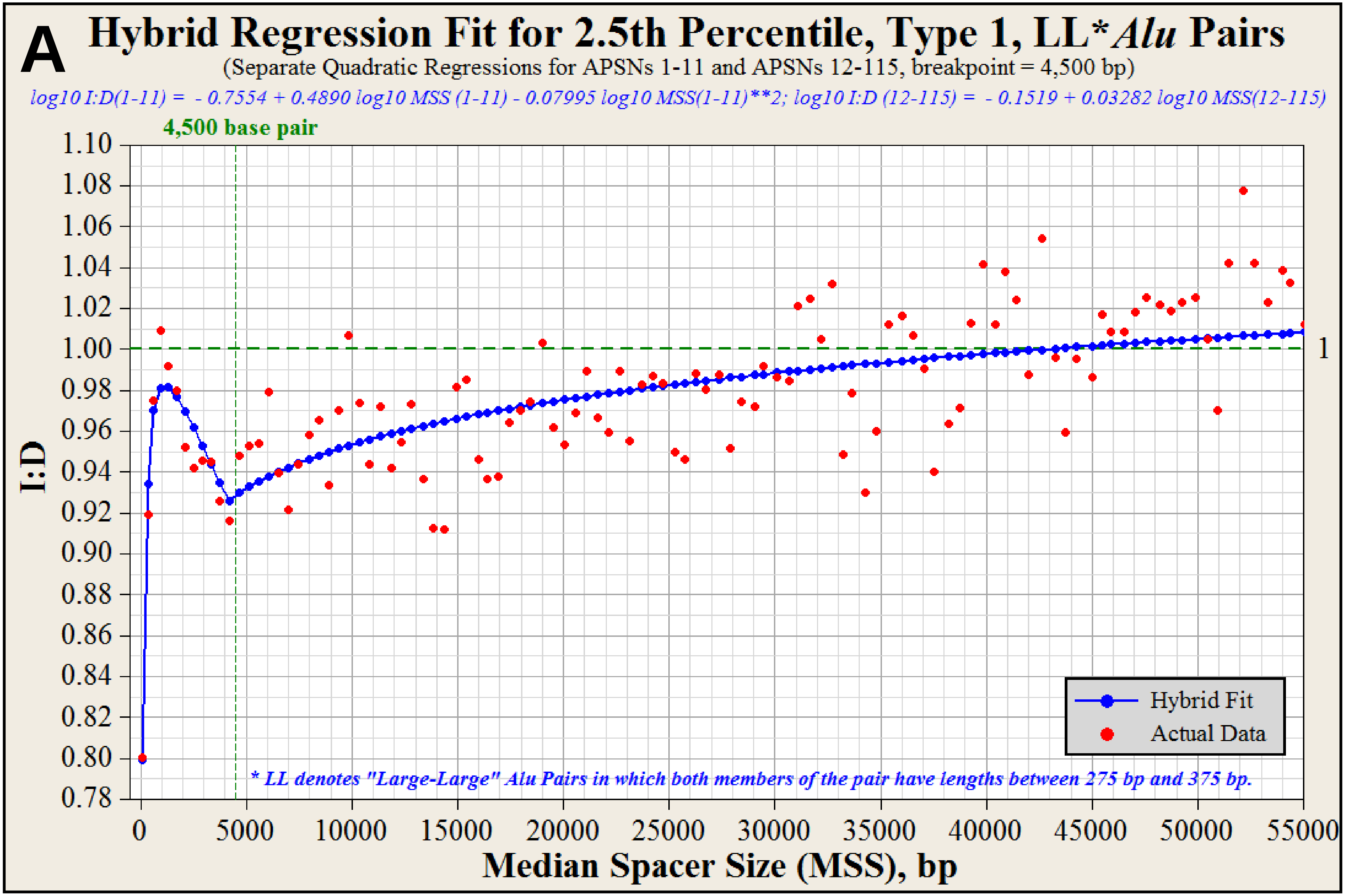
A
Hybrid Regression Fit for 2.5th Percentile, Type 1, LL*Alu Pairs
(Separate Quadratic Regressions for APSNs 1-11 and APSNs 12-115, breakpoint = 4,500 bp)
log10 I:D(1-11) = - 0.7554 + 0.4890 log10 MSS (1-11) - 0.07995 log10 MSS(1-11)**2; log10 I:D (12-115) = - 0.1519 + 0.03282 log10 MSS(12-115)
4,500 base pair
I:D
1.10
1.08
1.06
1.04
1.02
1.00
0.98
0.96
0.94
0.92
0.90
0.88
0.86
0.84
0.82
0.80
0.78
1
0
5000
10000
15000
20000
25000
30000
35000
40000
45000
50000
55000
Median Spacer Size (MSS), bp
Hybrid Fit
Actual Data
* LL denotes "Large-Large" Alu Pairs in which both members of the pair have lengths between 275 bp and 375 bp.

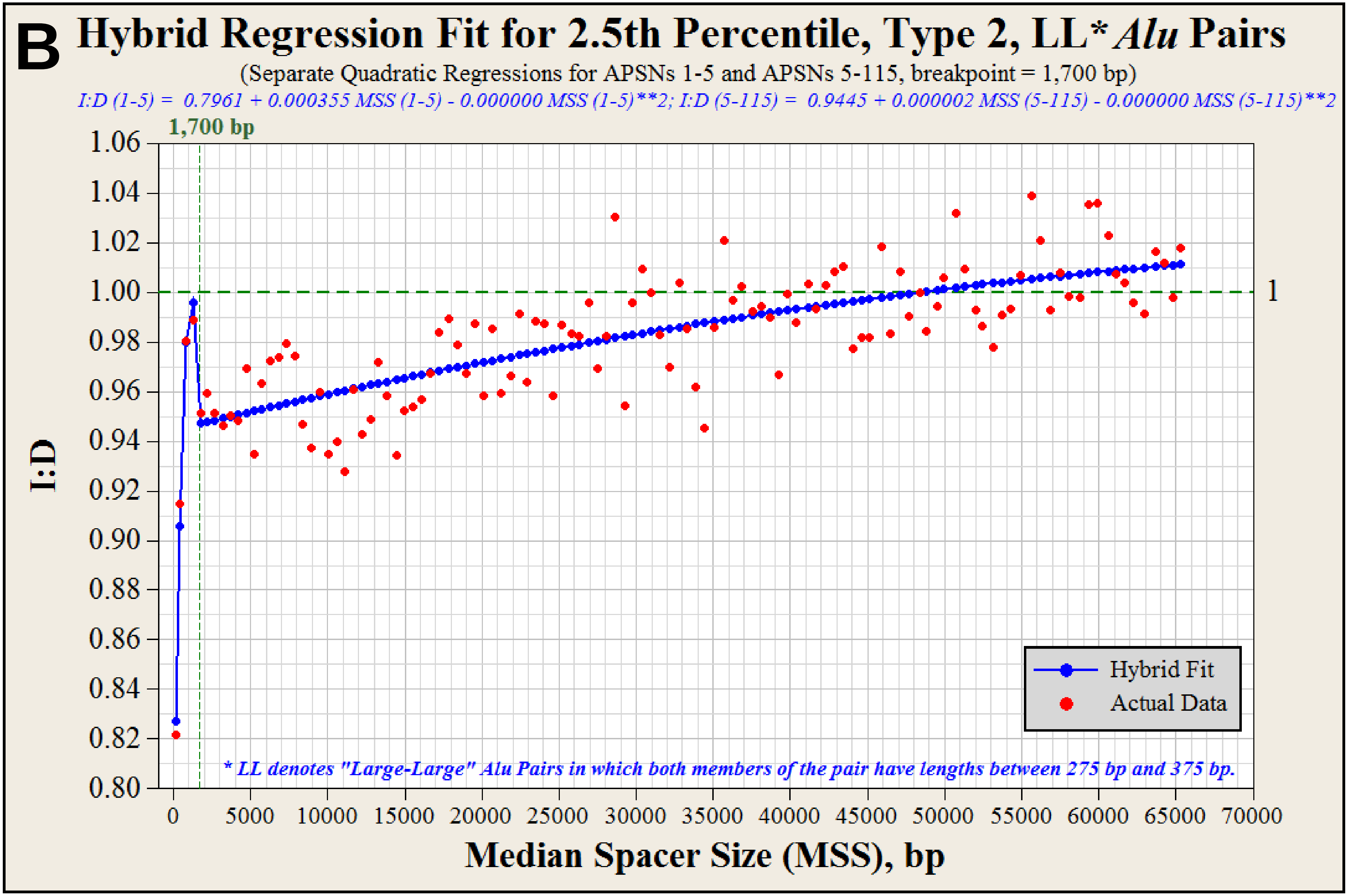
B
Hybrid Regression Fit for 2.5th Percentile, Type 2, LL* Alu Pairs
(Separate Quadratic Regressions for APSNs 1-5 and APSNs 5-115, breakpoint = 1,700 bp)
I:D (1-5) = 0.7961 + 0.000355 MSS (1-5) - 0.000000 MSS (1-5)**2; I:D (5-115) = 0.9445 + 0.000002 MSS (5-115) - 0.000000 MSS (5-115)**2
1,700 bp
1
I:D
Median Spacer Size (MSS), bp
Hybrid Fit
Actual Data
* LL denotes "Large-Large" Alu Pairs in which both members of the pair have lengths between 275 bp and 375 bp.
1.06
1.04
1.02
1.00
0.98
0.96
0.94
0.92
0.90
0.88
0.86
0.84
0.82
0.80
0
5000
10000
15000
20000
25000
30000
35000
40000
45000
50000
55000
60000
65000
70000

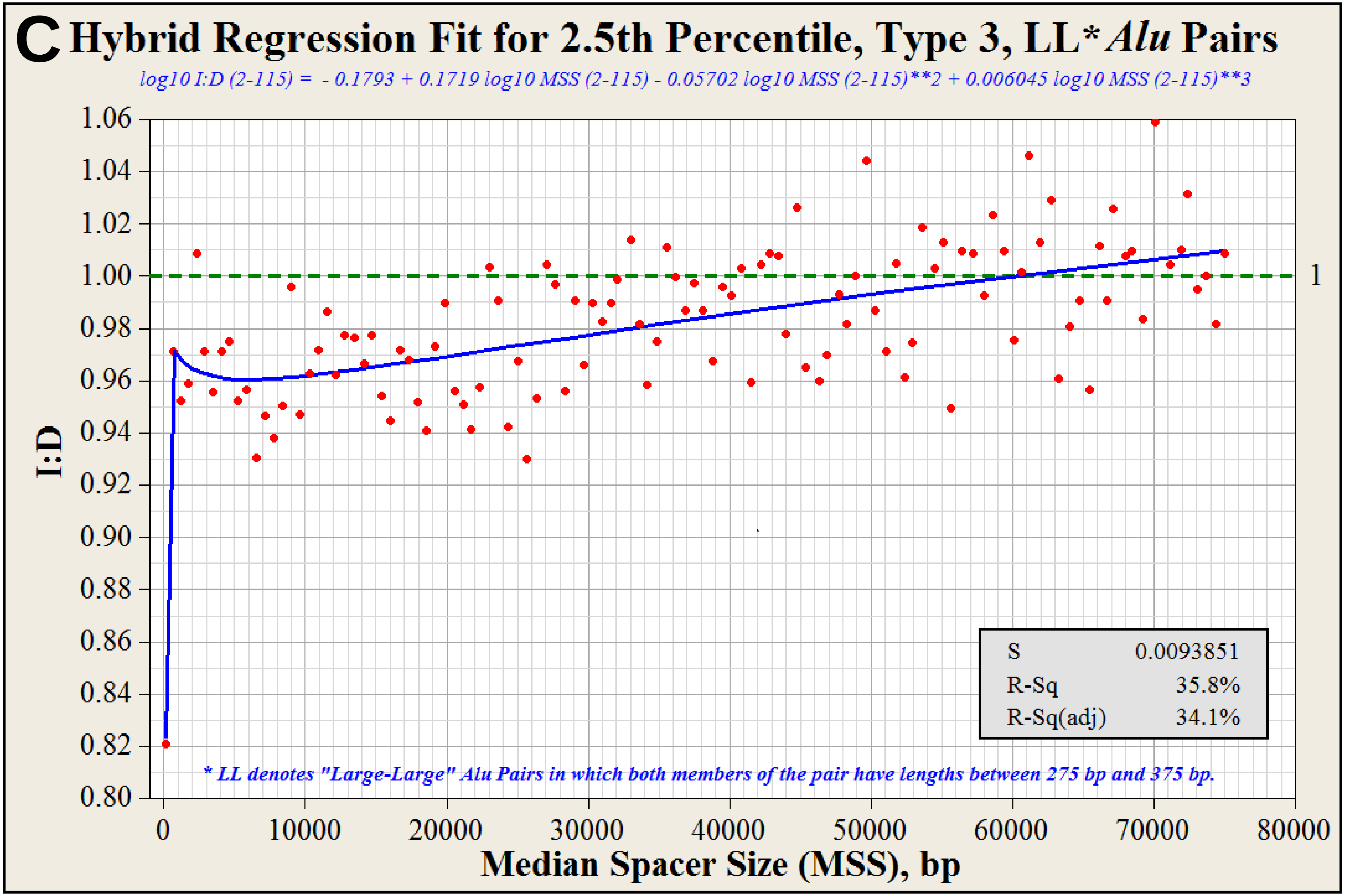
C Hybrid Regression Fit for 2.5th Percentile, Type 3, LL* Alu Pairs
log10 I:D (2-115) = - 0.1793 + 0.1719 log10 MSS (2-115) - 0.05702 log10 MSS (2-115)**2 + 0.006045 log10 MSS (2-115)**3
I:D
1.06
1.04
1.02
1.00
0.98
0.96
0.94
0.92
0.90
0.88
0.86
0.84
0.82
0.80
1
S 0.0093851
R-Sq 35.8%
R-Sq(adj) 34.1%
* LL denotes "Large-Large" Alu Pairs in which both members of the pair have lengths between 275 bp and 375 bp.
0
10000
20000
30000
40000
50000
60000
70000
80000
Median Spacer Size (MSS), bp
